# A systematic review and meta-analysis of pathogen reduction in onsite sanitation systems

**DOI:** 10.1016/j.wroa.2023.100171

**Published:** 2023-02-21

**Authors:** Isaac G. Musaazi, Shane McLoughlin, Heather M. Murphy, Joan B. Rose, Nynke Hofstra, Innocent K. Tumwebaze, Matthew E. Verbyla

**Affiliations:** a Department of Civil, Construction, and Environmental Engineering, San Diego State University, San Diego, CA 92182, United States; b Department of Epidemiology and Biostatistics, College of Public Health, Temple University, Philadelphia, PA, United States; c Department of Fisheries and Wildlife, Michigan State University, East Lansing, MI, United States; d Water Systems and Global Change Group, Wageningen University, the Netherlands

**Keywords:** Fecal sludge, Pit latrines, Composting toilets, Urine-diverting toilets, Septic tanks

## Abstract

The safe management of fecal sludge from the 3.4 billion people worldwide that use onsite sanitation systems can greatly reduce the global infectious disease burden. However, there is limited knowledge about the role of design, operational, and environmental factors on pathogen survival in pit latrines, urine diverting desiccation toilets, and other types of onsite toilets. We conducted a systematic literature review and meta-analysis to characterize pathogen reduction rates in fecal sludge, feces, and human excreta with respect to pH, temperature, moisture content, and the use of additives for desiccation, alkalinization, or disinfection. A meta-analysis of 1,382 data points extracted from 243 experiments described in 26 articles revealed significant differences between the decay rates and T_99_ values of pathogens and indicators from different microbial groups. The overall median T_99_ values were 4.8 days, 29 days, >341 days, and 429 days for bacteria, viruses, protozoan (oo)cysts, and *Ascaris* eggs, respectively. As expected, higher pH values, higher temperatures, and the application of lime all significantly predicted greater pathogen reduction rates but the use of lime by itself was more effective for bacteria and viruses than for *Ascaris* eggs, unless urea was also added. In multiple lab-scale experiments, the application of urea with enough lime or ash to reach a pH of 10 – 12 and a sustained concentration of 2,000 – 6,000 mg/L of non-protonated NH_3—_N reduced *Ascaris* eggs more rapidly than without urea. In general, the storage of fecal sludge for 6 months adequately controls hazards from viruses and bacteria, but much longer storage times or alkaline treatment with urea and low moisture or heat is needed to control hazards from protozoa and helminths. More research is needed to demonstrate the efficacy of lime, ash, and urea in the field. More studies of protozoan pathogens are also needed, as very few qualifying experiments were found for this group.

## Highlights

• Protozoa and *Ascaris* persisted longer in fecal sludge than bacteria and viruses.
• Median T_99_ values for bacteria and viruses were 4.8 and 29 days, respectively.
• Median T_99_ values for protozoa and *Ascaris* were >341 and 429 days, respectively.
• Pathogen type, design and operational factors explained 67% of variability in decay.
• Lime/ash treatment accelerates decay of *Ascaris*, but possibly only with urea present.

## Graphical abstract

**Figure:**
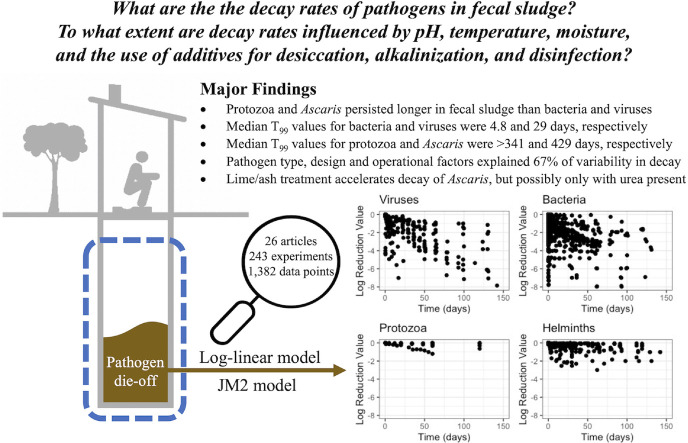

## 1 Introduction

Pathogens in human excreta create public health risks if they are released to the environment (Rose and Jiménez-Cisneros, 2020). Globally, 3.4 billion people (∼31% of urban residents and ∼58% of rural residents) utilize onsite sanitation technologies such as pit latrines, composting latrines, urine-diversion toilets, cesspits, or septic systems (Joint Monitoring Program (JMP) WHO/UNICEF, 2020). As of 2020, 78% of the world's population had at least basic sanitation services, but only 54% had safely managed sanitation (WHO/UNICEF, 2020). In 2017, diarrheal diseases attributed to enteric bacterial and viral pathogens caused 1.6 million deaths globally, with children being the most affected (Dadonaite et al., 2018). Onsite sanitation can provide an effective barrier to pathogen transmission (Mehl et al., 2011). However, when pits and tanks fill up with sludge, their contents must be emptied and conveyed to a treatment facility, unless there is room to excavate a new pit or tank (and cover/bury the old one). Manual pit emptiers often experience skin infections and gastrointestinal diseases (Farling et al., 2019; Chumo et al., 2021). There is a need to provide risk-based recommendations for storage times in onsite sanitation facilities to protect the health of pit emptiers and other people who are exposed to fecal sludge (e.g., from illegal dumping).

In pit latrines, composting toilets, and septic tanks, fecal sludge is stored onsite (often for years) before the pits/tanks fill up. During this time, if the toilet is still in use, newer pathogens may be added, but older pathogens die-off or decay. The survival of different pathogens in fecal sludge varies greatly (Austin and Cloete 2008; Chien et al., 2001; Jensen et al., 2009; Magri et al., 2013; Mensah et al., 2013). Helminths and protozoa generally survive longer than bacteria and viruses (Jimenez, 2007). Nevertheless, there is a lack of understanding about the decay rates of different pathogens in fecal sludge and about the impact of design, operational, and environmental factors on pathogen survival. Pathogen decay is often assumed to follow pseudo-first order kinetics, based on Chick's law (Chick, 1908), while biphasic and other decay models have also been considered (Fidjeland et al., 2013; Niwagaba et al., 2009). Mitchell and Akram (2019) found that the nonlinear JM2 model (Juneja et al., 2006), which allows for shouldering (initial delay in decay) and tailing (decay slowing with time), provided the best fit for modeling the decay of pathogens and fecal indicators in wastewater, manure, and biosolids.

The design, operation, and maintenance of onsite sanitation systems directly influences the characteristics of the fecal sludge, which subsequently influences pathogen survival. For example, toilet type (flush vs. dry vs. urine diversion toilets) influences moisture content, which affects pathogen survival (Mehl et al., 2011). Temperature, moisture, and pH also affect the rate of pathogen decay (Koné et al., 2007; Darimani et al., 2015; Chien et al., 2001), and can be controlled by applying additives for desiccation and/or alkalinization, such as soil, ash, or quicklime. There have been inconsistent recommendations about what fecal sludge characteristics should be achieved to promote faster pathogen decay. For example, some authors have recommended moisture levels below 25% to achieve rapid pathogen decay in pit latrines (Mehl et al., 2011), while others have recommended lower moisture levels of 3 – 5% (Dey et al., 2016; Endale et al., 2012; Nordin, 2010). Pathogens decay more rapidly at higher temperatures, and the temperature of fecal sludge can be increased through composting or solar heating—however, the efficacy of thermal disinfection is dependent on pathogen type and influenced by matrix effects (Espinosa et al., 2020). For viruses, heat treatment causes nonoxidative denaturation of capsid proteins, affecting host cell binding and resulting in a loss of viability (Wigginton et al., 2012). For protozoan pathogens such as *Cryptosporidium*, exposure to elevated temperatures depletes carbohydrate energy reserves, preventing excystation (Peng et al., 2008). Better knowledge about pathogen decay rates can help ensure safely managed sanitation systems (Rose et al., 2019; Mraz et al., 2021).

There have been inconsistent recommendations and unanswered questions about the hazards associated with fecal sludge emptying. For example, some toilets are designed with dual pits to allow for stabilization and treatment prior to removal, but there are inconsistent recommendations about the storage time required before fecal sludge can be safely removed and reused (e.g., for land application). Austin and Cloete (2008) promoted storage for 9 to 12 months to treat fecal sludge in urine diversion toilets. Kumwenda et al. (2019) recommended a storage time of one year or more for ecological sanitation systems in Malawi. Others have suggested six months (Chien et al., 2001) or even as low as three to four months (Jensen et al., 2009), but *E. coli* and *A. lumbricoides* have been detected in composting latrine contents even after 14 months of undisturbed storage (Dey et al., 2016). The recommended storage times and conditions to produce fecal sludge that can be safely reused can be improved with a better understanding about the kinetics of pathogen inactivation. Other factors that influence risk are the number of users of the sanitation facility and the prevalence of disease among the users. For example, if the contents of the pit or tank are well mixed, then any pathogens present in the “fresh” feces will be diluted out with older material that has already experienced more decay (Fleming 2017).

Predictive microbiology involves forecasting pathogen survival using models that predict inactivation based on influencing factors (e.g., temperature and time). This approach has been previously used in the food industry (Kadoya et al., 2019), and has also been applied for waste stabilization ponds (Verbyla et al., 2017), sunlight-mediated pathogen decay (Nelson et al., 2018), and for free chlorine disinfection (Kadoya et al., 2019). To our knowledge, predictive microbiology has not yet been successfully applied for the management of onsite sanitation systems (Sherpa et al., 2009). There would be great value in the development of a predictive model for pathogen decay in onsite sanitation systems, which could be combined with dynamic temporal and spatial pathogen flow models to better quantify risks associated with emptying or discharging fecal sludge and excreta to the environment (e.g., Hofstra et al., 2013; Hofstra and Vermeulen, 2016; Fleming, 2017; Kiulia et al., 2015; Mills et al., 2018; 2019; Foster et al., 2021). Previous reviews of pathogen survival in onsite sanitation systems (e.g., Bicudo and Goyal, 2003; Feachem et al., 1983; Dumontet et al., 1999; Sidhu and Toze, 2009; Rudolfs et al., 1950a; 1950b) are either outdated, focused on matrices other than fecal sludge (e.g., manure, compost), did not include all pathogen groups (viruses, bacteria, protozoa, and helminths), did not consider design, operational, and environmental factors, and/or were not done systematically.

Therefore, the purpose of this study was to use systematic literature review and meta-analysis to quantify the decay rates of pathogens in fecal sludge and to determine the extent to which pathogen decay is influenced by parameters such as pH, temperature, moisture content, and additives for desiccation, alkalinization, and disinfection. This research is novel because while there has been guidance from United Nations’ Sustainable Development Goal #6 to provide safely managed sanitation for all by 2030, there is no clear evidence for recommendations about how long fecal waste from onsite sanitation systems should be stored and under what conditions it should be stored before it can be safely emptied, handled, and discharged or reused. This research helps answer that question by quantifying pathogen decay patterns and decay rates in fecal sludge with respect to design, operational, and environmental factors.

The following questions were addressed in this review: (1) Which model, between the log-linear regression model and the non-linear JM2 model (Juneja et al., 2006; Mitchell and Akram, 2019), best represents the decay of viruses, bacteria, protozoa, and helminth eggs in fecal sludge? (2) How do decay rate coefficients and T_99_ values compare between viruses, bacteria, protozoa, and helminths? (3) To what extent do design, operational, and environmental factors such as pH, temperature, moisture content, the use of additives such as urea, lime, and ash, and the separation of urine from excreta influence pathogen inactivation rates in fecal sludge?

## 2 Materials and methods

### 2.1 Search strategy

PRISMA guidelines (Moher et al., 2016) were followed (Table S3) and a protocol was registered and published in PROSPERO (Musaazi et al., 2020) before the review began. Studies were identified from peer-reviewed literature using the following Boolean search string: [(sanitation OR latrines OR toilets OR septic tanks) AND (pathogens OR bacteria OR helminth OR protozoa OR indicator OR virus) AND (fecal sludge OR feces OR excreta OR compost) AND (persistence OR die-off OR survival OR decay OR inactivation OR removal OR treatment)]. Results were not restricted by date. The following databases were consulted in February 2020: PubMed, Web of Science and Scopus. An additional search was made on the bibliographies of studies identified in the first round.

### 2.2 Study selection and eligibility

Results from the search were imported into Mendeley, and duplicate entries were removed. Remaining articles were exported to a spreadsheet. For the primary review, titles and abstracts were screened independently by two co-authors using the inclusion criteria described in Musaazi et al. (2020). Briefly, studies needed to include: a) persistence or survival with a time component; b) individual quantitative measurements of human pathogens or fecal indicators at each time point; c) fecal sludge, feces, or human excreta. Abstracts that described studies of occurrence only, without a time and decay component, were excluded. Similarly, studies of microorganisms that are neither human pathogens nor fecal indicators were excluded. Studies of the decay of antibiotic or antimicrobial resistance genes were not included. Studies of persistence in non-target matrices (e.g., soils, fomites, non-human fecal or organic waste, sludge from centralized sewage systems, freshwater, marine waters) were also excluded.

For the secondary review, full texts were read by the lead author and another co-author. All studies that did not meet the aforementioned criteria were excluded, as well as studies where: 1) the physical and chemical nature of the fecal sludge was not well described, 2) experiments took place with exposure to natural or simulated sunlight; 3) primary data such as exposure times and corresponding log_10_ reduction could not be determined; 4) methods used for enumerating microorganisms were not well described.

### 2.3 Data extraction

The following data were extracted from eligible studies: author, publication year, study location, sanitation technology, name of microorganism or microbial group, and the method used to quantify microorganisms. For studies of viruses and bacteria, only data from experiments using culture-based methods were included. All experiments with protozoa (even those that did not assess viability) were included in the meta-analysis, since the assessment of viability is not part of standardized methods for protozoan pathogens. Pathogen concentrations and corresponding storage times were recorded, as well as information about fecal sludge conditions under which these changes in concentrations were observed (e.g., temperature, pH, moisture content). Unique IDs were created to differentiate each experiment. Data collection forms were not used. If the moisture content was not reported, it was calculated from the percent total solids (dry weight per total weight). Rarely, the pH (5.8% of experiments), temperature (2.5% of experiments), or moisture content (3.3% of experiments) of the fecal sludge was not reported. In these cases, values were imputed using the multivariate imputation by chained equations (mice) package in R (van Buuren 2021). Data presented graphically were digitized using WebPlotDigitizer v4.1 (Drevon et al., 2017, Rohatgi et al., 2019). Extracted data from all papers were compiled by a single author, and data from a randomly selected subset of studies (10%) were independently extracted by a second author, for quality control. Discrepancies found (<5%) were corrected. All data were compiled into a CSV file, which was uploaded to the Global Water Pathogen Project (GWPP) K2P Data Portal (https://data.waterpathogens.org/dataset/persistence-treatment).

Quality of reported values and risk of bias was assessed based on the level of detail provided for the laboratory methods. Papers were qualitatively assigned a score based on: 1) sufficient description of the methodology (or reference to a document with sufficient description) to repeat the experiment and understand what strain/group of microorganisms was measured (up to 1 point); 2) use of internal controls to determine percent recovery (up to 1 point); and 3) reporting the limit of detection (up to 1 point). Papers of all quality scores were used in the meta-analysis, but the quality scores were used to provide context about the limitations associated with some of the findings.

### 2.4 Model comparison

All data analysis was done using R (R Core Team, 2017), the code can be found in Supporting Information. The log-linear decay model (Chick 1908) and the JM2 model (Juneja et al., 2006) were compared for goodness of fit. The log-linear model was chosen for its simplicity and broad applicability to pathogen decay in multiple matrices. The JM2 model was chosen because it was previously reported as the best model for pathogen survival in sewage, sludge, biosolids, and manure (Mitchell and Akram, 2019). For the log-linear model, first order decay rate coefficients (*k*) were calculated as the slope of the natural log change in pathogen concentrations (ln(C_t_/C_0_)) with respect to time (in days), where C_t_ is the pathogen concentration at time t, and C_0_ is the concentration at time zero:

(1) $$\frac{C_{t}}{C_{o}}=e^{-kt}$$

The JM2 model is represented by the following equation, where *C*_t_ is the concentration at time *t, C*_0_ is the concentration at time zero, and *k*_1_ and *k*_2_ are coefficients (Juneja et al., 2006):

(2) $$\frac{C_{t}}{C_{o}}=\frac{1}{1+e^{k_{1}+k_{2}\ln\left(t\right)}}$$

Maximum likelihood estimation was used with unbounded optimization to find the best fit parameters (k_1_ and k_2_) based on data from each experiment. Portable Fortran programs for numerical computation (PORT) routines were used for function minimization (Fox et al., 1978), implemented via the *nlminb* function and the *bbmle* package in R (Bolker, 2020).

Data from experiments with fewer than four timepoints and from experiments where concentrations were reduced by less than 90% were not used for model selection. The former group was not used because it is not enough timepoints to properly fit the JM2 model. The latter group was not used because the variability and uncertainty associated with pathogen measurements was similar to the variation associated with decay, producing erratic estimates for model parameters. Many of these experiments were the control groups in studies that evaluated different treatment methods (e.g., McKinley et al., 2012; Ogunyoku et al., 2016; Pompeo et al., 2016). It was likely the authors’ intent to have low reduction in these control groups compared to the treatment group(s). Nevertheless, data from these experiments were still used to estimate the upper limits of T_99_ values and to evaluate the influence of design, operational, and environmental factors on log-linear decay rate coefficients, since omitting them from this analysis could have skewed the results. The Akaike information criterion (AIC) (Akaike, 1974) was used to assess the relative quality of both models for all experiments with at least four timepoints and a maximum reduction of at least 90%.

### 2.5 Estimation of T_99_ values

T_99_ values (time required for 2-log_10_ reduction) were estimated by algebraically rearranging Eqs. (1) and 2 to solve for the time that corresponded with a value of C_t_/C_0_ = 0.01. T_99_ is commonly used to describe pathogen persistence (Mitchell and Akram 2019), so it allows for comparison with other studies. Ideally, the T_99_ values should be interpolated from the models, however many experiments did not reach 99% reduction. Therefore, the decay models were used to extrapolate for some experiments, but only up to a distance equal to twice the maximum timepoint from the dataset. If extrapolation of T_99_ was not possible within these limits, then the T_99_ value was considered to be censored, and was reported to be greater than 2x the maximum timepoint in the dataset. For example, in one of the experiments by Nordin et al. (2009a), measurements of *Ascaris* eggs were taken at five timepoints, with a maximum time point of 22 days resulting in a maximum reduction of 0.86 log_10_ units. Extrapolation using the best fit model (JM2) produced a T_99_ estimate of 67.2 days. However, since the maximum timepoint was only 22 days, we reported the T_99_ value to be censored at >44 days.

Summary statistics for T_99_ were calculated using the *cenfit* command from the NADA package (Lee, 2020), which implements the nonparametric Kaplan-Meier (KM) method. This method is considered the standard approach for estimating summary statistics of censored data (Helsel, 2012). The censoring level was assumed to be equal to twice the maximum timepoint from the dataset. Since the dataset was right-censored, data values were flipped prior to implementing the KM method, as described by Helsel (2012). The Peto-Prentice version of the generalized Wilcoxon test (Helsel, 2012) was used to test for significant differences between T_99_ values from different microbial groups, using the *cendiff* command from the NADA package (Lee, 2020).

### 2.6 Influence of design, operational, and environmental factors

Pearson's correlation was used to understand the degree to which the decay rate coefficients moved in coordination with the design, operational, and environmental factors (i.e., pathogen group, pH, moisture content, temperature, use of urea, or use of desiccating/alkalinizing agents). Analysis of variance (ANOVA) and multiple linear regression (von Sperling et al. 2020) were used to assess the influence of design, operational, and environmental factors as independent variables on the log-linear decay rate. Prior to completing these analyses, the distributions of decay rate coefficients were checked, and log or Box-Cox transformations were applied to make distributions more symmetrical. The application of additives for desiccation, alkalinization, or disinfection were treated as categorical because the concentrations applied were not always reported. In studies where urea was added to fecal sludge, the concentration (when reported) was usually at least 0.5% on a wet mass basis.

## 3 Results and discussion

### 3.1 Search results

**Fig. 1:**
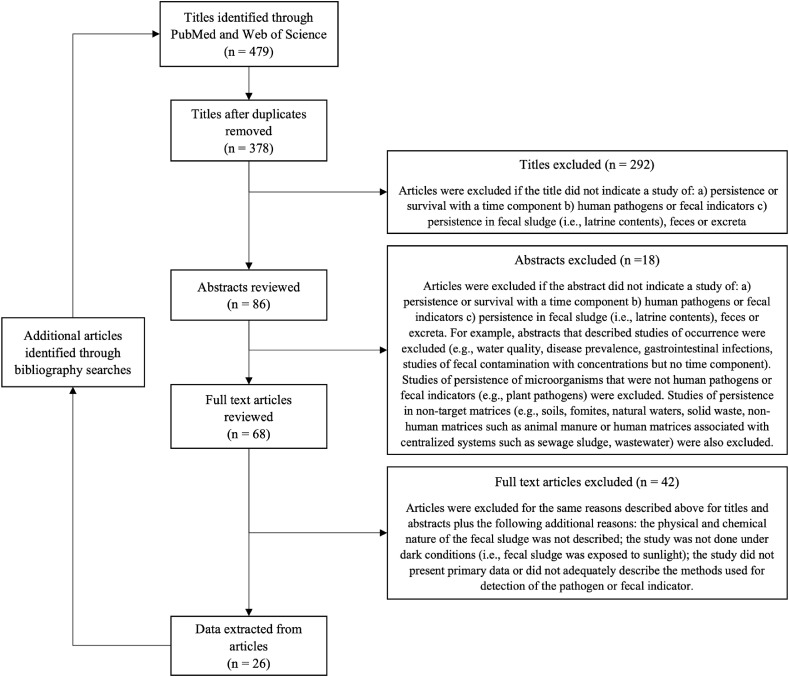
Flowchart depicting the number of articles identified in the search, the number excluded based on review of the title, abstract, and full text, and the number from which data were extracted.

Of the 479 articles identified in the search, 292, 18, and 42 articles were excluded based on the review of the title, abstract, and full text, respectively (Fig. 1). A full list of the 26 articles that met the inclusion criteria (Table S4), as well as articles that appeared to meet the criteria but were excluded after reviewing the full text (Table S5) can be found in Supporting Information. A total of 1382 data points were extracted from 243 experiments described in the 26 articles (Anderson et al., 2015; Berendes et al., 2015; Chien et al., 2001; Darimani et al., 2015; Decrey and Kohn, 2017; Endale et al., 2012; Fidjeland et al., 2013; 2016; Graham et al., 2003; Hashemi et al., 2019; Jensen et al., 2009; Magri et al., 2013; McKinley et al., 2012; Moe and Shirley, 1982; Nakamura and Taylor 1965; Niwagaba et al., 2009; Nordin et al., 2009a; 2009b; Odey et al., 2018; Ogunyoku et al., 2016; Pompeo et al., 2016; Robertson et al., 1992; Sossou et al., 2014; 2016b; Tønner-Klank et al., 2007; Yin et al., 2016). 1078 data points were extracted from 190 laboratory-based experiments and 304 data points were extracted from 53 field-based experiments. Of the 243 experiments, 32 were performed with viruses, 147 with bacteria, 8 with protozoa, and 56 with helminth eggs. In 23 of the 32 experiments with viruses, (72%) bacteriophages were used (MS2, PhiX174, T4, and *Salmonella* 28B phages); animal viruses (rotavirus and adenovirus) were used in the other nine experiments (28%). Experiments with bacteria included both indicators and pathogens (total coliforms, fecal coliforms, *Streptococcus* spp., *E. coli, Enterococcus* spp., *Salmonella* spp., *Shigella* spp., and *Clostridia* spp.). Qualifying experiments with protozoa included *Cryptosporidium, Giardia*, and *Entamoeba. Ascaris* eggs (*A. suum* or *A. lumbricoides*) were the only type of helminth in qualifying experiments.

### 3.2 Experimental design

Viruses were quantified as plaque forming units (PFUs) in 75% of the experiments and as fluorescent cell forming units (FCFUs) in 25% of the experiments. Bacteria were quantified as colony forming units (CFUs) in 92% of the experiments and most probable number (MPN) in 8% of the experiments. Half of the experiments of protozoa assessed viability using stains (e.g., propidium iodide), and the other half only counted total (oo)cysts using immunofluorescence microscopy. The viability of helminth eggs (*Ascaris*) was assessed in all experiments, with 89% based on morphology after incubation (e.g., US EPA (2003) or similar) and the other 11% based on the use of stains such as Safranin O (Sossou et al., 2014; 2016b).

In 144 of the 243 experiments (59%), fresh stool, feces, or the contents of dry/desiccation toilets (e.g., with urine diversion) were used. In the other 99 experiments (41%), the matrix included feces and fresh urine, and of those, 58 also included flushing water (e.g., sampling from pour flush toilets, cesspits, septic tanks; mixing feces and urine with water). The three most common locations for experiments were in Sweden (40 of 243 experiments), Burkina Faso (39 of 243 experiments), and China (21 of 243 experiments). Other experiments were performed in Bolivia, Brazil, Burkina Faso, China, Great Britain, Ethiopia, Haiti, Japan, Korea, Malawi, Mexico, Scotland, Sweden, Switzerland, Uganda, United States, and Vietnam.

About half (50.6%) of the experiments used fecal sludge with temperatures between 22 °C and 34 °C. In 16% of the experiments, the temperature was above 50 °C, and in 3% of experiments the temperature was below 10 °C. The interquartile ranges of pH and moisture content were 7.2 to 9.0 and 40% to 90%, respectively. The pH of the fecal sludge was below 6 in 8% of the experiments and above 9 in 24% of the experiments. The moisture content was below 10% in 7% of the experiments and above 90% in 25% of experiments. In 87 of 243 experiments, alkalinizing or desiccating agents were added to modify the pH or moisture content—lime and ash were used in 28 experiments each and organic materials (sugarcane husks, rice husks, shea nut shells, etc.) were added for co-composting in 23 experiments. Urea was applied in 57 experiments.

Of the 243 experiments, 96 (40%) originated from studies with the maximum quality score of 3.0, 47 (19%) originated from studies with quality scores of at least 2.0, 66 (27%) originated from studies with quality scores of at least 1.0, and the other 34 (14%) originated from studies with quality scores of less than 1.0. The most common reasons for low quality ratings were the lack of recovery controls during sample concentration and not reporting limits of detection. Except for protozoa, there were no major differences between quality ratings of papers with experiments using different microbial groups—63% of virus experiments, 53% of bacteria experiments, and 83% of helminth experiments received a score of 2 or higher. For the 8 experiments with protozoa, the scores were all between 1 and 2, mostly due to the lack of process recovery controls and the lack of reporting the method detection limits. For additional statistics about the experimental conditions and the quality control scores, see Tables S1 and S2.

### 3.3 Model selection

**Fig. 2:**
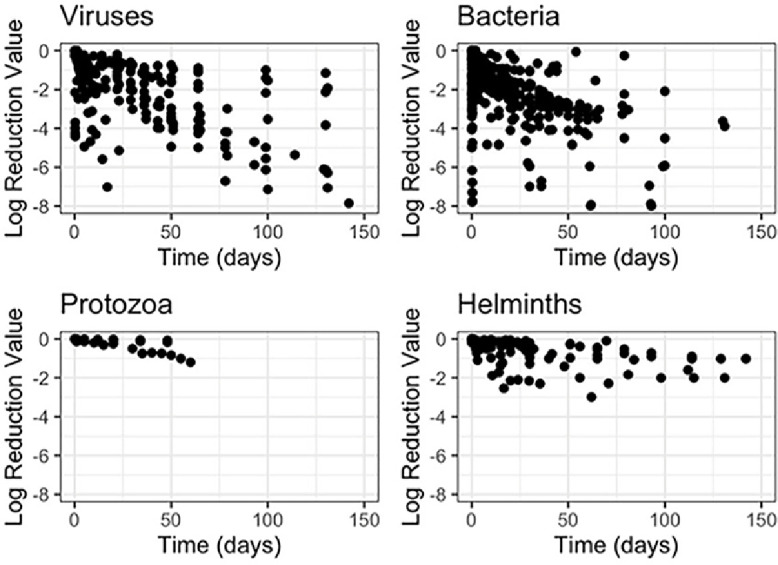
Microbial log_10_ reduction in fecal sludge with respect to time for viruses, bacteria, protozoa, and helminths (*Ascaris*).

Fig. 2 shows log_10_ reductions with respect to time for all experiments. Reductions were lower for *Ascaris* and protozoa than they were for viruses and bacteria, which is consistent with a previous review of pathogen decay in sewage, urine, manure, and surface waters (Mitchell and Akram, 2019). The reduction of bacteria was more variable than other microbial groups. In most experiments, bacteria were reduced by at least 2-log_10_ within 50 days, but the same reduction took longer for viruses, protozoa, and *Ascaris* eggs.

Most experiments (53%) had 4, 5, 6, or 7 timepoints, and 22% of the experiments had more than 7 timepoints. In 38 (16%) of the experiments, there were only three timepoints with concentrations above the LOD, which is too few to fit the JM2 model. Similarly, 23 (9%) of the experiments had only two timepoints with concentrations above the LOD. These experiments were not used for model selection, but they were used to estimate T_99_ values using the log-linear model. The justification was that these experiments had rapid decay rates (e.g., concentrations fell below the LOD after the first or second timepoint), and not including them would have skewed the results by preferentially eliminating experiments with conditions that promoted rapid pathogen decay. In 65 of the 243 experiments (27%), microbial reduction never reached 90%. These data were not used for model selection. In 11 experiments (5%), there were fewer than four timepoints and maximum reduction was less than 90%, meaning that 115 of 243 experiments (47%) either had too few timepoints or the reduction was too low to be used for model selection.

Of the experiments that qualified for model selection, the JM2 model had lower AIC values than the log-linear model for 81 experiments (63%), indicating that JM2 generally provided a better fit. The R^2^ values for the log-linear model were generally greater than 0.5 (Fig. S3), but data from experiments that displayed characteristic tailing effects (e.g., Niwagaba et al., 2009; Hashemi et al., 2019) were better modeled using the JM2 equation (Fig. S1; Experiments 292 – 299, 323 – 325). Some experiments with *Ascaris* eggs (e.g., Fidjeland et al. 2016; McKinley et al., 2012) displayed a characteristic shouldering effect, which was modeled well using the JM2 equation (Fig. S1; Experiments 192 – 195, 262 – 263). Some experiments with bacteria (Magri et al., 2013; Nakamura and Taylor, 1965; Niwagaba et al., 2009; Nordin et al., 2009b; Tønner-Klank et al., 2007) showed erratic patterns of decay and apparent regrowth, which could have resulted from true regrowth, random sampling, or large variabilities associated with recovery, isolation, quantification, and enumeration of microorganisms in fecal sludge. For slightly more than half of the 31 qualifying experiments with viruses, the JM2 model had lower AIC values (log-linear had lower AIC in 15 experiments; JM2 had lower AIC in 16 experiments). The JM2 model was also the best model for 48 of 76 qualifying experiments with bacteria, two of three qualifying experiments with protozoa, and for 15 of 18 qualifying experiments with *Ascaris*.

### 3.4 Decay rate coefficients

The estimated *k* values (and standard errors) and *k*_1_ and *k*_2_ values (and sigma values) for all experiments are presented in Table S6. Table 1 shows summary statistics of the calculated decay rate coefficients for the log-linear and JM2 models (also see Fig. S5). Protozoan (oo)cysts had the lowest log-linear decay rates, with a median *k*-value of 0.0095 days^−1^. Decay rates for *Ascaris* were only slightly higher, with a median *k*-value of 0.0042 days^−1^. The protozoa decay rates are based on an extremely limited set of experiments originating from only three papers (Graham et al., 2003; Robertson et al. 1992, Sossou et al., 2014) where viability was only assessed in 3 of 4 experiments using dye stains. These experiments were also done with lower moisture levels and higher temperatures than most experiments done with *Ascaris* eggs (Table S1). The median decay rates for viruses and bacteria were 0.19 days^−1^ and 0.93 days^−1^, respectively. This is consistent with previous publications that have shown that protozoan cysts and helminth eggs persist longer in the environment than viruses and bacteria, since they possess durable cell walls that enhance survival (Peng et al., 2008; Ben Ayed and Sabbahi, 2019). It is also consistent with the findings of Mitchell and Akram (2019), who reported higher T_90_ and T_99_ values for viruses than for bacteria in sewage, urine, manure, and surface waters. Uncertainty in estimated decay rate coefficients was also larger for bacteria and viruses than it was for *Ascaris* and protozoa, as evidenced by average standard errors for decay rate coefficients of 0.92, 1.04, 0.13, and 0.33 for viruses, bacteria, protozoa, and *Ascaris*, respectively.

**Table 1:** Calculated log-linear and JM2 model decay rate coefficients in fecal sludge for each microbial group.

**Microbial group**	**Number of experiments used for log-linear model (JM2 model)**^a^	**Calculated pseudo-first order decay rate coefficient, *k* (days^−1^)**			**Calculated JM2 model coefficients**					
					**Coefficient *k*_1_**			**Coefficient *k*_2_**		
		**Median**	**5%**	**95%**	**Median**	**5%**	**95%**	**Median**	**5%**	**95%**
Viruses	32 (31)	0.19	0.025	16.8	−7.2	−31.1	17.7	4.9	1.5	17.9
Bacteria	147 (120)	0.93	0.012	243	0.88	−78.3	216	3.6	0.45	216
Protozoa	8 (4)	0.0095	0.0016	0.035	−13.4	−13.6	−6.5	3.1	2.1	3.31
Helminths^b^	56 (23)	0.0042	0.000008	0.52	−8.7	−58.1	−1.8	3.8	1.4	17.6

a Log-linear decay rate coefficients were calculated from all experiments, including experiments with 2 time points; JM2 coefficients were only calculated from experiments with at least four time points.

b All qualifying experiments were done with *Ascaris* eggs.

Uncertainty in JM2 model parameters was larger for bacteria and viruses than it was for *Ascaris* and protozoa. The average values of σ (see Supplemental Information) were 1.12, 1.63, 0.12, and 0.21 for viruses, bacteria, protozoa, and *Ascaris*, respectively. The *k*_1_ value controls the amount of shouldering and the general slope of the curve at the beginning of decay, with more negative values consistent with flat curves with longer shoulders. Median *k*_1_ values were generally found to be negative, except for bacteria. *Ascaris* and protozoa had the most negative *k*_1_ values, indicating that shouldering may be more prevalent for the decay of these pathogens in fecal sludge, compared to viruses and bacteria. The *k*_2_ value controls the tail and the flatness toward the end of the curve, with lower values consistent with curves that flatten out earlier and higher values consistent with curves that flatten out later. The median *k*_2_ values for bacteria, protozoa, and *Ascaris* ranged from 3.1 to 3.8, and viruses had the highest median *k*_2_ value of 4.9. The higher *k*_2_ and less negative *k*_1_ value for viruses reflects quicker decay with less tailing compared with *Ascaris* and protozoa.

### 3.5 T_99_ values

There were 74 experiments where T_99_ values were censored (i.e., microbial reduction never reached 99%, and T_99_ could not be estimated using the best fit model without extrapolating more than twice the maximum timepoint, as described in the Methods section). Table 2 shows summary statistics of estimated T_99_ values for both models independently, and using whichever model provided the best fit. There was a significant difference between the T_99_ values for the different microbial groups based on the generalized Wilcoxon test (χ^2^ = 79.8 on 3 degrees of freedom when the best fit models were used, *p* < 0.0001; see Fig. S10). Predicted T_99_ values for viruses were similar for both models, with median values of ∼1 month, with the upper 95th percentile equal to 140 days when the best fit models were used. There were divergences in the predicted T_99_ values of bacteria and *Ascaris* eggs, depending on which model was used. For instance, median T_99_ values for bacteria ranged from slightly less than one week to slightly more than two weeks for the log-linear and JM2 models, respectively, with a median value of just under 5 days when the best fit models were used. For *Ascaris*, median predicted T_99_ values ranged from 260 days for the log-linear model to 429 days when the best fit models were used. Due to higher censoring when the JM2 model was used, the median T_99_ value could only be determined to be greater than 67 days. The divergence between summary statistics computed based on the different models can be explained by tailing patterns that are represented with the JM2 model, but not the log-linear model.

**Table 2:** Calculated T_99_ values in fecal sludge for each microbial group.

**Microbial group**	**T_99_ values (days) based on the log-linear model**				**T_99_ values (days) based on the JM2 model**				**T_99_ values (days) based on the best fit model**			
	**N**	**Median**	**5%**	**95%**	**N**	**Median**	**5%**	**95%**	**N**	**Median**	**5%**	**95%**
Viruses	32	30	b	140	32	29	0.40	216	32	29	b	140
Bacteria	147	5.8	0.0031	141	113	16	0.13	148	147	4.8	0.0035	148
Protozoa	8	>321	b	>360	8	>341	221	>360	8	>341	b	>360
Helminths^a^	56	260	15	>658	53	>67	16	>658	56	429	9.7	>658

a All qualifying experiments were done with *Ascaris* eggs.

b Quantile could not be estimated using the K-M method due to excessive censoring.

Of the eight qualifying experiments with protozoa, none achieved more than 99% reduction, so all T_99_ values are based on model extrapolations. Despite this limitation, the median T_99_ value was still estimated to be greater than 341 days. Graham et al. (2003) reported a 83% and a 67% decrease in the number of *Giardia* cysts in fecal sludge from biodegrading and dehydrating toilets, respectively, after a storage period of 6 months (however, the authors did not measure viability). The estimated T_99_ value for *Cryptosporidium* was 221 days or more, and the estimated T_99_ value for *Entamoeba* was 109 days or more (depending on which models were used). These T_99_ values are much longer than 86 days and 70 days, which is what has been previously reported for *Cryptosporidium* in swine waste (Jenkins et al., 2013) and groundwater (Sidhu and Toze, 2012), respectively. More research is needed to better understand the persistence of protozoan pathogens in fecal sludge matrices, as the findings presented here are based on limited data.

### 3.6 Influence of pH, temperature, moisture, urine diversion, and additives

The distribution of estimated log-linear decay rate coefficients (*k*) was highly skewed. Natural logarithm transformations made the distribution more symmetrical (Fig. S4). Likewise, the distribution of JM2 model coefficient *k*_2_ was skewed and a Box-Cox transformation ($\sqrt{\ln(k_{2})}$) made the distribution more symmetrical. The distribution of *k*_1_ values was already relatively symmetrical but had very long tails.

#### 3.6.1 pH

The pH of fecal sludge had no significant correlation with ln(*k*) (Pearson's *r* = −0.10, *p* = 0.109), *k*_1_ (Pearson's *r* = −0.14, *p* = 0.067), or $\sqrt{\ln(k_{2})}$ (Pearson's *r* = 0.023, *p* = 0.76) (Fig. S7a), which is consistent with previous studies of pathogen persistence in animal manure (Himathongkham and Riemann, 1999). The presence of free ammonia at high pH levels may drive inactivation more than pH alone (Kohn et al., 2017), unless very high pH levels are reached (e.g., >12). Ghiglietti et al. (1997, 1995) reported that *Ascaris* eggs treated with ammonia at pH 11.9 and a temperature of 30 °C were inactivated, and that thermal treatment at 40 °C impeded the eggs’ development even in the absence of ammonia. Mehl et al. (2011) suggested that a pH of 9 or above will inactivate pathogens (including helminth eggs) after six months, if temperatures are above 40 °C.

#### 3.6.2 Temperature

Temperature correlated significantly with ln(*k*) (Pearson's *r* = 0.52, *p* < 0.0001), *k*_1_ (Pearson's *r* = 0.55, *p* < 0.001), and $\sqrt{\ln(k_{2})}$ (Pearson's *r* = 0.42, *p* < 0.001). This is consistent with a review by Espinosa et al. (2020), which indicated that temperatures above 50 °C yielded reductions of 3-log_10_ or greater within a few hours for many pathogens and within a month for the most resistant pathogens. However, the correlation was strongly influenced by 35 experiments at extreme temperatures (above 50 °C or below freezing), and fecal sludge in onsite toilets rarely reaches these temperatures. Data from experiments performed at temperatures above 50 °C or below 0 °C were removed for subsequent meta-analysis. After removing these data, temperature did not correlate significantly with ln(*k*) (Pearson's *r* = 0.10, *p* = 0.15) or $\sqrt{\ln(k_{2})}$ (Pearson's *r* = −0.13, *p* = 0.1154), but it did correlate significantly with *k*_1_ (Pearson's *r* = 0.20, *p* = 0.0134) (Fig. S7b). This indicates that extreme temperatures can influence pathogen decay in fecal sludge, but for systems operating at ambient temperatures, the influence may be minimal. The limited impact of temperature within the ambient range could also be attributed to the heterogeneous distribution of temperature in fecal sludge (Preneta et al., 2013; Tønner-Klank et al., 2007), which can be addressed by mixing (e.g., turning compost piles). In summary, temperatures needed to achieve rapid pathogen decay (i.e., above 50 °C) were not achieved in most of the experiments and might be difficult to obtain in a real-world setting, unless designs are modified, such as toilets with solar-heated chambers (Cruz Espinosa et al. 2012; Sossou et al., 2016a; Koottatep et al., 2020).

#### 3.6.3 Moisture

Interestingly, higher moisture correlated significantly with higher ln(*k*) values (Pearson's *r* = 0.28, *p* < 0.001), higher *k*_1_ values (Pearson's *r* = 0.20, *p* = 0.0059), and higher $\sqrt{\ln(k_{2})}$ values (Pearson's *r* = 0.17, *p* = 0.0195). This was counterintuitive, since desiccation should increase pathogen inactivation (King and Monis, 2007; Himathongkham and Riemann, 1999). In general, *Ascaris* eggs were subjected to higher moisture conditions than other pathogen groups in the experiments, and experiments with *Ascaris* eggs made up nearly one-fourth of the data set, which could be one explanation for the findings. Lower moisture should cause more rapid inactivation, but there is no consensus in the literature about how dry fecal sludge must be to experience this effect—it likely differs for different pathogens. *Ascaris* and *Entamoeba* persisted in pit latrines after 7 months storage at a moisture content of 29% (Gibson, 2014). Mehl et al. (2011) recommended less than 25% moisture for pathogen inactivation in pit latrines. However, others have noted that *Ascaris* eggs possess a multi-layered wall composed of chitin and lipids, which makes them tolerant to desiccation, thus moisture levels below 5% may be needed for their inactivation (Nordin, 2010). In 199 of the 243 experiments from this review, fecal sludge moisture levels were above 25%. Except for the experiments done by Endale et al. (2012), where the moisture content got as low as 3% after a month of storage, the lowest moisture in any other experiment was 7 – 8% (Magri et al., 2013), which may not be low enough to induce rapid inactivation. As such, observed decay rates from this review might be confounded by factors other than moisture. More research is needed to better understand the role of moisture on pathogen inactivation in fecal sludge.

#### 3.6.4 Sanitation technology (fecal sludge matrix)

**Fig. 3:**
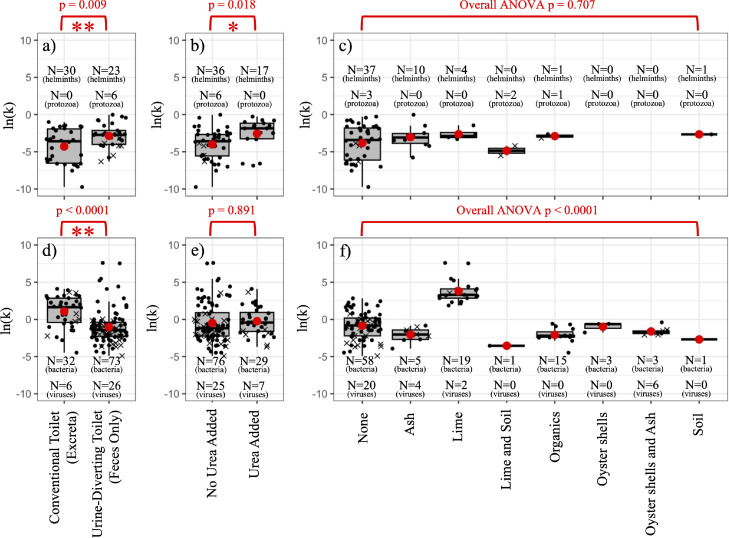
Natural log transformations of the pseudo first-order decay rate coefficients obtained for experiments with protozoa and *Ascaris* (panels a–c) or with bacteria and viruses (panels d–f).*Ascaris* and bacteria are shown with black circles; protozoa and viruses are shown with the symbol × . Panels (a) and (d) show results from matrices common to urine diversion toilets (feces only) vs. matrices common to conventional toilets (excreta, **i.e.**, urine mixed with feces); panels (b) and (c) show results when urea is used as a disinfectant; and panels (c) and (f) show the use of different additives for the desiccation or alkalinization of fecal sludge. For panel (f): the Tukey *post hoc* pairwise comparison test showed that lime was significantly greater than all other groups, and that organics was significantly lower than none; all other pairwise comparisons were not significantly different at the 0.05 level.

Fig. 3 shows log-transformed *k*-values (for the log-linear model) from experiments performed with different fecal sludge matrices (conventional vs. urine-diverting toilets), the use of urea for disinfection, and the use of additives for desiccation or alkalinization (e.g., ash, lime, soil, etc.). Fig. S6 shows similar box plots for *k*_1_ and *k*_2_ values obtained from the JM2 model. For *Ascaris* eggs and protozoan pathogens, ln(*k*) values from experiments with matrices consistent with fecal sludge from urine diversion toilets (feces only) were significantly greater (*p* = 0.009) than the ln(*k*) values from experiments with fecal sludge consistent with conventional pit toilets (excreta, i.e. feces and urine). For bacteria and viruses, the opposite was true (*p* < 0.0001). This suggests that the type of toilet may influence decay rates of different pathogen groups in different ways.

#### 3.6.5 Urea

On average, for experiments where urea (or stored urine) was added as a source of ammonia for disinfection, decay rates of *Ascaris* eggs and protozoan pathogens were significantly higher (*p* = 0.018) than they were when urea was not added. This effect was more pronounced in fecal sludge for urine diverting dry toilets (UDDTs) than it was in conventional toilets where fresh urine was mixed with fecal matter, perhaps due to pH differences. Fresh urine has a pH between 6 and 7, while the pH of commercial urea and stored urine is often as high as 9 (Hoglund, 2001). Ammonia produced from the hydrolysis of urea in stored urine acts as a disinfectant since it is in the NH_3_ form instead of the protonated form, which happens above pH 9 (Kohn et al., 2017). NH_3_ can diffuse through microbial cell membranes, raising the internal pH and reducing viability (Nelson and Pecson, 2005) through protein denaturation and the cleavage of nucleic acids (Nordin, 2010). In urine, the viability of protozoa is affected by natural ammonia (Hoglund, 2001), but the effects of ammonia sanitization may be minimal in pit latrines and other onsite toilets because of unfavorable pH conditions and the lack of airtight storage which permits ammonia loss to the atmosphere (Fidjeland et al., 2015). In experiments where urea or stored urine was added to fecal sludge (Anderson et al., 2015; Fidjeland et al., 2016; Nordin et al., 2009a; 2009b; Magri et al., 2013; McKinley et al., 2012; Ogunyoku et al., 2016; Sossou et al., 2016b), concentrations of non-protonated NH_3_ ranged from 14 mg/L NH_3—_N to above 4000 mg/L NH_3—_N, but were above 100 mg/L NH_3—_N in most experiments (70%). The concentration of non-protonated ammonia (NH_3—_N) correlated strongly with the amount of urea applied and the pH of the fecal sludge (Fig. S2). Unlike *Ascaris* and protozoa, there was no significant difference for viruses and bacteria between experiments done with vs. without urea (*p* = 0.891).

#### 3.6.6 Alkalinizing and desiccating additives

Lime was the only additive that caused significantly higher decay rates compared to the control (no additives) and compared to all other additives (*p* < 0.0001). However, this was only true for experiments with viruses and bacteria. Pairwise comparisons revealed that the application of organic materials resulted in significantly lower decay rates of viruses and bacteria compared to the control group. For *Ascaris* eggs and protozoan pathogens, there were no significant differences between the different additive groups (*p* = 0.707). However, significant effects of additives have been reported in individual studies, but there may have been confounding factors. Graham et al. (2003) compared *Giardia* and *Cryptosporidium* in the contents of UDDTs with lime addition vs. non-flush vault toilets without urine diversion, finding more rapid reduction in the UDDT with lime—however, the authors did not assess viability. Endale et al. (2012) studied the decay of *Ascaris* eggs in feces (no urine) after adding lime, soil, ash, or nothing (experiments 128 – 131; Fig. S1), reporting that experiments with lime and ash produced similar decay rates that were only slightly faster than the control (no additives). McKinley et al. (2012) found that the application of ash to excreta without fresh urine (experiment 193) provided more rapid inactivation of *Ascaris* eggs relative to the control (experiment 191), but the use of ash for excreta mixed with fresh urine (experiment 194) had much more rapid decay, and decay was even faster when excreta was mixed with stored urine (experiment 195). Ogunyoku et al. (2016) reported more rapid inactivation of *Ascaris* eggs when lime and urea were added, compared to a lower decay rate in feces with lime but without urea (experiments 179 – 181); the latter group had similar decay compared to the control (feces plus urea but without lime). Nordin et al. (2009a) reported a reduction of ∼1 log_10_ after 35 days of storage with ash (experiment 187), with much faster reduction when urea was also added at a concentration of 1% by weight (experiment 189). This indicates that the effect on *Ascaris* eggs may be derived more from the disinfection properties of NH_3_ rather than the addition of just ash or lime.

The addition of 20% quicklime (dry mass basis) can raise the pH of sludge from pH 6.5 to pH 12 (North et al., 2008). As mentioned above, pH alone did not correlate with decay rates and the use of ash did not have as much of an impact as lime on the decay of bacteria and viruses. Endale et al. (2012) reported that ash treatment of fecal sludge in an eco-sanitation system took one month to achieve the same reduction of fecal coliforms as lime treatment achieved in one day. Like lime, ash can also increase the pH of fecal sludge, but larger doses are required. For example, Monney and Awuah (2016) found that the addition of 180 g of wood ash per liter of sludge produced pH levels of ∼11, but only 21.3 g of lime per liter of sludge increased the pH to above 12. Endale et al. (2012) and McKinley et al. (2012) raised the pH of fecal sludge to 9 with ash, but when they used lime, the pH reached 11 or 12. In summary, ash does not appear to have the same level of disinfection efficacy as lime for the treatment of fecal sludge. Also, there is not enough evidence to suggest that the decay of *Ascaris* eggs in fecal sludge is significantly increased by only adding lime (e.g., without also adding urea).

### 3.7 Multiple linear regression

Table 3 shows the coefficients, standard errors, and significance levels for the regression that predicts ln(*k*) using the independent variables: microbial group; temperature; moisture; pH; addition of urea or stored urine; use of UDDTs; and use of additives for desiccation or alkalinization. The intercept includes bacterial decay in fecal sludge with a pH of 8, a temperature of 20 °C, a moisture content of 60%, without urine diversion, and with no urea or other additives added, which are typical conditions for a pit latrine (Bakare et al., 2012; Nabateesa et al., 2017; Zuma et al., 2015). The model was significant with a residual standard error of 1.669, an F-statistic of 23.52 on 15 and 177 degrees of freedom (*p* < 0.0001). Standardized residuals were randomly distributed, with limited deviations from normality (Fig. S8). The R^2^ value was 0.6659 (adjusted R^2^ = 0.6376), so the model accounted for two-thirds of the variability in decay rates.

**Table 3:** Summary table of the regression model coefficients and corresponding p-values.

**Variable**	**Estimate**	**Standard Error**	**p-value**	**Significance**
Intercept (Bacteria)	−1.974	1.272	< 0.001	***
Virus	−0.902	0.371	0.016	*
Protozoa	−3.585	0.812	< 0.001	***
Helminth (*Ascaris*)	−3.120	0.321	< 0.001	***
pH (pH – 8)	0.350	0.128	0.007	**
Temperature (*T* – 20)	0.061	0.014	< 0.001	***
Moisture (*M* – 60%)	0.021	0.005	< 0.001	***
UDDT (feces only)	1.232	0.376	0.001	**
Urea addition	0.469	0.352	0.185	
Additive (ash)	−0.943	0.534	0.079	.
Additive (lime)	3.484	0.510	< 0.001	***
Additive (lime and soil)	−1.726	1.191	0.149	
Additive (organics)	−1.599	0.507	0.002	**
Additive (oyster shells)	−0.554	1.018	0.587	
Additive (oyster shells and ash)	−0.279	0.661	0.673	
Additive (soil)	0.283	1.226	0.818	

The coefficients for pH and temperature were positive and significant, indicating that higher pH and higher temperatures caused more rapid pathogen decay. The coefficient for UDDT was positive and significant, indicating the benefits of urine diversion on decay rates. The coefficient for urea addition was positive but not significant. This suggests that the use of urea alone may not significantly improve pathogen decay in onsite toilets, perhaps unless pH is also increased.

All additives except for lime and organics had non-significant coefficients, implying a limited effect on pathogen decay. Lime had a significant positive coefficient, implying that it increased decay rates. Organic materials for co-composting, such as sugarcane husks (Berendes et al., 2015), rice husks (Hashemi et al., 2019), or shea nut shells (Sossou et al., 2014), had a significant negative coefficient (*p* = 0.003), indicating a potential protective effect on pathogen survival. There have been mixed results about the impact of composting processes on pathogen reduction. For instance, Bustamante et al. (2008) reported that bacterial indicators and pathogens (e.g., *E. coli, Salmonella*) were not completely eliminated during the co-composting of winery and distillery wastes, and that the regrowth of bacterial pathogens was influenced by humidity, nutrient availability, and the presence of competitive microbiota. Manga et al. (2016) found that the use of chicken feathers for co-composting fecal sludge instead of organic market waste significantly impacted temperatures, which sped up the decay of helminth eggs.

The estimated intercept was −1.974, which equates to a *k* value of 0.139 days^−1^. This produces a T_99_ value of 33 days, implying that on average, approximately one month is required for a 2-log_10_ reduction of bacterial pathogens under the conditions typical of a pit latrine (pH 8, 20 °C, 60% moisture, no additives). The regression coefficients for viruses, protozoa, and *Ascaris* eggs were negative and significant, implying that these pathogen groups decay at slower rates relative to bacteria. For viruses, under the conditions typical of a pit latrine, the estimated *k* value of 0.056 days^−1^ produced a T_99_ of 82 days. For protozoan (oo)cysts and *Ascaris* eggs, the estimated *k* values under the same conditions (typical of a pit latrine) lead to predicted T_99_ values of more than a year. The regression also indicates that pathogens in fecal sludge at 20 °C survive 1.8 times longer on average than they do at 30 °C, which supports a recommendation that storage times should be twice as long in colder climates.

### 3.8 Policy implications

The findings from this study have broader policy implications for more than 3 billion people worldwide who use onsite sanitation systems (WHO/UNICEF 2020). Recommendations for the storage and treatment of fecal sludge have been made, but with limited supporting evidence. The WHO (2006) recommended storage times of 6 months with pH of 9 or more, temperature above 35 °C, and moisture below 25% (p. 69). Without alkaline treatment, they recommended storage of at least 1 year for temperatures of 20 – 35 °C, and 1.5 – 2 years for temperatures of 2 – 20 °C, stating that higher temperatures provide “more or less complete inactivation of *Ascaris* eggs … within 1 year” and “substantial to total inactivation of viruses, bacteria, and protozoa,” and that at the lower temperatures, viruses and parasitic protozoa would be reduced “below risk levels,” but that “some soil-borne ova may persist in low numbers” after 1.5 – 2 years (WHO, 2006). These recommendations were based on the works of Austin (2001), Carlander and Westrell (1999), Chien et al. (2001), Lan et al. (2001), Moe and Izurieta (2004), Peasey (2000), Phi et al. (2004), Strauss (1985), and Wang (1999), which are all either technical reports, conference proceedings, or conference abstracts. Except for Chien et al. (2001), all other cited works were either not accessible or did not meet the inclusion criteria for this systematic review and meta-analysis.

Stenström et al. (2011) made slightly different recommendations for the storage of fecal sludge from UDDTs when wood ash or lime was added, claiming that 6 – 12 months of storage would provide reductions “of up to 4 log units for viruses; 6 logs for bacteria; and a total reduction of viable protozoa and helminths.” Tilley et al. (2014) and the WHO (2018) repeated these recommendations for alkaline treatment of fecal sludge from UDDTs, suggesting a “minimum storage time of 6 months if ash or lime are used.” More recently, the WHO (2022) stated that more than 2-log_10_ reduction of pathogens (except for *Ascaris* eggs) is achieved after the fecal sludge from dry toilets with twin pits (fossa alterna) is stored for “at least 2 years”. For flush toilets with alternating pits, they suggested a similar reduction would be achieved after “at least 1 year storage at >20 °C, or storage of at least 6 months if pH is adjusted to >9 (e.g. with lime or ash)” (WHO, 2022). For regions where helminth infections are prevalent, they recommended “1.5–2 years of storage at 2–20 °C” (WHO, 2022), but did not cite any other new sources besides WHO (2006), Stenström et al. (2011), and Tilley et al. (2014).

Results from our meta-analysis provide a more expansive evidence base for recommendations about fecal sludge storage times. Our findings indicate that the use of lime and ash alone may not effectively inactivate *Ascaris* eggs unless urea is also added. Without urea, storage times for alkalinized fecal sludge from UDDTs should be longer than 6 months, due to risks presented not only from *Ascaris* eggs, but potentially also from protozoan pathogens (although more research is needed on protozoan pathogen viability under these conditions). Also, the storage of non-alkalinized fecal sludge for 1 year at temperatures of 20 – 35 °C does not provide “more or less complete inactivation” of *Ascaris* eggs, as originally suggested by WHO (2006). For all experiments with *Ascaris* eggs from this meta-analysis, where lime, ash, or urea were not added to the fecal sludge, and where the pH was less than 9, the maximum reductions were all less than or equal to 1.5-log_10_.

### 3.9 Limitations

There are several limitations to the findings from this study. First, only English-language publications in three databases (PubMed, Web of Science, and Scopus) were consulted. Second, the data used originated from articles that used different methods and potentially different levels of quality control. We qualitatively assigned a score to each paper based on the description of the methods used and the use of process controls, but all data were still used in the meta-analysis. Nevertheless, the potential bias created by experiments using different quality control methods was likely minimal, except for the experiments done with protozoa, since none of these experiments used process recovery controls and viability was not assessed in some of the experiments. A third limitation is that the data for helminth eggs was entirely based on experiments done with *Ascaris* and may not be representative of other helminths. Also, there were only eight experiments from three qualifying publications for protozoan pathogens. Only four of these experiments qualified for the calculation of decay rates, and in one of them (Graham et al., 2003), oocyst viability was not assessed, which could have led to underestimated decay rates and overestimated T_99_ values. For the regression, there were some scenarios for which very few experiments were performed. For example, there were 25 experiments where lime was added to fecal sludge—19 of them measured concentrations of bacteria, but only two measured viruses. There were no experiments with viruses or protozoa where urea was added to the fecal sludge and the pH was greater than 9—all data produced under those experimental conditions were for *Ascaris, Salmonella*, or fecal indicator bacteria. Another limitation of the findings from the regression analysis is that with all the factors involved in the model, the coefficients may not always reflect the true impact of the factor on pathogen decay. Dropping some factors could change the magnitude of coefficients for other factors. Another limitation is that most experiments were lab-based. A comparison of the results from field-based and lab-based studies for *Ascaris* eggs found slower decay and higher T_99_ values in the field-based studies, which may reflect differences between design, user habits, or other potential socioeconomic differences in field systems compared to controlled laboratory studies. More field-based research is needed to confirm trends that have been reported in laboratory settings.

## 4 Conclusions

We used systematic review and meta-analysis to draw insights about pathogen survival in fecal sludge from onsite toilets with respect to design, operational, and environmental factors. The JM2 model provided a better fit to experimental data than the log-linear Chick model for all pathogen groups. The rate of pathogen decay in fecal sludge was significantly different for different pathogen types. Bacteria died off the fastest, followed by viruses, then protozoa and helminths. The overall median T_99_ values were 4.8 days, 29 days, >341 days, and 429 days for bacteria, viruses, protozoan (oo)cysts, and *Ascaris* eggs, respectively, using the best fit models.

Microbial group, pH, temperature, moisture, toilet design (e.g., urine diversion), and the use of additives explained two-thirds of the variability in decay rate coefficients. As expected, higher pH values, higher temperatures, and the application of lime all significantly predicted greater decay rates, however lime was more effective for bacteria and viruses than it was for *Ascaris* eggs and protozoan pathogens. Fecal sludge stored for six months without treatment adequately controls hazards from viruses and bacteria, but much longer storage times or alkaline treatment with urea and low moisture or heat is needed to control hazards from protozoan (oo)cysts and helminth eggs.

## Declaration of Competing Interest

The authors declare that they have no known competing financial interests or personal relationships that could have appeared to influence the work reported in this paper.

## Appendix Supplementary materials

Image, application 1
