# Supplementary material for: A systematic review and meta-analysis of pathogen reduction in onsite sanitation systems
Source: Water Res X. 2023 Feb 21;18:100171. doi: 10.1016/j.wroa.2023.100171 (PMC10214292; doi:10.1016/j.wroa.2023.100171)
Supplement: Supplementary file 1 [file mmc1.docx]

**A systematic review and meta-analysis of pathogen reduction in onsite sanitation systems**

**Supporting Information**

*Isaac G. Musaazi^1†^, Shane McLoughlin^2^, Heather M. Murphy^2‡^, Joan B. Rose^3^, Nynke Hofstra^4^, Innocent K. Tumwebaze^2§^, Matthew E. Verbyla^1*^*

^1^ Department of Civil, Construction, and Environmental Engineering, San Diego State University, San Diego, California 92182, United States

^2^ Department of Epidemiology and Biostatistics, College of Public Health, Temple University, Philadelphia, PA, United States

^3^ Department of Fisheries and Wildlife, Michigan State University, East Lansing, MI, United States

^4^ Water Systems and Global Change Group, Wageningen University, The Netherlands

*^†^* Current affiliation: Department of Civil and Environmental Engineering, Howard University, Washington, D.C., United States

*^‡^* Current affiliation: Department of Pathobiology, Ontario Veterinary College, University of Guelph, Ontario, Canada

*^§^* Current affiliation: School of Architecture, Building and Civil Engineering, Loughborough University, England, United Kingdom

^*^ Corresponding author: mverbyla@sdsu.edu (M. E. Verbyla)


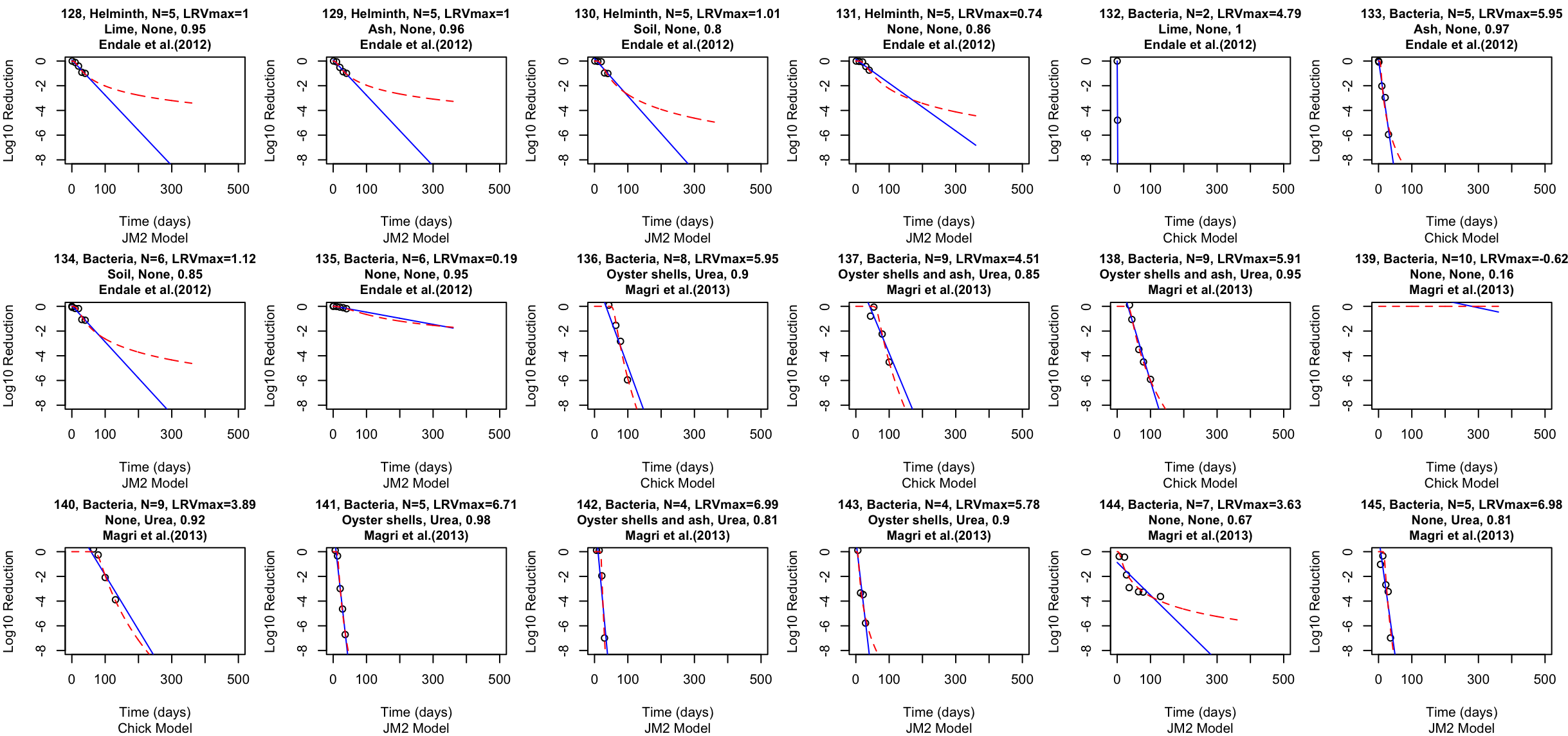

**Figure S1. Decay curves for experimental studies included in the meta-analysis to predict the decay rate coefficients.**

**
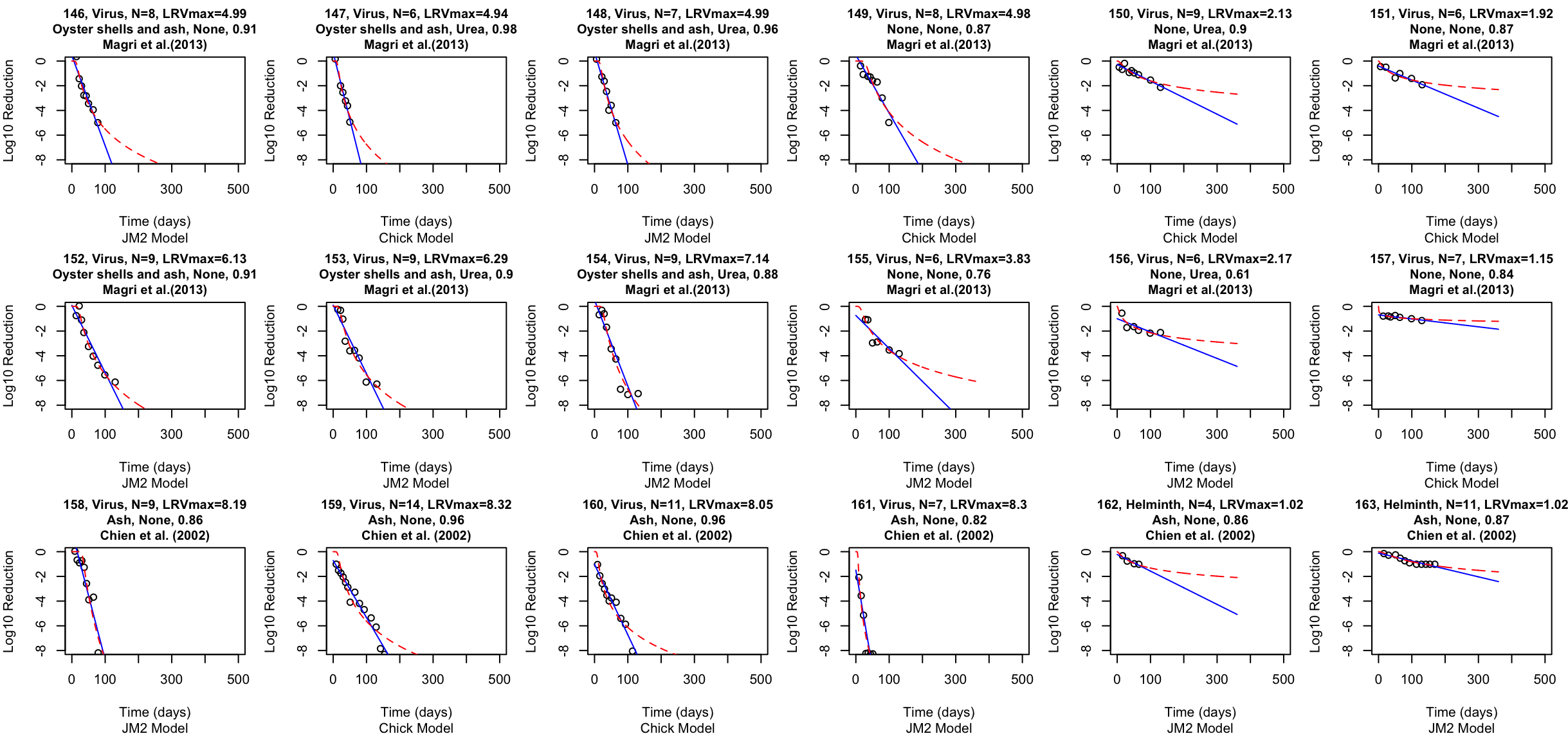
**

**Figure S1. Decay curves for experimental studies included in the meta-analysis to predict the decay rate coefficients (continued).**


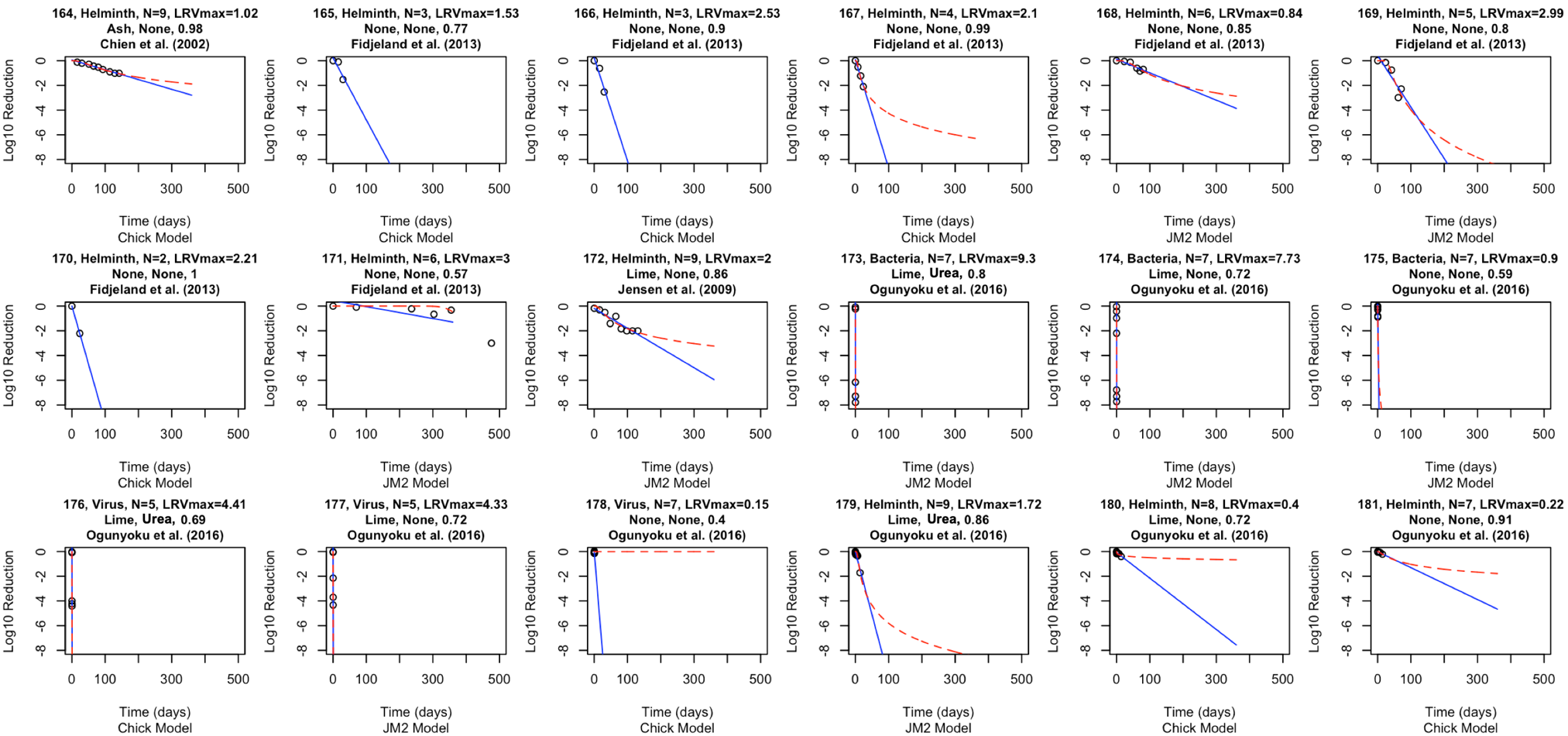

**Figure S1. Decay curves for experimental studies included in the meta-analysis to predict the decay rate coefficients (continued).**


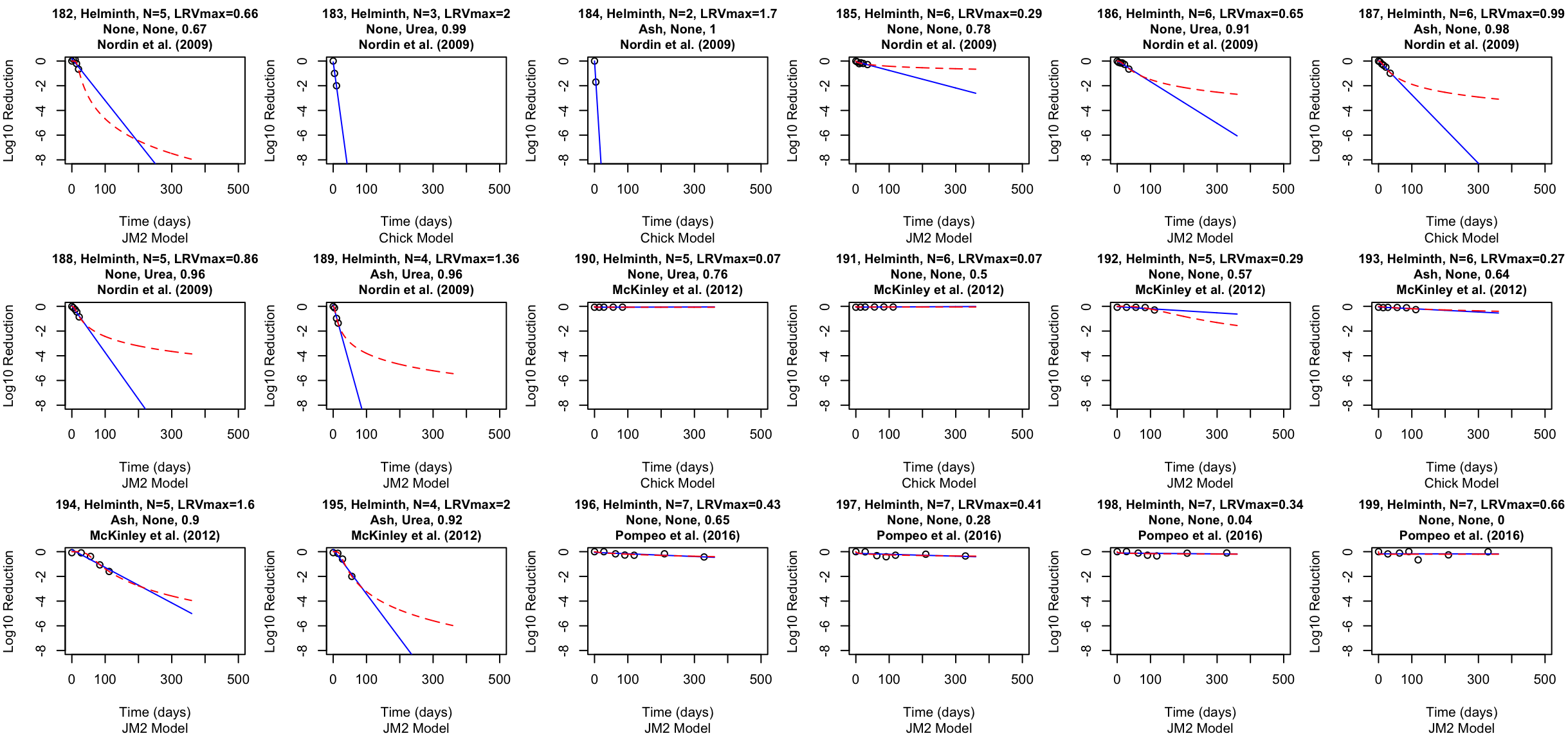

**Figure S1. Decay curves for experimental studies included in the meta-analysis to predict the decay rate coefficients (continued).**

**
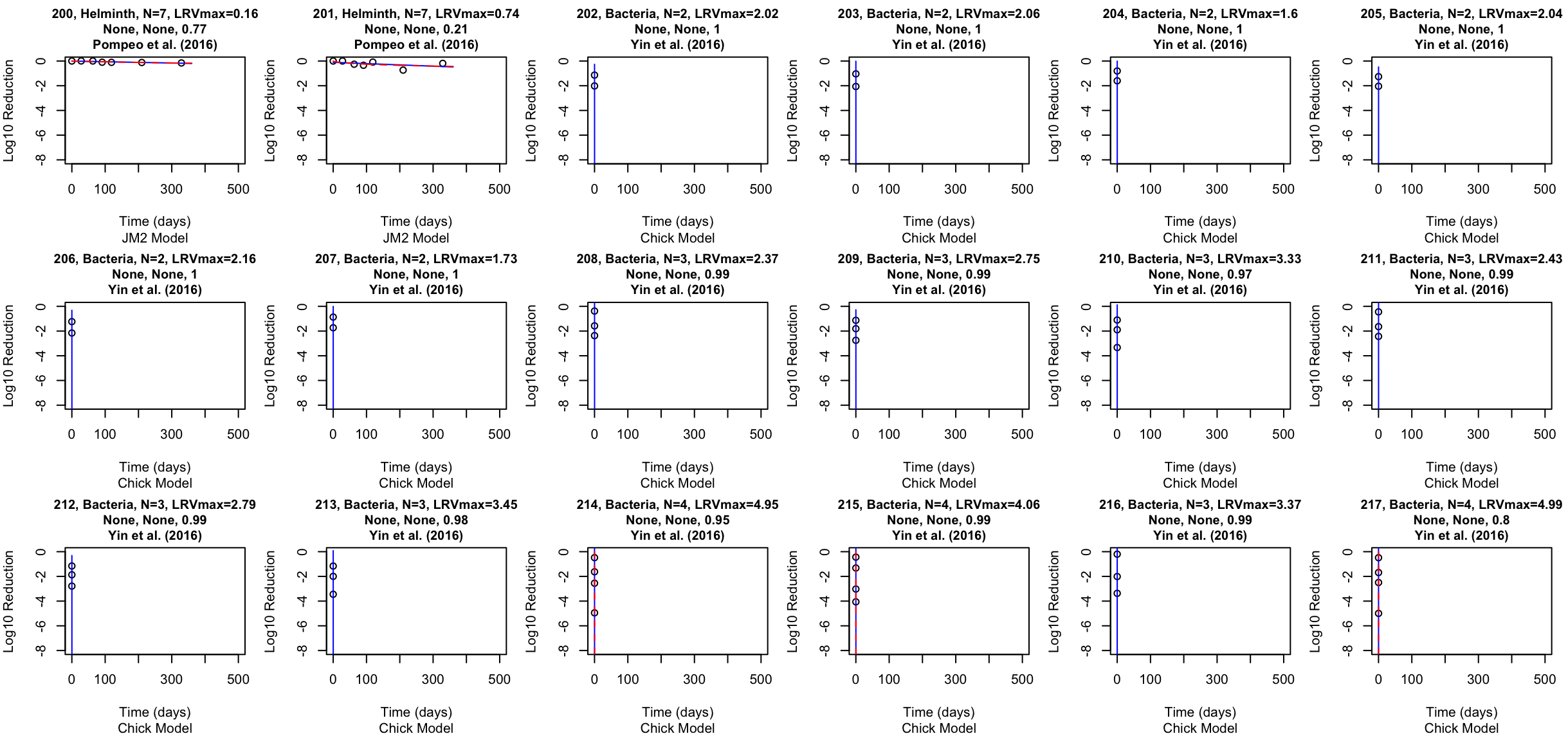
**

**Figure S1. Decay curves for experimental studies included in the meta-analysis to predict the decay rate coefficients (continued).**

**
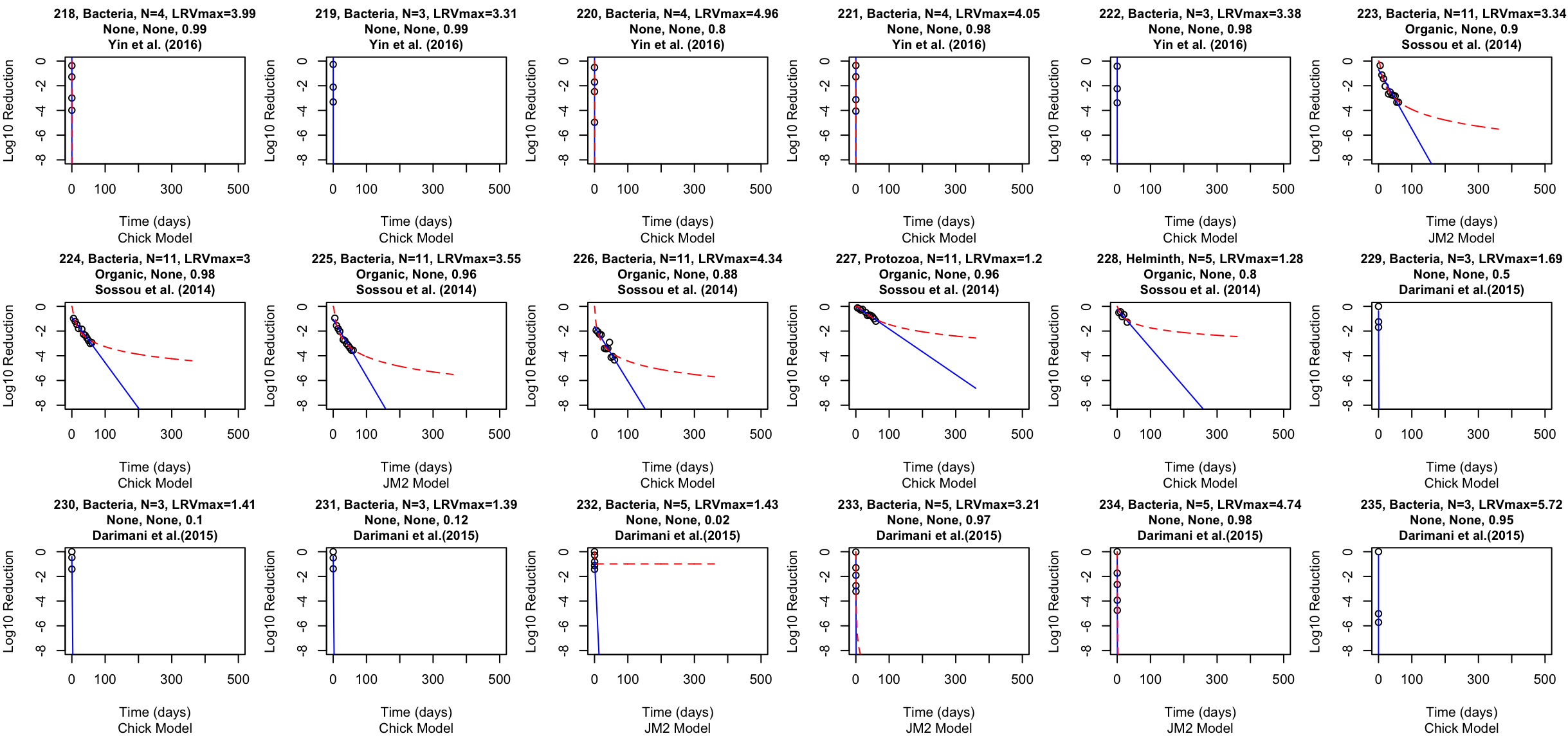
**

**Figure S1. Decay curves for experimental studies included in the meta-analysis to predict the decay rate coefficients (continued).**

**
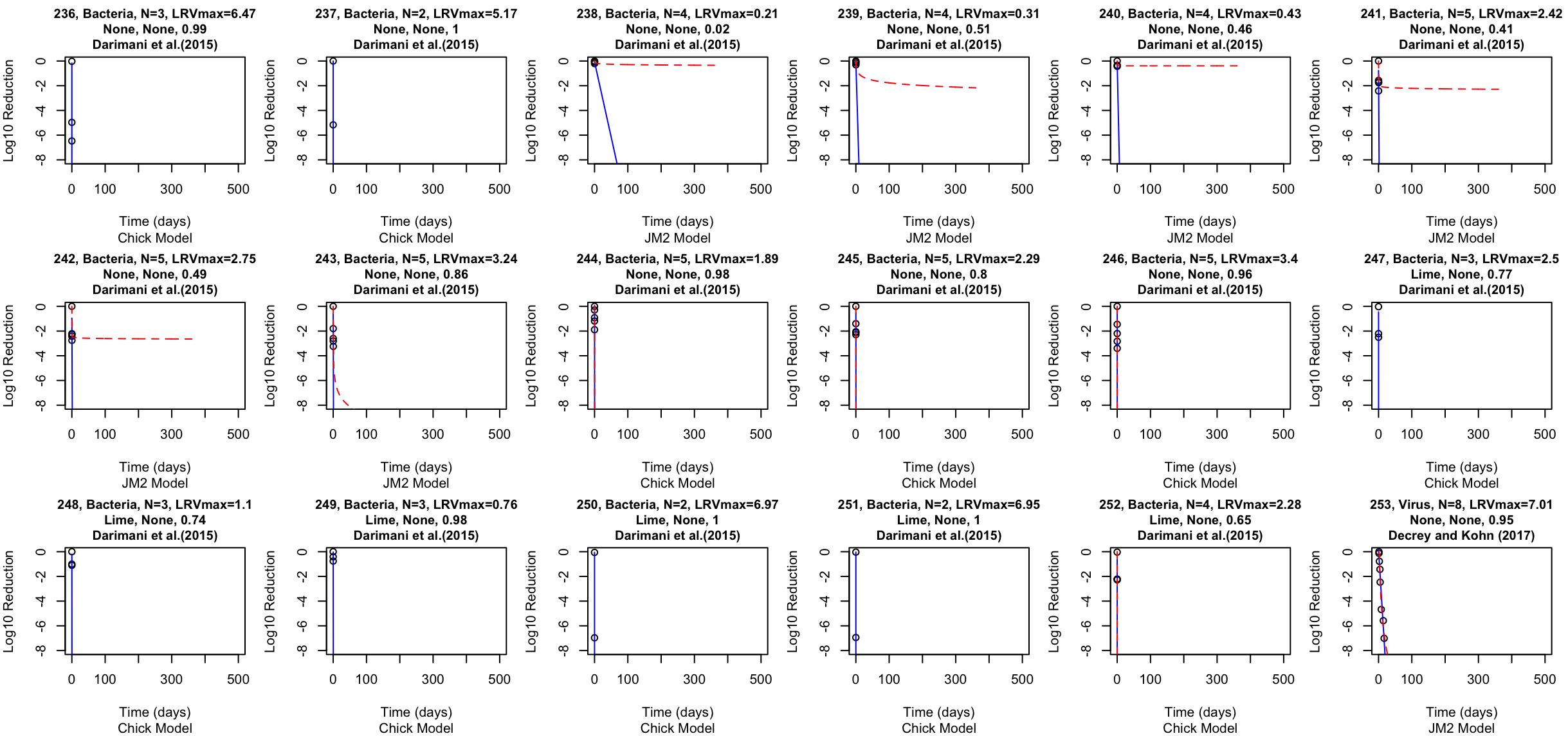
**

**Figure S1. Decay curves for experimental studies included in the meta-analysis to predict the decay rate coefficients (continued).**

**
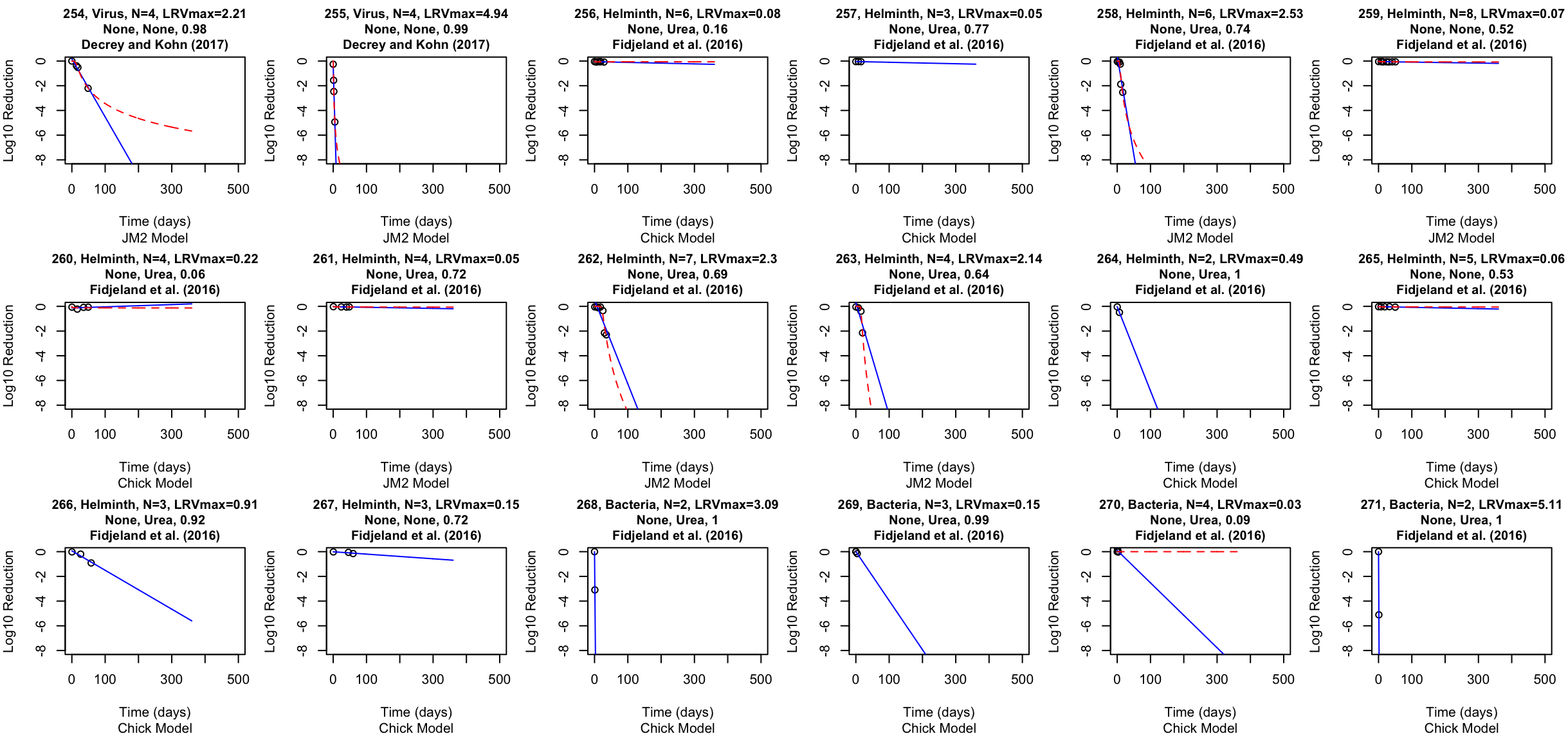
**

**Figure S1. Decay curves for experimental studies included in the meta-analysis to predict the decay rate coefficients (continued).**

**
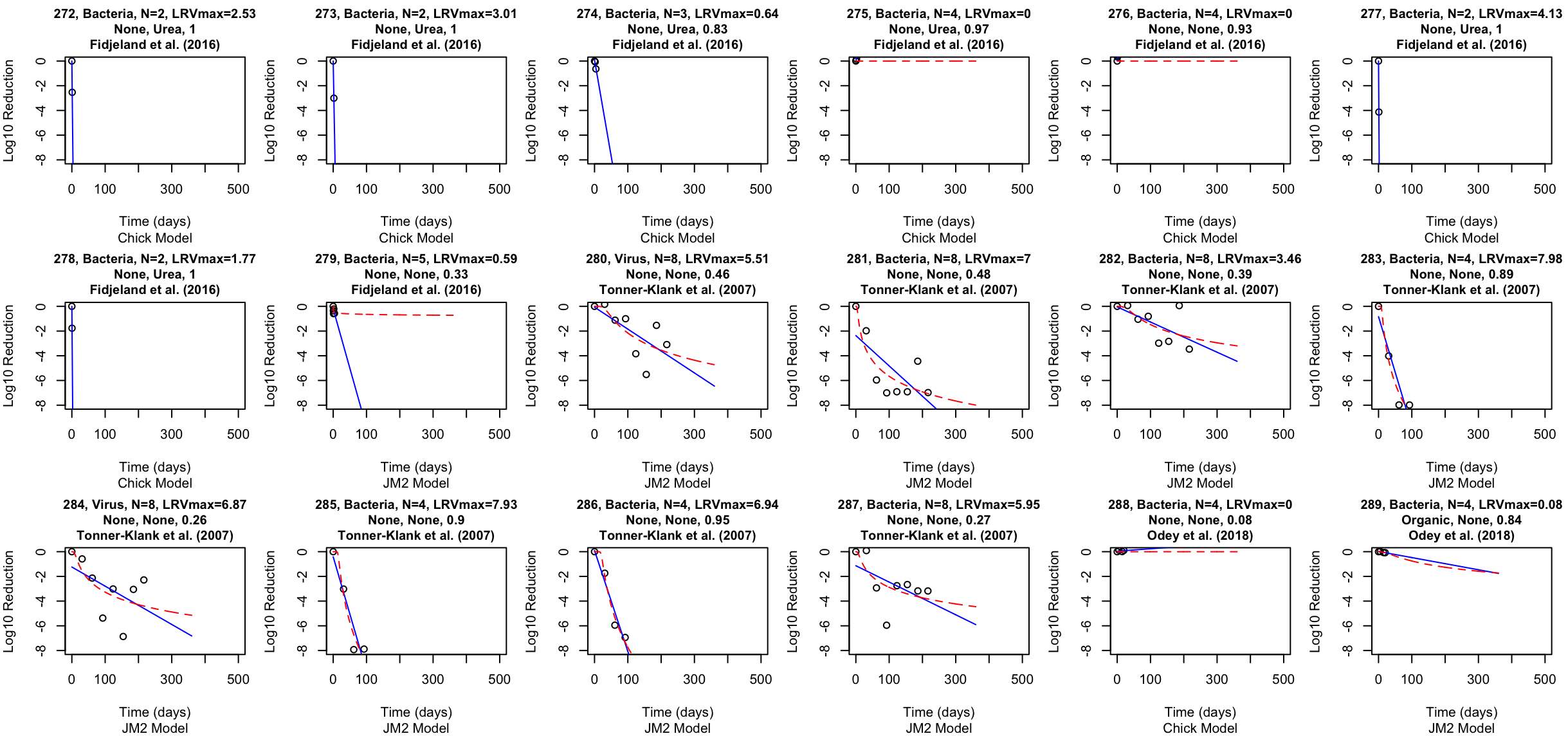
**

**Figure S1. Decay curves for experimental studies included in the meta-analysis to predict the decay rate coefficients (continued).**

**
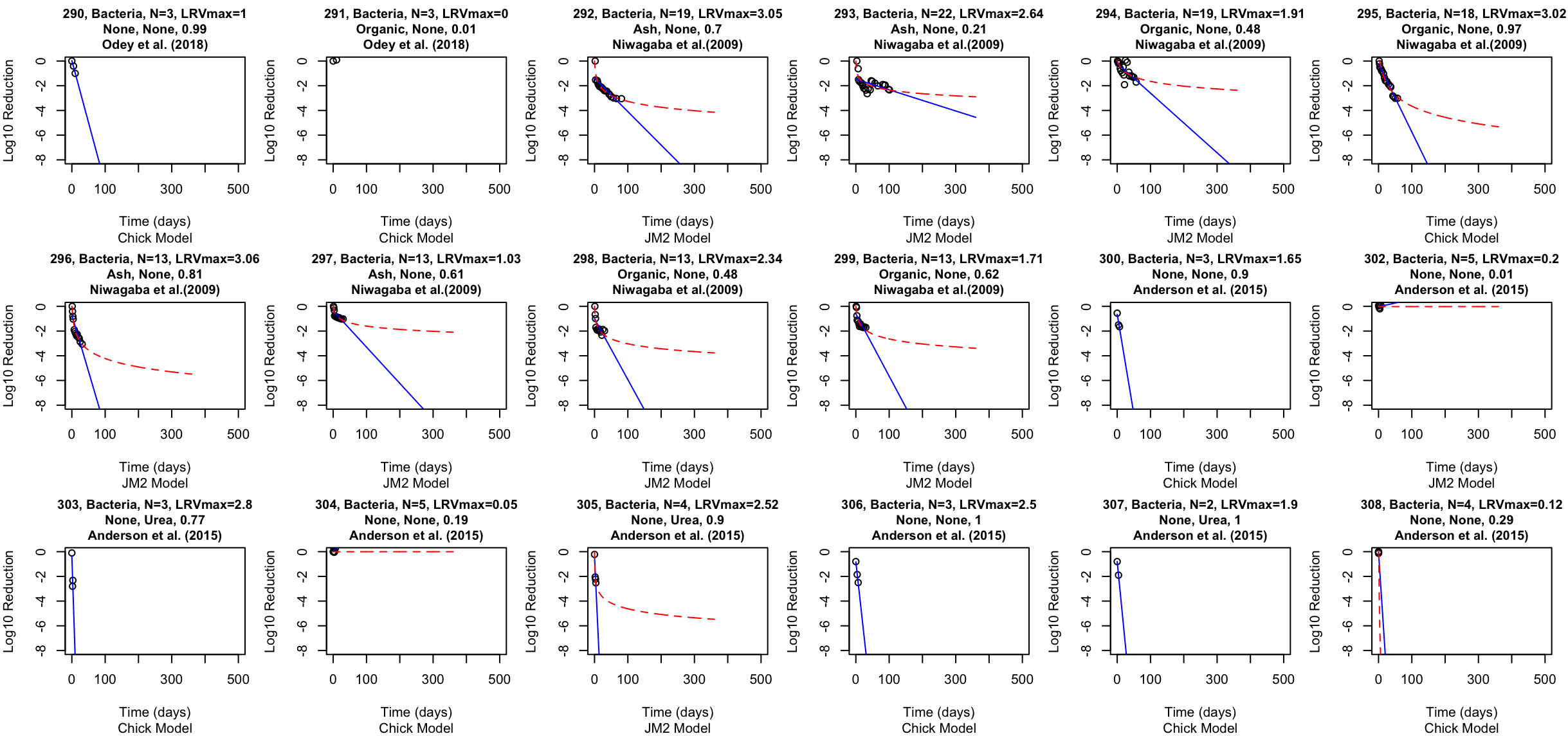
**

**Figure S1. Decay curves for experimental studies included in the meta-analysis to predict the decay rate coefficients (continued).**

**
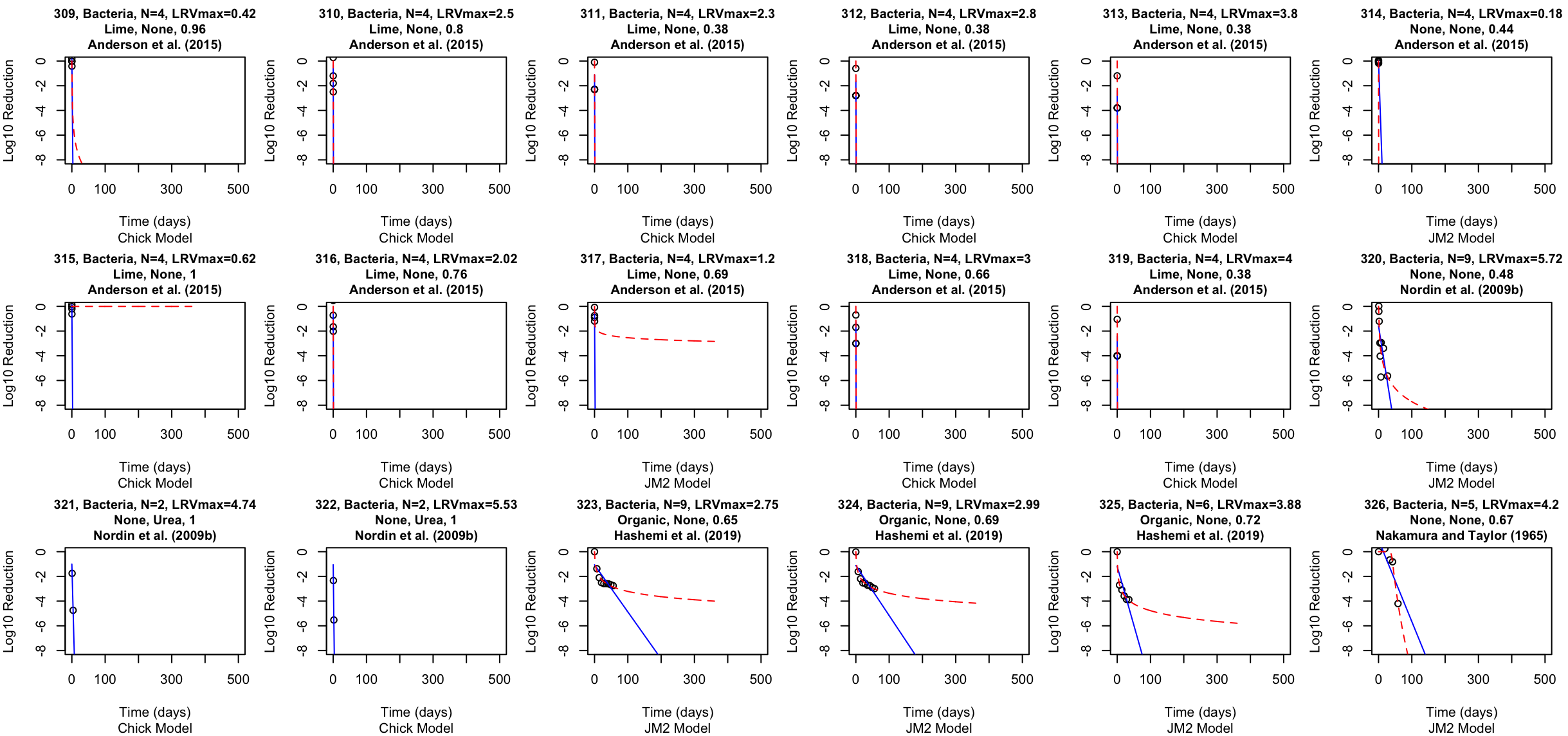
**

**Figure S1. Decay curves for experimental studies included in the meta-analysis to predict the decay rate coefficients (continued).**

**
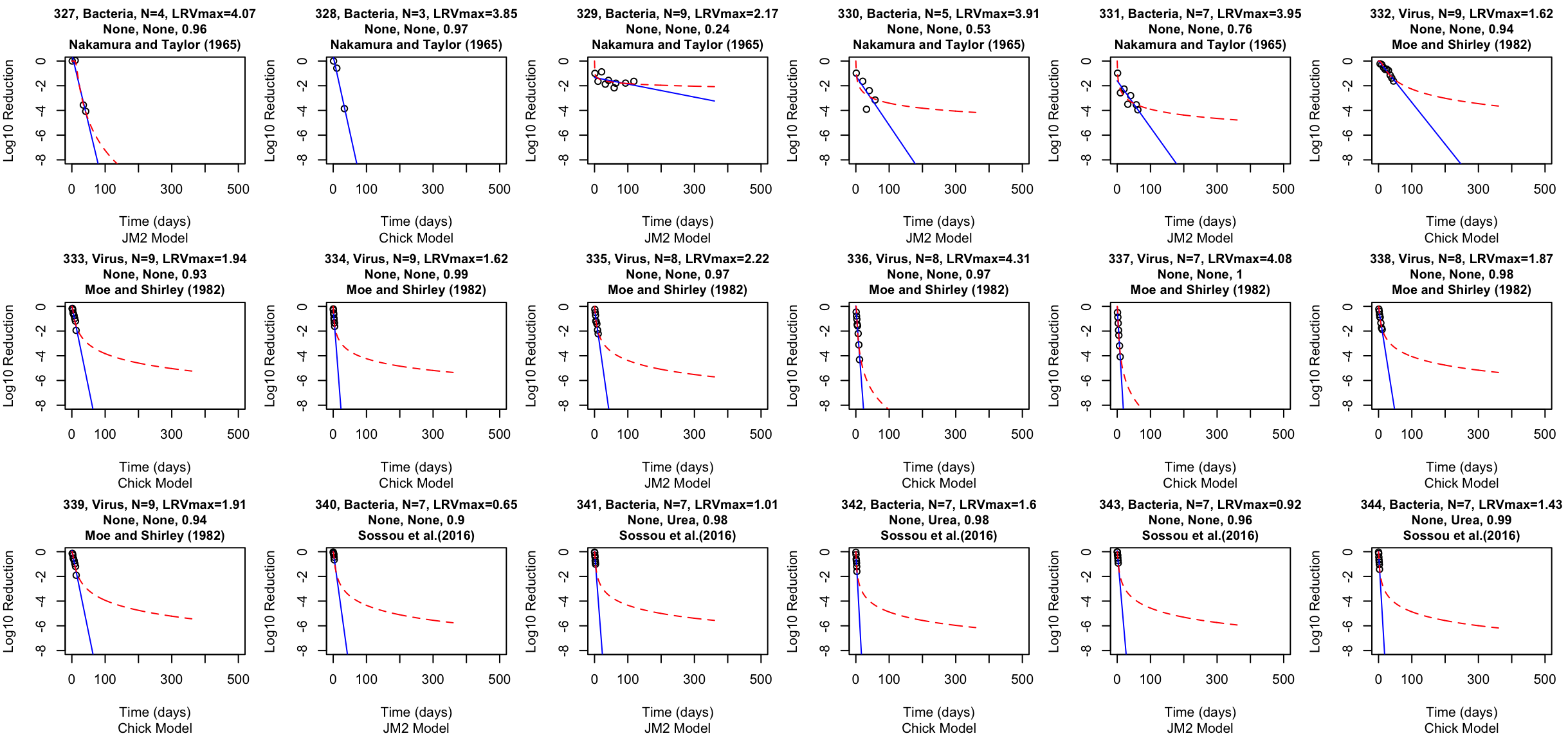
**

**Figure S1. Decay curves for experimental studies included in the meta-analysis to predict the decay rate coefficients (continued).**

**
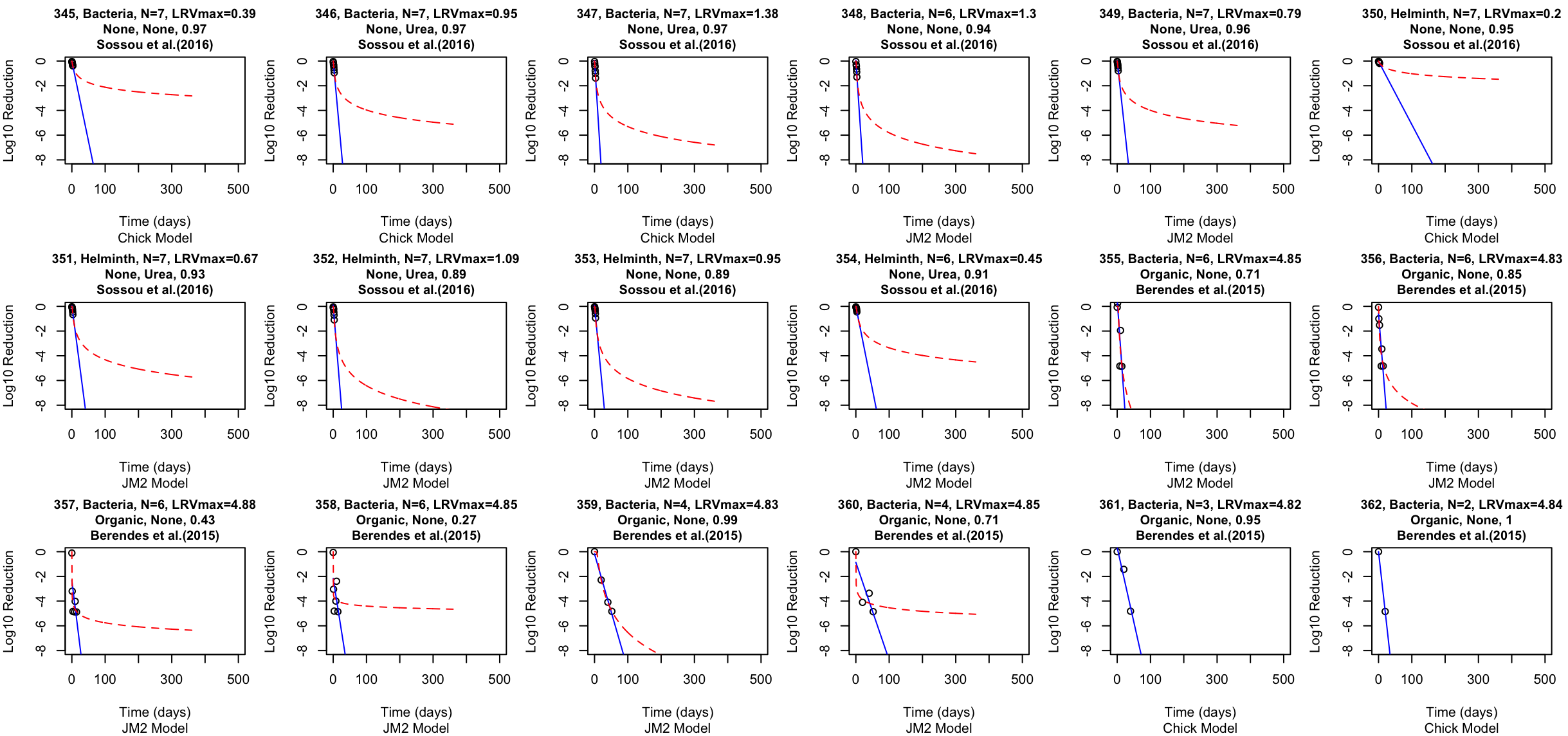
**

**Figure S1. Decay curves for experimental studies included in the meta-analysis to predict the decay rate coefficients (continued).**

**
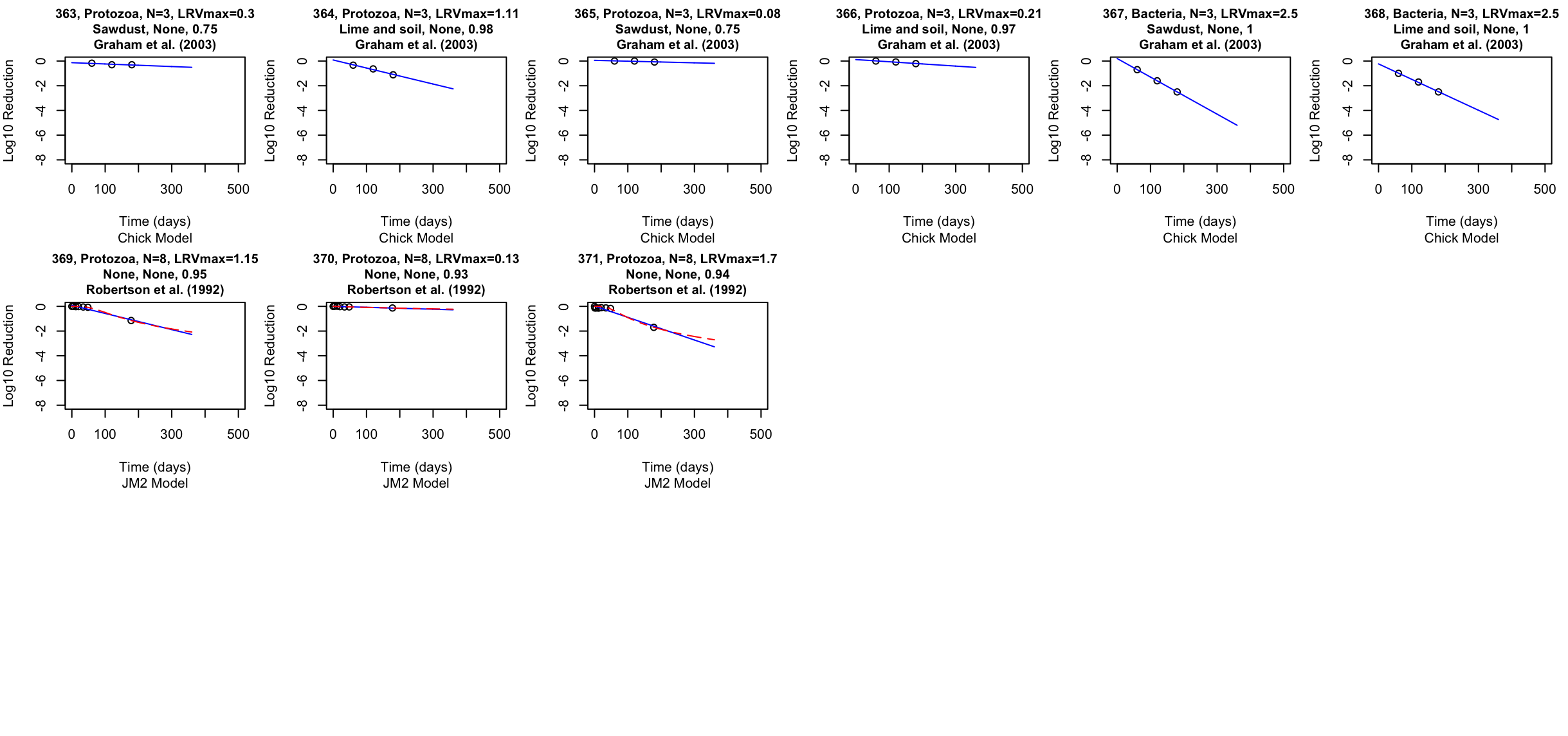
**

**Figure S1. Decay curves for experimental studies included in the meta-analysis to predict the decay rate coefficients (continued).**

**Table S1. Median and range of experimental conditions for each microbial group**

| **Microbial group** | **Number of experiments** | **pH^*^** | **Moisture Content^*^ (%)** | **Temp.^*^ (°C)** | **Urea Addition (%)** | **Urine-Diversion Toilets (%)** | **Desiccating or Alkalinizing Agents (%)** |
| --- | --- | --- | --- | --- | --- | --- | --- |
| Viruses | 32 | 7.8  (6.9 – 12.7) | 50 (9 – 93) | 22 (4 – 37) | 19% | 81% | 37%^a^ |
| Bacteria | 147 | 7.9 (4.3 – 12.7) | 70 (5 – 99) | 32 (-20 – 70) | 20% | 59% | 37%^b^ |
| Protozoa | 8 | 7.5 (6.0 – 9.8) | 25 (9 – 90) | 37 (32 – 70) | 0% | 75% | 62%^c^ |
| Helminths | 56 | 9.0 (4.4 – 12.8) | 83 (3 – 99) | 28 (10 – 34) | 32% | 45% | 29%^d^ |

^*^ Median values are shown (with ranges shown in parentheses)

^a^ Most common additives were ash (83%), oyster shells (50%), and lime (17%). Sum of percentages may exceed 100% because some studies used multiple additives in combination (e.g., oyster shells and ash).

^b^ Most common additives were organics (39%), lime (37%), ash (15%), oyster shells (12%), sawdust (2%) and soil (2%). Sum of percentages may exceed 100% because some studies used multiple additives in combination (e.g., oyster shells and ash).

^c^ Most common additives were lime and soil (40%), sawdust (40%), and organics (20%).

^d^ Most common additives were ash (62%), lime (25%), organics (6%), and soil (6%).

**Table S2. Summary of quality scores given for experiments done with each microbial group**

| **Microbial group** | **Number of Experiments** | | | | | |
| --- | --- | --- | --- | --- | --- | --- |
|  | **Total** | **Quality Score Less Than 1** | **Quality Score Between 1 – 2** | **Quality Score Between 2 – 3** | **Quality Score Equal to 3** |  |
| Viruses | 32 | 4 (13%) | 8 (25%) | 0 | 20 (63%) |  |
| Bacteria | 147 | 27 (18%) | 43 (29%) | 26 (18%) | 51 (35%) |  |
| Protozoa | 8 | 0 | 8 (100%) | 0 | 0 |  |
| Helminths | 56 | 3 (5%) | 7 (13%) | 21 (38%) | 25 (45%) |  |
| **TOTALS** | **243** | **34 (14%)** | **66 (27%)** | **47 (19%)** | **96 (40%)** |  |


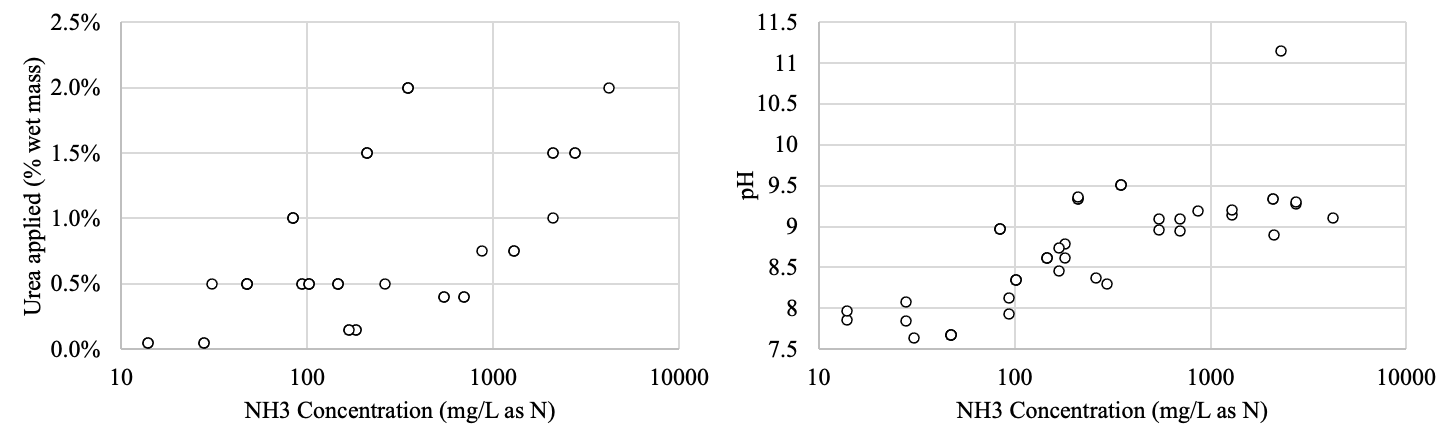


**Figure S2. Plot of the NH_3_ concentration with respect to the amount of urea applied (left) and the pH of the fecal sludge (right), for the experiments where urea was added.**


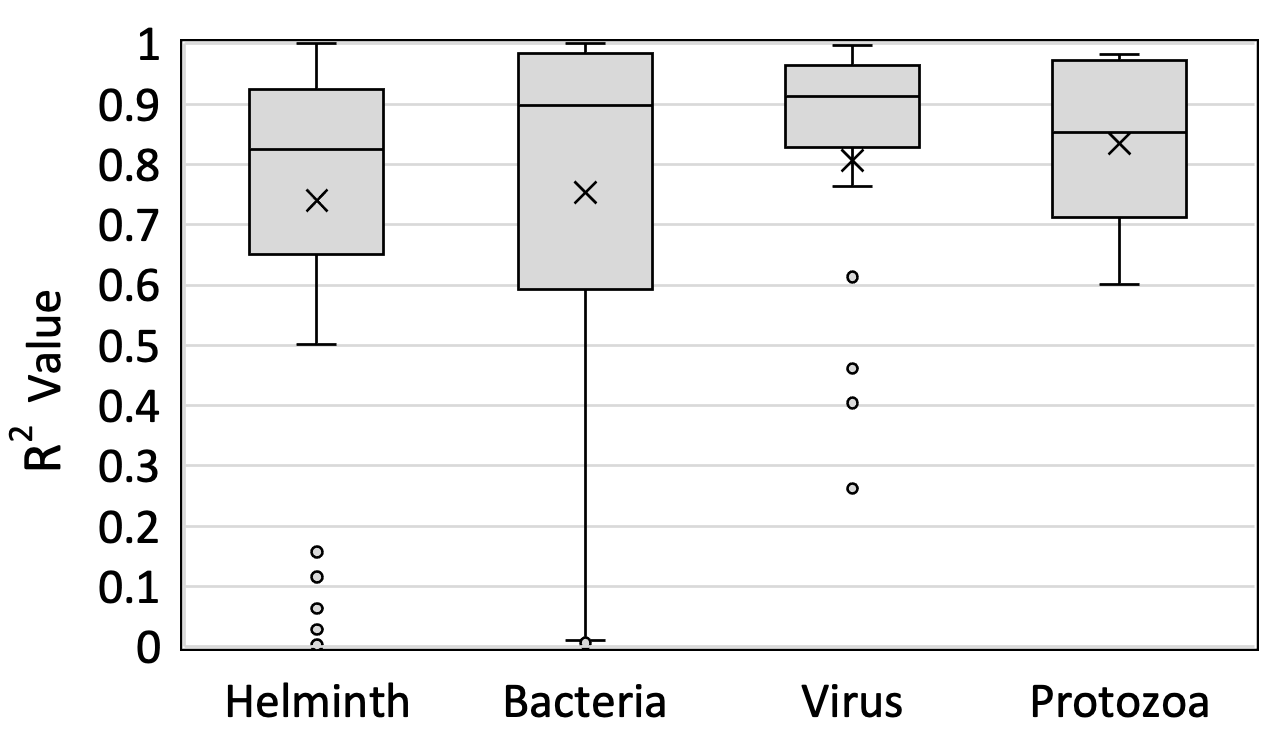


**Figure S3. Summary of R^2^ values achieved from fitting log-linear decay curves to experimental data for the different microbial groups**

**Figure S4. Histograms of the decay rate coefficients and their natural log or Box-Cox transformations: a) *k* (for the log-linear model); b) *k_1_* (for the JM2 model); and c) *k_2_* (for the JM2 model).**

**Figure S5. Summary of ln(k), *k*_1_, and *k*_2_ values achieved from fitting log-linear and JM2 models to experimental data for the different microbial groups**

**Figure S6. Natural log transformations of the JM2 model coefficients (panels a–f show results for coefficient *k*_1_ and panels g–l show results for coefficient *k*_2_.) obtained for experiments with protozoa and helminths (a–c and g–i) or with bacteria or viruses (d–f and j–l). Panels (a), (d), (g), and (j) show results from matrices common to urine diversion toilets (feces only) vs. matrices common to conventional toilets (excreta, i.e., urine mixed with feces); panels (b), (e), (h), and (k) show results when urea is used as a disinfectant; and panels (c), (f), (i), and (l) show the use of different additives for the desiccation or alkalinization of fecal sludge.**

**Figure S7. Correlation matrix of ln(*k*), *k*_1_, and** $\sqrt{\mathbf{ln}\left( \boldsymbol{k}_{\boldsymbol{2}} \right)}$ **with temperature (°C), pH, and moisture content (%) of the fecal sludge: a) including all experiments; and b) not including experiments performed at temperatures below 0°C or above 50°C.**

**^▪^ indicates significance at the 0.10 level**

*** indicates significance at the 0.05 level**

**** indicates significance at the 0.01 level**

***** indicates significance at the 0.001 level**


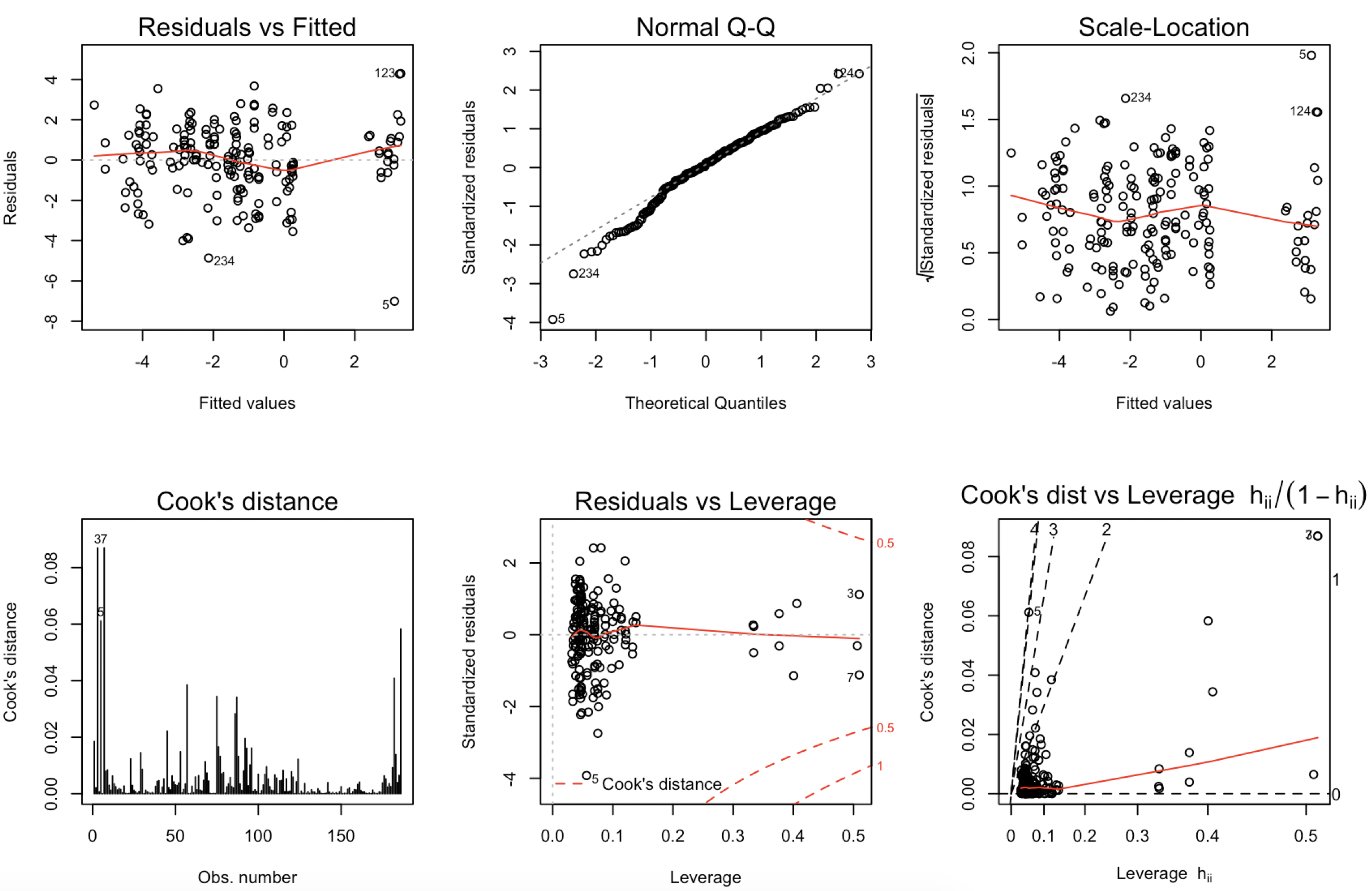


**Figure S8. Plot diagnostics for the multiple linear regression model for predicting the decay rate coefficient *k* for the log-linear Chick model**


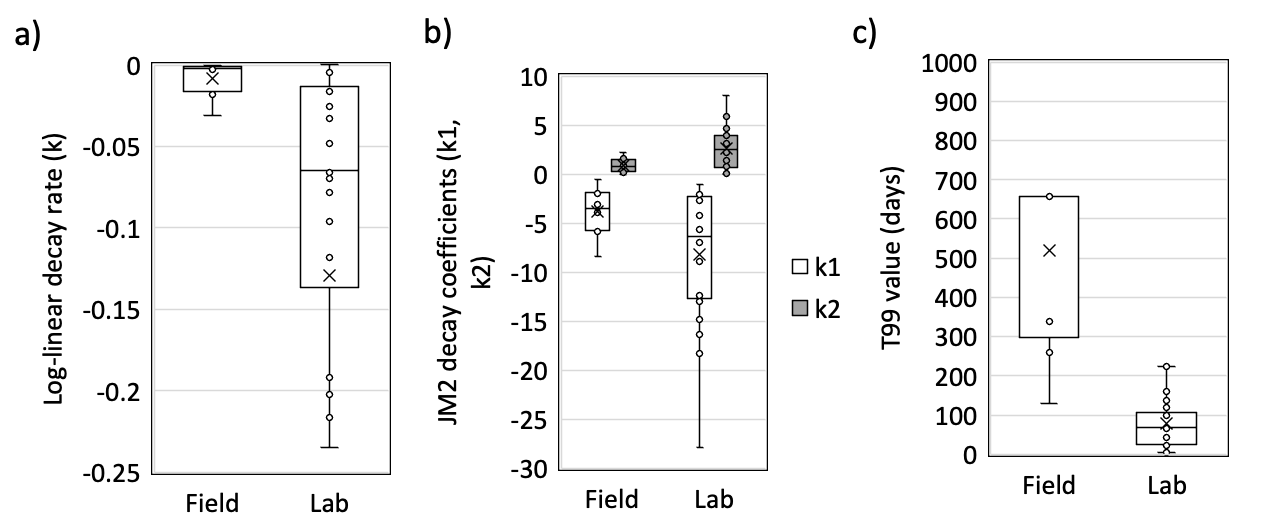


**Figure S9. Box plots showing estimated decay rate coefficients for a) the log-linear model and b) the JM2 model, along with c) estimated T_99_ values (days) based on the log-linear model from experiments done with *Ascaris* eggs in controlled laboratory setting (N=26) vs. field studies (N=9). If T_99_ could not be extrapolated within 2x the length of the experiment using the best fit model, then the censored T_99_ values were substituted with a value equal to 2x the length of the experiment.**

**
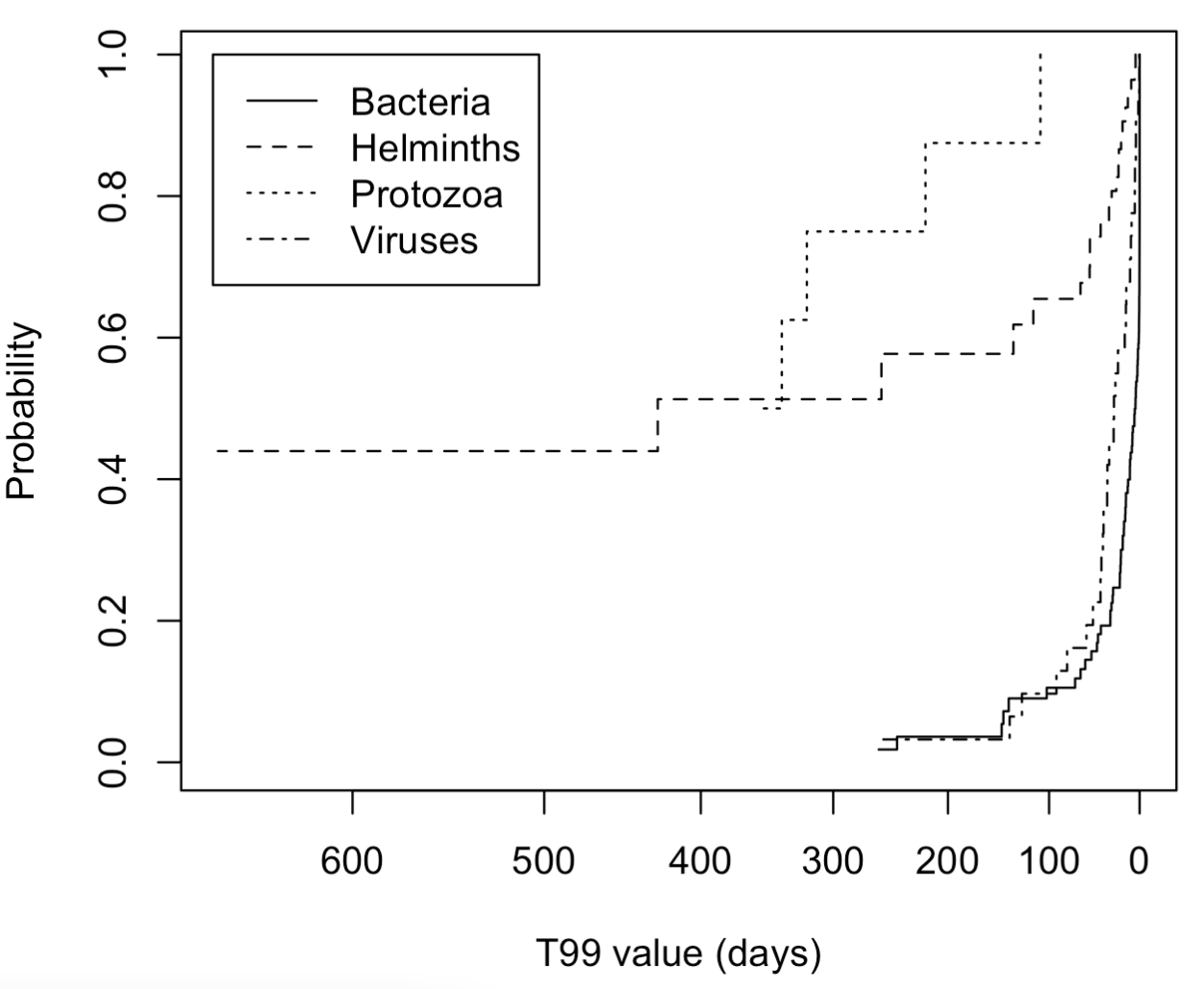
**

**Figure S10. Plot of empirical distribution function (EDFs) for T_99_ values (log scale) for the four pathogen groups.**

**Table S3. PRISMA guidelines checklist**

| **Topic** | **No.** | **Checklist Item Description** | **Where Item is Reported** |
| --- | --- | --- | --- |
| Title | 1 | Identify the report as a systematic review. | Title identifies the study as a systematic review and meta-analysis |
| Abstract | 2 | According to the PRISMA 2020 for Abstracts checklist:   - Provide an explicit statement of the main objective(s) or question(s) the review addresses. - Specify the inclusion and exclusion criteria for the review. - Specify the information sources (e.g. databases, registers) used to identify studies and the date when each was last searched. - Specify the methods used to assess risk of bias in the included studies. - Specify the methods used to present and synthesize results. - Give the total number of included studies and participants and summarize relevant characteristics of studies. - Present results for main outcomes, preferably indicating the number of included studies and participants for each. If meta-analysis was done, report the summary estimate and confidence/credible interval. If comparing groups, indicate the direction of the effect (i.e. which group is favored). - Provide a brief summary of the limitations of the evidence included in the review (e.g. study risk of bias, inconsistency and imprecision). - Provide a general interpretation of the results and important implications. - Specify the primary source of funding for the review. - Provide the register name and registration number. | Most of this information is contained within the abstract; anything that did not fit into the abstract is found in the manuscript. |
| Rationale | 3 | Describe the rationale for the review in the context of existing knowledge. | In the Introduction section |
| Objectives | 4 | Provide an explicit statement of the objective(s) or question(s) the review addresses. | At the end of the Introduction section |
| Eligibility criteria | 5 | Specify the inclusion and exclusion criteria for the review and how studies were grouped for the syntheses. | Section 2.1 and in Musaazi et al. (2020)^[[1]](#footnote-1)^ |
| Information sources | 6 | Specify all databases, registers, websites, organizations, reference lists and other sources searched or consulted to identify studies. Specify the date when each source was last searched or consulted. | Section 2.1 |
| Search strategy | 7 | Present the full search strategies for all databases, registers and websites, including any filters and limits used. | Section 2.1 |
| Selection process | 8 | Specify the methods used to decide whether a study met the inclusion criteria of the review, including how many reviewers screened each record and each report retrieved, whether they worked independently, and if applicable, details of automation tools used in the process. | Section 2.2 |
| Data collection process | 9 | Specify the methods used to collect data from reports, including how many reviewers collected data from each report, whether they worked independently, any processes for obtaining or confirming data from study investigators, and if applicable, details of automation tools used in the process. | Section 2.3 |
| Data items | 10a | List and define all outcomes for which data were sought. Specify whether all results that were compatible with each outcome domain in each study were sought (e.g. for all measures, time points, analyses), and if not, the methods used to decide which results to collect. | Section 2.3 |
|  | 10b | List and define all other variables for which data were sought (e.g. participant and intervention characteristics, funding sources). Describe any assumptions made about any missing or unclear information. | Section 2.3 |
| Study risk of bias assessment | 11 | Specify the methods used to assess risk of bias in the included studies, including details of the tool(s) used, how many reviewers assessed each study and whether they worked independently, and if applicable, details of automation tools used in the process. | Section 2.3 |
| Effect measures | 12 | Specify for each outcome the effect measure(s) (e.g. risk ratio, mean difference) used in the synthesis or presentation of results. | Sections 2.4 and 2.5 |
| Synthesis methods | 13a | Describe the processes used to decide which studies were eligible for each synthesis (e.g. tabulating the study intervention characteristics and comparing against the planned groups for each synthesis (item #5)). | Last paragraph in Section 2.4 (i.e., data from some experiments were used for the regression but not for model selection) |
|  | 13b | Describe any methods required to prepare the data for presentation or synthesis, such as handling of missing summary statistics, or data conversions. | Section 2.4 |
|  | 13c | Describe any methods used to tabulate or visually display results of individual studies and syntheses. | Figure S1 visually shows the results of individual studies and syntheses. |
|  | 13d | Describe any methods used to synthesize results and provide a rationale for the choice(s). If meta-analysis was performed, describe the model(s), method(s) to identify the presence and extent of statistical heterogeneity, and software package(s) used. | Sections 2.4 and 2.5 (model fitting, correlation, and regression analysis) |
|  | 13e | Describe any methods used to explore possible causes of heterogeneity among study results (e.g. subgroup analysis, meta-regression). | Section 3.5 |
|  | 13f | Describe any sensitivity analyses conducted to assess robustness of the synthesized results. | Not performed |
| Reporting bias assessment | 14 | Describe any methods used to assess risk of bias due to missing results in a synthesis (arising from reporting biases). | Section 3.4 |
| Certainty assessment | 15 | Describe any methods used to assess certainty (or confidence) in the body of evidence for an outcome. | Methods section  Supplemental Information (Optimization Procedure for Maximum Likelihood Estimation) |
| Study selection | 16a | Describe the results of the search and selection process, from the number of records identified in the search to the number of studies included in the review, ideally using a flow diagram. | Section 3.1; Figure 1 |
|  | 16b | Cite studies that might appear to meet the inclusion criteria, but which were excluded, and explain why they were excluded. | Supplemental Information (Table S4) |
| Study characteristics | 17 | Cite each included study and present its characteristics. | Section 3.1 |
| Risk of bias in studies | 18 | Present assessments of risk of bias for each included study. | Section 3.1 |
| Results of individual studies | 19 | For all outcomes, present, for each study: (a) summary statistics for each group (where appropriate) and (b) an effect estimate and its precision (e.g. confidence/credible interval), ideally using structured tables or plots. | Tables 1 and 2 |
| Results of syntheses | 20a | For each synthesis, briefly summarize the characteristics and risk of bias among contributing studies. | Sections 3.1 and 3.2 |
|  | 20b | Present results of all statistical syntheses conducted. If meta-analysis was done, present for each the summary estimate and its precision (e.g. confidence/credible interval) and measures of statistical heterogeneity. If comparing groups, describe the direction of the effect. | Sections 3.4 and 3.5 |
|  | 20c | Present results of all investigations of possible causes of heterogeneity among study results. | Sections 3.2 and 3.3 |
|  | 20d | Present results of all sensitivity analyses conducted to assess the robustness of the synthesized results. | Not performed |
| Reporting biases | 21 | Present assessments of risk of bias due to missing results (arising from reporting biases) for each synthesis assessed. | Section 3.7 |
| Certainty of evidence | 22 | Present assessments of certainty (or confidence) in the body of evidence for each outcome assessed. | Section 3.4 |
| Discussion | 23a | Provide a general interpretation of the results in the context of other evidence. | Results section |
|  | 23b | Discuss any limitations of the evidence included in the review. | The limitations associated with the lack of data for protozoan pathogens have been mentioned throughout the manuscript, also in the abstract. |
|  | 23c | Discuss any limitations of the review processes used. | Section 3.7 |
|  | 23d | Discuss implications of the results for practice, policy, and future research. | Conclusions section |
| Registration and protocol | 24a | Provide registration information for the review, including register name and registration number, or state that the review was not registered. | Section 2.1  Before the review began, the protocol was registered in PROSPERO (record no. CRD42020167254) |
|  | 24b | Indicate where the review protocol can be accessed, or state that a protocol was not prepared. | The protocol can be accessed at: [www.crd.york.ac.uk/prospero/display_record.php?ID=CRD42020167254](http://www.crd.york.ac.uk/prospero/display_record.php?ID=CRD42020167254) |
|  | 24c | Describe and explain any amendments to information provided at registration or in the protocol. | There were no amendments made |
| Support | 25 | Describe sources of financial or non-financial support for the review, and the role of the funders or sponsors in the review. | Acknowledgements section |
| Competing interests | 26 | Declare any competing interests of review authors. | There are no competing interests to declare |
| Availability of data, code and other materials | 27 | Report which of the following are publicly available and where they can be found: template data collection forms; data extracted from included studies; data used for all analyses; analytic code; any other materials used in the review. | Section 2.3 |

Reference: Page MJ, McKenzie JE, Bossuyt PM, Boutron I, Hoffmann TC, Mulrow CD, et al. The PRISMA 2020 statement: an updated guideline for reporting systematic reviews. BMJ 2021;372:n71. doi: 10.1136/bmj.n71

**Table S4. Summary of articles that met the inclusion criteria and from which data were extracted for the meta-analysis**

| **Reference** | **Number of experiments** | | | | **Characteristics of the experiments** |
| --- | --- | --- | --- | --- | --- |
|  | **Bacteria** | **Virus** | **Protozoa** | **Helminth** |  |
| Anderson et al. (2015) | 19 |  |  |  | Field-based experiments done in Malawi with *E. coli*, fecal coliforms, and total coliforms; lime and/or urea were added to excreta in some experiments. |
| Berendes et al. (2015) | 8 |  |  |  | Field-based experiments done in Haiti with *E. coli*; feces were co-composted with sugarcane husk in all experiments. The pH values were not reported, so they were imputed using the ‘mice’ package in R (van Buuren 2021), which produced estimated values that ranged between pH 7 and pH 9. |
| Chien et al. (2002) |  | 4 |  | 3 | Field-based experiments done in Vietnam with *Salmonella typhimurium* phages and *Ascaris suum* eggs; ash was added to feces in all experiments. |
| Darimani et al. (2015) | 24 |  |  |  | Lab-based experiments done in Burkina Faso with *E. coli* and *Enterococcus* bacterial indicators; lime was added to the feces in some experiments. |
| Decrey and Kohn (2017) |  | 3 |  |  | Lab-based experiments done in Switzerland with MS2 coliphage, T4 coliphage, and adenovirus in excreta; no additives were used. |
| Endale et al. (2012) | 4 |  |  | 4 | Lab-based experiments done in Ethiopia with *Ascaris* eggs and fecal coliforms; feces were mixed with either lime, ash, or soil, and a control received no additives. |
| Fidjeland et al. (2013) |  |  |  | 7 | Lab-based experiments done in Sweden with *Ascaris* eggs; no additives were used. |
| Fidjeland et al. (2016) | 12 |  |  | 12 | Lab-based experiments done with *Ascaris* eggs and *Salmonella*; urea was added to excreta in some experiments. |
| Graham et al. (2003) | 2 |  | 4 |  | Field-based experiments done in Mexico with fecal coliforms, *Giardia*, and *Cryptosporidium*; either sawdust or lime and soil were added to either excreta or feces. |
| Hashemi et al. (2019) | 3 |  |  |  | Lab-based experiments done in Korea with *E. coli*; feces were mixed with sawdust and charcoal produced from rice husks in all experiments. |
| Jensen et al. (2009) |  |  |  | 1 | Field-based experiment done in Vietnam with *Ascaris* eggs; lime was added to excreta. |
| Magri et al. (2013) | 10 | 12 |  |  | Lab-based experiments done in Sweden with *Enterococcus faecalis*; oyster shells and urea were added to the feces in some experiments. |
| McKinley et al. (2012) |  |  |  | 6 | Lab-based experiments done in Bolivia with *Ascaris suum* eggs; ash and/or urea were added to excreta or feces in the experiments. |
| Moe and Shirley (1982) |  | 8 |  |  | Lab-based experiments done in Great Britain with rotavirus; no additives were used. |
| Nakamura and Taylor (1965) | 6 |  |  |  | Lab-based experiments done in the United States with *Shigella sonnei* and *Shigella flexneri*; experiments were done with feces at temperatures that were near freezing. |
| Niwagaba et al. (2009) | 8 |  |  |  | Lab-based experiments done in Uganda with *E. coli* and enterococci; either ash or sawdust were added to feces in the experiments. |
| Nordin et al. (2009a) |  |  |  | 8 | Lab-based experiments done in Sweden with *Ascaris* eggs; ash and/or urea were added to feces in the experiments. |
| Nordin et al. (2009b) | 3 |  |  |  | Lab-based experiments done in Sweden with *Salmonella*; urea was added to feces in some of the experiments. |
| Odey et al. (2018) | 4 |  |  |  | Lab-based experiments done with fecal coliforms; Lactobacillus bacteria, cassava, and rice were added to excreta in some of the experiments to induce lactic acid fermentation. |
| Ogunyoku et al. (2016) | 3 | 3 |  | 3 | Lab-based experiments done in the United States with *E. coli*, MS2 coliphage, and *Ascaris* eggs; lime was added to excreta for some of the experiments. |
| Pompeo et al. (2016) |  |  |  | 6 | Field-based experiments done in Brazil with *Ascaris* eggs; no additives were used. |
| Robertson et al. (1992) |  |  | 3 |  | Lab-based experiments done in Scotland with *Cryptosporidium* oocysts; no additives were used. |
| Sossou et al. (2014) | 4 |  | 1 | 1 | Field-based experiments done in Japan with total coliforms, *E. coli*, streptococci, sulfur reducing clostridia, *Entamoeba* cysts, and *Ascaris* eggs; shea nut shells were added to feces for co-composting in all experiments. |
| Sossou et al. (2016b) | 10 |  |  | 5 | Lab-based experiments done in Burkina Faso with *E. coli*, *Enterococcus faecalis*, and *Ascaris lumbricoides*; urea was added to feces for some of the experiments. |
| Tonner-Klank et al. (2007) | 6 | 2 |  |  | Lab-based experiments done with *Salmonella typhimurium* phage 28B, thermotolerant coliforms, enterococci, and *Salmonella senftenberg*; no additives were used. |
| Yin et al. (2016) | 21 |  |  |  | Lab-based experiments done in China with fecal coliforms, streptococci, and *Salmonella*; no additives were used. |
| **TOTAL** | **147** | **32** | **8** | **56** | **243 total experiments** |

**Table S5. Examples of articles that met the inclusion criteria based on title/abstract but were excluded after reviewing the full text**

| **Citation** | **Reason for Exclusion** |
| --- | --- |
| Kazama, S.; Tameike, N.; Nakagawa, N.; Otaki, M. 2011. A fate model of pathogenic viruses in a composting toilet based on coliphage inactivation. *Journal of Environmental Sciences*, 23(7), 1194-1198. | Upon review of the methodology, the experimental set up was that of a flow-through reactor, so there was loss of coliphage as it exited the reactor through the outlet. Without knowing the residence time distribution, it would be impossible to accurately estimate true decay rate coefficients. Therefore, this study was not included in the meta-analysis. |
| Dey, D.; Ridwanul, A.T.M. Haque; Kabir, B.; Ubaid, S.F. 2016. Fecal indicator and Ascaris removal from double pit latrine content. *Journal of Water and Health*, 14(6), 972-979. | Upon careful review of the methodology, the experimental trial was performed by subjecting a composite fecal sludge sample to sun drying in the summer for two months, during which the samples were analyzed at regular intervals. This exposure to sunlight is not typical of pit latrines or other onsite sanitation systems, therefore this study was not included in the meta-analysis. |
| Kone, D.; Cofie, O.; Zurbrugg, C.; Gallizzi, K.; Moser, D.; Drescher, S.; Strauss, M. 2007. Helminth eggs inactivation efficiency by faecal sludge dewatering and co-composting in tropical climates. *Water Research*, 41(19), 4397-4402. | In this study, fecal sludge was loaded onto drying beds or composting slabs in an outdoor environment, which simulates the treatment of fecal sludge after being removed from onsite pits or tanks, but not while it is contained within the pit or tank. As such, this study was not included in the meta-analysis. |
| Stramer, S.L.; Cliver, D.O. 1984. Septage treatments to reduce the numbers of bacteria and polioviruses. *Applied and Environmental Microbiology*, 48(3), 566-572. | This study was not included in the meta-analysis because the septage was treated with technical and analytical grade disinfectants including glutaraldehyde and hydrogen peroxide, which are not reflective of conditions in most onsite sanitation systems. |
| Redlinger, T.; Graham, J.; Corella-Barud, V.; Avitia, R. 2001. Survival of fecal coliforms in dry-composting toilets. *Applied and Environmental Microbiology*, 67(9), 4036-4040. | This study was not included because the authors did not measure the pathogen concentrations at the beginning of the experiment, they only had measurements after 3 months and 6 months of storage, which are only two time points. It was also not possible to match the 3-month concentrations with the corresponding 6-month concentrations due to the way the data were presented, so it was not even possible to include the two timepoints from these data in the meta-analysis. |
| Strauch, D. 1991. Survival of pathogenic micro-organisms and parasites in excreta, manure and sewage sludge. *Revue Scientifique et Technique*, 10(3), 813-846. | This study was excluded because it was a review paper and did not present any new original data. |

**Optimization Procedure for Maximum Likelihood Estimation**

As mentioned in the Methods section of the main manuscript, maximum likelihood estimation with unbounded optimization was used to find the best fit parameters (*k*_1_ and *k*_2_) for the JM2 model (Juneja et al. 2006). Portable Fortran programs for numerical computation (PORT) routines for function minimization (Fox et al. 1978) were implemented via the *nlminb* function and the *bbmle* package in R (Bolker, 2020). Regarding the assumptions of the optimization protocol, the ability for the computational algorithm to converge on the optimized values was contingent on having good initial guesses for *k*_1_, *k*_2_, and sigma (the standard deviation of the normal distribution representing the difference between the measured and modeled log reduction values, which had a mean of zero and a standard deviation equal to sigma). After some trial and error, it was found that starting guesses of *k*_1_ = -5, *k*_2_ = 1, and sigma = 3 generally produced converging results for most experiments. However, for experimental data sets with very rapid decay (i.e., log-linear model *k* value less than -10), the initial guesses for *k*_1_ and *k*_2_ were each set to the value of *k*. For experimental data sets with very slow decay (i.e., log-linear model *k* value greater than -0.0001, the initial guesses were set to *k*_1_ = -5, *k*_2_ = 0.01, and sigma = 2. For other experiments that still did not converge under these conditions, initial guesses were set as follows: for experiments #175, 309, and 314, initial guesses were set to *k*_1_ = -0.1, *k*_2_ = 1, and sigma = 1; for experiment #242, initial guesses were set to *k*_1_ = 4, *k*_2_ = 100, and sigma = 1; for experiments #232, 239, 240, and 241, initial guesses were set to *k*_1_ = 5, *k*_2_ = 1, and sigma = 1; and for experiment #308, initial guesses were set to *k*_1_ = 1, *k*_2_ = 10, and sigma = 1.

**Table S6. Results of the model fitting for each experiment**

| **ID** | **Authors** | **Microbial group** | **Additive for Desiccation or Alkalinization** | **Urea Addition** | **Matrix**^f^ | **Log-Linear Model** | | **JM2 Model** | | | **Lower AIC** | **Maximum LRV** | **Maximum Time (days)** | **T_99_ value**^a^ **(days)** |
| --- | --- | --- | --- | --- | --- | --- | --- | --- | --- | --- | --- | --- | --- | --- |
|  |  |  |  |  |  | ***k* value (days^-1^)** | **SE of  *k* value** | ***k*_1_** | ***k*_2_** | **σ** |  |  |  |  |
| 128 | Endale et al. (2012) | *Ascaris* | Lime | None | Feces only | -0.0656 | 0.21 | -6.79 | 2.48 | 0.15 | JM2 | 1.0 | 40 | 98 |
| 129 | Endale et al. (2012) | *Ascaris* | Ash | None | Feces only | -0.0659 | 0.20 | -6.34 | 2.35 | 0.13 | JM2 | 1.0 | 40 | 104 |
| 130 | Endale et al. (2012) | *Ascaris* | Soil | None | Feces only | -0.0698 | 0.50 | -12.27 | 4.02 | 0.36 | JM2 | 1.0 | 40 | 67 |
| 131 | Endale et al. (2012) | *Ascaris* | None | None | Feces only | -0.0444 | 0.25 | -12.98 | 3.94 | 0.07 | JM2 | 0.7 | 40 | 87 |
| 132 | Endale et al. (2012) | Fecal coliforms | Lime | None | Feces only | -11.029 | NA | NA | NA | NA | NA | 4.8 | 1.0 | 0.42^b^ |
| 133 | Endale et al. (2012) | Fecal coliforms | Ash | None | Feces only | -0.4334 | 0.74 | -13.96 | 7.70 | 1.28 | log-linear | 6.0 | 30 | 11 |
| 134 | Endale et al. (2012) | Fecal coliforms | Soil | None | Feces only | -0.0676 | 0.32 | -10.41 | 3.58 | 0.34 | JM2 | 1.1 | 40 | 66 |
| 135 | Endale et al. (2012) | Fecal coliforms | None | None | Feces only | -0.0114 | 0.03 | -8.15 | 2.04 | 0.03 | JM2 | 0.2 | 40 | 510 |
| 136 | Magri et al. (2013) | *Enterococcus faecalis* | Oyster shells | Urea | Feces only | -0.1703 | 1.23 | -95.70 | 23.70 | 1.84 | log-linear | 6.0 | 99 | 61 |
| 137 | Magri et al. (2013) | *Enterococcus faecalis* | Oyster shells and ash | Urea | Feces only | -0.1490 | 1.21 | -91.45 | 22.11 | 2.68 | log-linear | 4.5 | 100 | 72 |
| 138 | Magri et al. (2013) | *Enterococcus faecalis* | Oyster shells and ash | Urea | Feces only | -0.2056 | 0.99 | -52.14 | 14.32 | 2.14 | log-linear | 5.9 | 100 | 55 |
| 139 | Magri et al. (2013) | *Enterococcus faecalis* | None | None | Feces only | -0.0132 | 0.69 | -6.86 | -5.61 | 3.26 | log-linear | -0.6 | 131 | >500^c^ |
| 140 | Magri et al. (2013) | *Enterococcus faecalis* | None | Urea | Feces only | -0.1024 | 0.75 | -75.74 | 17.41 | 2.65 | log-linear | 3.9 | 131 | 102 |
| 141 | Magri et al. (2013) | *S. enterica* Typhimurium | Oyster shells | Urea | Feces only | -0.5522 | 1.17 | -39.55 | 15.24 | 0.35 | JM2 | 6.7 | 36 | 18 |
| 142 | Magri et al. (2013) | *S. enterica* Typhimurium | Oyster shells and ash | Urea | Feces only | -0.6724 | 4.63 | -111.29 | 37.45 | 0.18 | JM2 | 7.0 | 30 | 22 |
| 143 | Magri et al. (2013) | *S. enterica* Typhimurium | Oyster shells | Urea | Feces only | -0.5318 | 2.54 | -15.24 | 8.18 | 1.37 | JM2 | 5.8 | 29 | 11 |
| 144 | Magri et al. (2013) | *S. enterica* Typhimurium | None | None | Feces only | -0.0610 | 1.25 | -7.38 | 3.41 | 1.19 | JM2 | 3.6 | 130 | 33 |
| 145 | Magri et al. (2013) | *S. enterica* Typhimurium | None | Urea | Feces only | -0.4463 | 2.96 | -55.08 | 19.41 | 1.92 | JM2 | 7.0 | 36 | 22 |
| 146 | Magri et al. (2013) | Bacteriophage ΦX | Oyster shells and ash | None | Feces only | -0.1677 | 0.98 | -18.10 | 6.69 | 0.65 | JM2 | 5.0 | 78 | 30 |
| 147 | Magri et al. (2013) | Bacteriophage ΦX | Oyster shells and ash | Urea | Feces only | -0.2488 | 0.61 | -19.48 | 7.61 | 0.67 | log-linear | 4.9 | 50 | 25 |
| 148 | Magri et al. (2013) | Bacteriophage ΦX | Oyster shells and ash | Urea | Feces only | -0.2100 | 0.78 | -24.03 | 8.47 | 0.77 | JM2 | 5.0 | 64 | 29 |
| 149 | Magri et al. (2013) | Bacteriophage ΦX | None | None | Feces only | -0.1081 | 0.98 | -27.21 | 7.98 | 1.47 | log-linear | 5.0 | 99 | 53 |
| 150 | Magri et al. (2013) | Bacteriophage ΦX | None | Urea | Feces only | -0.0309 | 0.25 | -5.24 | 1.94 | 0.59 | log-linear | 2.1 | 130 | 128 |
| 151 | Magri et al. (2013) | Bacteriophage ΦX | None | None | Feces only | -0.0261 | 0.38 | -2.97 | 1.41 | 0.50 | log-linear | 1.9 | 131 | 140 |
| 152 | Magri et al. (2013) | Bacteriophage MS2 | Oyster shells and ash | None | Feces only | -0.1254 | 1.01 | -24.64 | 8.11 | 0.74 | JM2 | 6.1 | 130 | 37 |
| 153 | Magri et al. (2013) | Bacteriophage MS2 | Oyster shells and ash | Urea | Feces only | -0.1277 | 1.10 | -22.94 | 7.76 | 1.04 | JM2 | 6.3 | 131 | 35 |
| 154 | Magri et al. (2013) | Bacteriophage MS2 | Oyster shells and ash | Urea | Feces only | -0.1600 | 1.52 | -34.89 | 10.93 | 1.37 | JM2 | 7.1 | 131 | 37 |
| 155 | Magri et al. (2013) | Bacteriophage MS2 | None | None | Feces only | -0.0614 | 1.32 | -12.43 | 4.48 | 0.90 | JM2 | 3.8 | 130 | 45 |
| 156 | Magri et al. (2013) | Bacteriophage MS2 | None | Urea | Feces only | -0.0247 | 0.74 | -2.27 | 1.56 | 0.53 | JM2 | 2.2 | 130 | 81 |
| 157 | Magri et al. (2013) | Bacteriophage MS2 | None | None | Feces only | -0.0074 | 0.10 | 0.30 | 0.41 | 0.18 | log-linear | 1.2 | 130 | 407 |
| 158 | Chien et al. (2002) | *S. typhimurium* phages | Ash | None | Feces only | -0.2392 | 1.61 | -60.09 | 17.41 | 1.94 | JM2 | 8.2 | 79 | 41 |
| 159 | Chien et al. (2002) | *S. typhimurium* phages | Ash | None | Feces only | -0.1062 | 0.53 | -18.05 | 6.73 | 1.83 | log-linear | 8.3 | 154 | 28 |
| 160 | Chien et al. (2002) | *S. typhimurium* phages | Ash | None | Feces only | -0.1314 | 0.57 | -11.98 | 5.67 | 1.49 | log-linear | 8.1 | 114 | 17 |
| 161 | Chien et al. (2002) | *S. typhimurium* phages | Ash | None | Feces only | -0.3655 | 2.49 | -16.58 | 9.50 | 1.67 | JM2 | 8.3 | 51 | 9.3 |
| 162 | Chien et al. (2002) | *Ascaris suum* | Ash | None | Feces only | -0.0310 | 0.39 | -3.50 | 1.41 | 0.16 | JM2 | 1.0 | 65 | 309 |
| 163 | Chien et al. (2002) | *Ascaris suum* | Ash | None | Feces only | -0.0147 | 0.21 | -5.62 | 1.59 | 0.22 | JM2 | 1.0 | 169 | 618 |
| 164 | Chien et al. (2002) | *Ascaris suum* | Ash | None | Feces only | -0.0182 | 0.09 | -8.41 | 2.17 | 0.12 | log-linear | 1.0 | 142 | 260 |
| 165 | Fidjeland et al. (2013) | *Ascaris* eggs | None | None | Excreta | -0.1155 | 1.24 | NA | NA | NA | NA | 1.5 | 30 | 44 |
| 166 | Fidjeland et al. (2013) | *Ascaris* eggs | None | None | Excreta | -0.1917 | 1.27 | NA | NA | NA | NA | 2.5 | 30 | 27 |
| 167 | Fidjeland et al. (2013) | *Ascaris* eggs | None | None | Excreta | -0.2019 | 0.22 | -6.94 | 3.64 | 0.22 | log-linear | 2.1 | 24 | 24 |
| 168 | Fidjeland et al. (2013) | *Ascaris* eggs | None | None | Excreta | -0.0255 | 0.29 | -12.34 | 3.22 | 0.22 | JM2 | 0.8 | 80 | 192 |
| 169 | Fidjeland et al. (2013) | *Ascaris* eggs | None | None | Excreta | -0.0959 | 1.29 | -27.91 | 8.05 | 0.89 | JM2 | 3.0 | 71 | 57 |
| 170 | Fidjeland et al. (2013) | *Ascaris* eggs | None | None | Excreta | -0.2165 | NA | NA | NA | NA | NA | 2.2 | 24 | 21 |
| 171 | Fidjeland et al. (2013) | *Ascaris* eggs | None | None | Excreta | -0.0111 | 1.40 | -129.26 | 22.08 | 0.65 | JM2 | 3.0 | 476 | 429 |
| 172 | Jensen et al. (2009) | *Ascaris* eggs | Lime | None | Excreta | -0.0368 | 0.44 | -7.42 | 2.53 | 0.59 | JM2 | 2.0 | 131 | 116 |
| 173 | Ogunyoku et al. (2016) | *E. coli* | Lime | None | Excreta | -60.337 | 3.65 | 42.68 | 20.71 | 2.93 | JM2 | 9.3 | 0.42 | 0.16 |
| 174 | Ogunyoku et al. (2016) | *E. coli* | Lime | None | Excreta | -51.003 | 3.80 | 41.17 | 24.05 | 2.58 | JM2 | 7.7 | 0.42 | 0.22 |
| 175 | Ogunyoku et al. (2016) | *E. coli* | None | None | Excreta | -4.8052 | 0.48 | 6.60 | 5.09 | 0.34 | JM2 | 0.9 | 0.42 | 0.67 |
| 176 | Ogunyoku et al. (2016) | MS2 coliphage | Lime | None | Excreta | -38.805 | 3.13 | 38.81 | 38.80 | 7.52 | log-linear | 4.4 | 0.29 | 0.15 |
| 177 | Ogunyoku et al. (2016) | MS2 coliphage | Lime | None | Excreta | -34.739 | 2.61 | 33.23 | 18.33 | 0.59 | JM2 | 4.3 | 0.29 | 0.21 |
| 178 | Ogunyoku et al. (2016) | MS2 coliphage | None | None | Excreta | -0.7522 | 0.11 | -4.11 | 0.00 | 0.24 | log-linear | 0.2 | 0.42 | 6.1 |
| 179 | Ogunyoku et al. (2016) | *Ascaris* | Lime | None | Excreta | -0.2347 | 0.20 | -8.94 | 4.84 | 0.34 | JM2 | 1.7 | 14 | 16 |
| 180 | Ogunyoku et al. (2016) | *Ascaris* | Lime | None | Excreta | -0.0479 | 0.07 | -1.08 | 0.40 | 0.16 | log-linear | 0.4 | 14 | 93 |
| 181 | Ogunyoku et al. (2016) | *Ascaris* | None | None | Excreta | -0.0297 | 0.03 | -4.21 | 1.41 | 0.05 | log-linear | 0.2 | 14 | 154 |
| 182 | Nordin et al. (2009a) | *Ascaris* | None | None | Feces only | -0.0783 | 0.38 | -16.33 | 5.89 | 0.21 | JM2 | 0.7 | 20 | 35 |
| 183 | Nordin et al. (2009a) | *Ascaris* | None | Urea | Feces only | -0.4549 | 0.33 | NA | NA | NA | NA | 2.0 | 10 | 10 |
| 184 | Nordin et al. (2009a) | *Ascaris* | Ash | None | Feces only | -0.9793 | NA | NA | NA | NA | NA | 1.7 | 4.0 | 4.7 |
| 185 | Nordin et al. (2009a) | *Ascaris* | None | None | Feces only | -0.0164 | 0.08 | -2.29 | 0.61 | 0.08 | JM2 | 0.3 | 35 | >500^c^ |
| 186 | Nordin et al. (2009a) | *Ascaris* | None | Urea | Feces only | -0.0389 | 0.11 | -6.56 | 2.17 | 0.13 | JM2 | 0.7 | 35 | 172 |
| 187 | Nordin et al. (2009a) | *Ascaris* | Ash | None | Feces only | -0.0639 | 0.08 | -5.69 | 2.17 | 0.12 | log-linear | 1.0 | 35 | 74 |
| 188 | Nordin et al. (2009a) | *Ascaris* | None | Urea | Feces only | -0.0872 | 0.13 | -6.12 | 2.55 | 0.09 | JM2 | 0.9 | 22 | 67 |
| 189 | Nordin et al. (2009a) | *Ascaris* | Ash | Urea | Feces only | -0.2247 | 0.29 | -4.81 | 2.95 | 0.10 | JM2 | 1.4 | 15 | 24 |
| 190 | McKinley et al. (2012) | *Ascaris* ova | None | Urea | Feces only | 0.0001 | 0.00 | -1.76 | 0.00 | 0.07 | log-linear | 0.1 | 84 | >500^c^ |
| 191 | McKinley et al. (2012) | *Ascaris* ova | None | None | Feces only | 0.0003 | 0.01 | -2.03 | 0.00 | 0.07 | log-linear | 0.1 | 112 | >500^c^ |
| 192 | McKinley et al. (2012) | *Ascaris* ova | None | None | Excreta | -0.0038 | 0.13 | -14.85 | 3.12 | 0.12 | JM2 | 0.3 | 112 | >500^c^ |
| 193 | McKinley et al. (2012) | *Ascaris* ova | Ash | None | Feces only | -0.0031 | 0.07 | -4.23 | 0.79 | 0.12 | log-linear | 0.3 | 112 | >500^c^ |
| 194 | McKinley et al. (2012) | *Ascaris* ova | Ash | None | Excreta | -0.0330 | 0.42 | -18.29 | 4.65 | 0.11 | JM2 | 1.6 | 112 | 137 |
| 195 | McKinley et al. (2012) | *Ascaris* ova | Ash | Urea | Feces only | -0.0830 | 0.54 | -15.32 | 4.94 | 0.14 | JM2 | 2.0 | 56 | 56 |
| 196 | Pompeo et al. (2016) | *Ascaris* eggs | None | None | Excreta | -0.0025 | 0.13 | -3.90 | 0.73 | 0.17 | JM2 | 0.4 | 329 | >500^c^ |
| 197 | Pompeo et al. (2016) | *Ascaris* eggs | None | None | Excreta | -0.0017 | 0.20 | -1.92 | 0.37 | 0.24 | JM2 | 0.4 | 329 | >500^c^ |
| 198 | Pompeo et al. (2016) | *Ascaris* eggs | None | None | Excreta | -0.0005 | 0.19 | -1.78 | 0.21 | 0.25 | JM2 | 0.3 | 329 | >500^c^ |
| 199 | Pompeo et al. (2016) | *Ascaris* eggs | None | None | Excreta | -0.0001 | 0.34 | -0.52 | 0.00 | 0.48 | JM2 | 0.7 | 329 | >500^c^ |
| 200 | Pompeo et al. (2016) | *Ascaris* eggs | None | None | Excreta | -0.0012 | 0.05 | -5.87 | 0.88 | 0.06 | JM2 | 0.2 | 329 | >500^c^ |
| 201 | Pompeo et al. (2016) | *Ascaris* eggs | None | None | Excreta | -0.0024 | 0.33 | -3.05 | 0.62 | 0.45 | JM2 | 0.7 | 329 | >500^c^ |
| 202 | Yin et al. (2016) | Fecal coliforms | None | None | Excreta | -145.33 | NA | NA | NA | NA | NA | 2.0 | 0.028 | 0.027 |
| 203 | Yin et al. (2016) | *Streptococcus* | None | None | Excreta | -169.69 | NA | NA | NA | NA | NA | 2.1 | 0.028 | 0.027 |
| 204 | Yin et al. (2016) | *Salmonella* | None | None | Excreta | -131.77 | NA | NA | NA | NA | NA | 1.6 | 0.028 | 0.035 |
| 205 | Yin et al. (2016) | Fecal coliforms | None | None | Excreta | -128.96 | NA | NA | NA | NA | NA | 2.0 | 0.028 | 0.027 |
| 206 | Yin et al. (2016) | *Streptococcus* | None | None | Excreta | -152.54 | NA | NA | NA | NA | NA | 2.2 | 0.028 | 0.025 |
| 207 | Yin et al. (2016) | *Salmonella* | None | None | Excreta | -142.92 | NA | NA | NA | NA | NA | 1.7 | 0.028 | 0.032 |
| 208 | Yin et al. (2016) | Fecal coliforms | None | None | Excreta | -164.90 | 0.59 | NA | NA | NA | NA | 2.4 | 0.042 | 0.036 |
| 209 | Yin et al. (2016) | *Streptococcus* | None | None | Excreta | -134.05 | 0.38 | NA | NA | NA | NA | 2.7 | 0.042 | 0.030 |
| 210 | Yin et al. (2016) | *Salmonella* | None | None | Excreta | -184.64 | 0.93 | NA | NA | NA | NA | 3.3 | 0.042 | 0.026 |
| 211 | Yin et al. (2016) | Fecal coliforms | None | None | Excreta | -164.45 | 0.59 | NA | NA | NA | NA | 2.4 | 0.042 | 0.035 |
| 212 | Yin et al. (2016) | *Streptococcus* | None | None | Excreta | -134.75 | 0.30 | NA | NA | NA | NA | 2.8 | 0.042 | 0.029 |
| 213 | Yin et al. (2016) | *Salmonella* | None | None | Excreta | -189.18 | 0.89 | NA | NA | NA | NA | 3.4 | 0.042 | 0.025 |
| 214 | Yin et al. (2016) | Fecal coliforms | None | None | Excreta | -237.36 | 1.46 | 237.36 | 237.36 | 6.70 | log-linear | 4.9 | 0.056 | 0.031 |
| 215 | Yin et al. (2016) | *Streptococcus* | None | None | Excreta | -207.90 | 0.68 | 207.90 | 207.90 | 6.04 | log-linear | 4.1 | 0.056 | 0.032 |
| 216 | Yin et al. (2016) | *Salmonella* | None | None | Excreta | -262.47 | 0.65 | NA | NA | NA | NA | 3.4 | 0.042 | 0.029 |
| 217 | Yin et al. (2016) | Fecal coliforms | None | None | Excreta | -224.41 | 3.27 | 224.41 | 224.41 | 6.74 | log-linear | 5.0 | 0.056 | 0.034 |
| 218 | Yin et al. (2016) | *Streptococcus* | None | None | Excreta | -207.70 | 0.68 | 207.70 | 207.70 | 5.94 | log-linear | 4.0 | 0.056 | 0.033 |
| 219 | Yin et al. (2016) | *Salmonella* | None | None | Excreta | -251.61 | 0.90 | NA | NA | NA | NA | 3.3 | 0.042 | 0.029 |
| 220 | Yin et al. (2016) | Fecal coliforms | None | None | Excreta | -220.97 | 3.23 | 220.97 | 220.97 | 6.70 | log-linear | 5.0 | 0.056 | 0.034 |
| 221 | Yin et al. (2016) | *Streptococcus* | None | None | Excreta | -214.15 | 0.83 | 214.15 | 214.15 | 6.08 | log-linear | 4.1 | 0.056 | 0.033 |
| 222 | Yin et al. (2016) | *Salmonella* | None | None | Excreta | -245.13 | 0.97 | NA | NA | NA | NA | 3.4 | 0.042 | 0.028 |
| 223 | Sossou et al. (2014) | Total coliforms | Organic | None | Feces only | -0.1105 | 0.47 | -4.19 | 2.87 | 0.32 | JM2 | 3.3 | 60 | 21 |
| 224 | Sossou et al. (2014) | *E. coli* | Organic | None | Feces only | -0.0846 | 0.15 | -1.90 | 2.04 | 0.40 | log-linear | 3.0 | 60 | 30 |
| 225 | Sossou et al. (2014) | Streptococcus | Organic | None | Feces only | -0.1058 | 0.26 | -2.57 | 2.59 | 0.27 | JM2 | 3.6 | 60 | 16 |
| 226 | Sossou et al. (2014) | Sulfur reducing clostridia | Organic | None | Feces only | -0.1008 | 0.46 | -0.34 | 2.29 | 0.86 | log-linear | 4.3 | 60 | 7.8 |
| 227 | Sossou et al. (2014) | Entamoeba cysts | Organic | None | Feces only | -0.0425 | 0.11 | -5.79 | 1.99 | 0.20 | log-linear | 1.2 | 60 | 109 |
| 228 | Sossou et al. (2014) | Ascaris eggs | Organic | None | Feces only | -0.0719 | 0.37 | -1.88 | 1.27 | 0.38 | log-linear | 1.3 | 30 | 56 |
| 229 | Darimani et al. (2015) | *E. coli* | None | None | Feces only | -16.990 | 1.84 | NA | NA | NA | NA | 1.7 | 0.17 | 0.22 |
| 230 | Darimani et al. (2015) | *E. coli* | None | None | Feces only | -6.3028 | 2.02 | NA | NA | NA | NA | 1.4 | 0.17 | 0.58 |
| 231 | Darimani et al. (2015) | *E. coli* | None | None | Feces only | -6.7983 | 1.95 | NA | NA | NA | NA | 1.4 | 0.17 | 0.55 |
| 232 | Darimani et al. (2015) | *E. coli* | None | None | Feces only | -1.3192 | 1.20 | 2.16 | 0.00 | 0.90 | JM2 | 1.4 | 0.33 | 2.4^d^ |
| 233 | Darimani et al. (2015) | *E. coli* | None | None | Feces only | -21.537 | 0.45 | 10.73 | 3.22 | 0.28 | JM2 | 3.2 | 0.33 | 0.15 |
| 234 | Darimani et al. (2015) | *E. coli* | None | None | Feces only | -32.165 | 0.51 | 15.93 | 4.96 | 0.49 | JM2 | 4.7 | 0.33 | 0.10 |
| 235 | Darimani et al. (2015) | *E. coli* | None | None | Feces only | -1112.5 | 2.10 | NA | NA | NA | NA | 5.7 | 0.012 | 0.0036 |
| 236 | Darimani et al. (2015) | *E. coli* | None | None | Feces only | -1210.0 | 1.19 | NA | NA | NA | NA | 6.5 | 0.013 | 0.0035 |
| 237 | Darimani et al. (2015) | *E. coli* | None | None | Feces only | -1491.5 | NA | NA | NA | NA | NA | 5.2 | 0.0080 | 0.0031 |
| 238 | Darimani et al. (2015) | *Enterococcus* | None | None | Feces only | -0.2805 | 0.21 | -0.97 | 0.20 | 0.16 | JM2 | 0.2 | 0.25 | 16^d^ |
| 239 | Darimani et al. (2015) | *Enterococcus* | None | None | Feces only | -2.0856 | 0.23 | 0.67 | 0.73 | 0.18 | JM2 | 0.3 | 0.25 | 2.2^d^ |
| 240 | Darimani et al. (2015) | *Enterococcus* | None | None | Feces only | -2.8229 | 0.34 | 0.38 | 0.00 | 0.06 | JM2 | 0.4 | 0.25 | 1.5^d^ |
| 241 | Darimani et al. (2015) | *Enterococcus* | None | None | Feces only | -9.9603 | 1.41 | 4.49 | 0.13 | 0.68 | JM2 | 2.4 | 0.33 | 0.29 |
| 242 | Darimani et al. (2015) | *Enterococcus* | None | None | Feces only | -13.396 | 1.62 | 5.67 | 0.07 | 0.43 | JM2 | 2.8 | 0.33 | 0.18 |
| 243 | Darimani et al. (2015) | *Enterococcus* | None | None | Feces only | -20.682 | 1.00 | 9.84 | 2.26 | 0.15 | JM2 | 3.2 | 0.33 | 0.16 |
| 244 | Darimani et al. (2015) | *Enterococcus* | None | None | Feces only | -78.591 | 0.22 | 78.59 | 78.59 | 2.51 | log-linear | 1.9 | 0.054 | 0.061 |
| 245 | Darimani et al. (2015) | *Enterococcus* | None | None | Feces only | -89.524 | 0.86 | 89.52 | 89.52 | 4.11 | log-linear | 2.3 | 0.054 | 0.038 |
| 246 | Darimani et al. (2015) | *Enterococcus* | None | None | Feces only | -136.58 | 0.56 | 136.58 | 136.58 | 5.30 | log-linear | 3.4 | 0.054 | 0.028 |
| 247 | Darimani et al. (2015) | *Enterococcus* | Lime | None | Feces only | -186.60 | 1.87 | NA | NA | NA | NA | 2.5 | 0.029 | 0.019 |
| 248 | Darimani et al. (2015) | *Enterococcus* | Lime | None | Feces only | -82.877 | 0.89 | NA | NA | NA | NA | 1.1 | 0.029 | 0.050 |
| 249 | Darimani et al. (2015) | *Enterococcus* | Lime | None | Feces only | -59.635 | 0.15 | NA | NA | NA | NA | 0.8 | 0.029 | 0.076 |
| 250 | Darimani et al. (2015) | *E. coli* | Lime | None | Feces only | -1978.9 | NA | NA | NA | NA | NA | 7.0 | 0.0080 | 0.0023 |
| 251 | Darimani et al. (2015) | *E. coli* | Lime | None | Feces only | -1862.6 | NA | NA | NA | NA | NA | 6.9 | 0.0085 | 0.0024 |
| 252 | Darimani et al. (2015) | *E. coli* | Lime | None | Feces only | -235.70 | 1.56 | 235.70 | 235.70 | 4.48 | log-linear | 2.3 | 0.021 | 0.013 |
| 253 | Decrey and Kohn (2017) | MS2 coliphage | None | None | Excreta | -1.0044 | 0.82 | -7.19 | 7.99 | 0.90 | JM2 | 7.0 | 17 | 4.4 |
| 254 | Decrey and Kohn (2017) | T4 coliphage | None | None | Excreta | -0.1081 | 0.33 | -10.51 | 4.00 | 0.14 | JM2 | 2.2 | 49 | 44 |
| 255 | Decrey and Kohn (2017) | Adenovirus | None | None | Excreta | -2.1237 | 0.45 | 2.22 | 5.66 | 0.31 | JM2 | 4.9 | 5.0 | 1.5 |
| 256 | Fidjeland et al. (2016) | *Ascaris* | None | Urea | Excreta | -0.0014 | 0.03 | -1.84 | 3.2E-09 | 0.05 | log-linear | 0.1 | 28 | >500^c^ |
| 257 | Fidjeland et al. (2016) | *Ascaris* | None | Urea | Excreta | -0.0014 | 0.01 | NA | NA | NA | NA | 0.1 | 15 | >500^c^ |
| 258 | Fidjeland et al. (2016) | *Ascaris* | None | Urea | Excreta | -0.3682 | 1.02 | -15.72 | 7.79 | 0.81 | JM2 | 2.5 | 17 | 14 |
| 259 | Fidjeland et al. (2016) | *Ascaris* | None | None | Excreta | -0.0009 | 0.01 | -2.20 | 0.10 | 0.01 | JM2 | 0.1 | 51 | >500^c^ |
| 260 | Fidjeland et al. (2016) | *Ascaris* | None | Urea | Excreta | 0.0021 | 0.18 | -1.41 | 0.06 | 0.15 | log-linear | 0.2 | 49 | >500^c^ |
| 261 | Fidjeland et al. (2016) | *Ascaris* | None | Urea | Excreta | -0.0010 | 0.02 | -2.65 | 0.14 | 0.01 | JM2 | 0.1 | 48 | >500^c^ |
| 262 | Fidjeland et al. (2016) | *Ascaris* | None | Urea | Excreta | -0.1561 | 1.02 | -41.35 | 13.26 | 0.64 | JM2 | 2.3 | 35 | 32 |
| 263 | Fidjeland et al. (2016) | *Ascaris* | None | Urea | Excreta | -0.2091 | 1.45 | -45.57 | 16.84 | 0.14 | JM2 | 2.1 | 20 | 20 |
| 264 | Fidjeland et al. (2016) | *Ascaris* | None | Urea | Excreta | -0.1567 | NA | NA | NA | NA | NA | 0.5 | 6.7 | 29 |
| 265 | Fidjeland et al. (2016) | *Ascaris* | None | None | Excreta | -0.0012 | 0.02 | -2.67 | 0.11 | 0.03 | log-linear | 0.1 | 50 | >500^c^ |
| 266 | Fidjeland et al. (2016) | *Ascaris* | None | Urea | Excreta | -0.0362 | 0.38 | NA | NA | NA | NA | 0.9 | 58 | 131 |
| 267 | Fidjeland et al. (2016) | *Ascaris* | None | None | Excreta | -0.0044 | 0.12 | NA | NA | NA | NA | 0.2 | 60 | >500^c^ |
| 268 | Fidjeland et al. (2016) | *Salmonella* spp. | None | Urea | Excreta | -6.9909 | NA | NA | NA | NA | NA | 3.1 | 1.0 | 0.66 |
| 269 | Fidjeland et al. (2016) | *Salmonella* spp. | None | Urea | Excreta | -0.0914 | 0.02 | NA | NA | NA | NA | 0.2 | 4.1 | 51 |
| 270 | Fidjeland et al. (2016) | *Salmonella* spp. | None | Urea | Excreta | -0.0605 | 0.26 | -22.72 | 1.3E-05 | 0.27 | log-linear | 0.0 | 3.0 | 80 |
| 271 | Fidjeland et al. (2016) | *Salmonella* spp. | None | Urea | Excreta | -11.460 | NA | NA | NA | NA | NA | 5.1 | 1.0 | 0.40 |
| 272 | Fidjeland et al. (2016) | *Salmonella* spp. | None | Urea | Excreta | -5.5948 | NA | NA | NA | NA | NA | 2.5 | 1.0 | 0.82 |
| 273 | Fidjeland et al. (2016) | *Salmonella* spp. | None | Urea | Excreta | -3.3940 | NA | NA | NA | NA | NA | 3.0 | 2.0 | 1.4 |
| 274 | Fidjeland et al. (2016) | *Salmonella* spp. | None | Urea | Excreta | -0.3584 | 0.43 | NA | NA | NA | NA | 0.6 | 4.1 | 13 |
| 275 | Fidjeland et al. (2016) | *Salmonella* spp. | None | Urea | Excreta | 0.2454 | 0.07 | -5.01 | 3.4E-12 | 0.56 | log-linear | 0.0 | 4.1 | NA^e^ |
| 276 | Fidjeland et al. (2016) | *Salmonella* spp. | None | None | Excreta | 0.3608 | 0.17 | -5.01 | 2.5E-11 | 1.02 | log-linear | 0.0 | 4.1 | NA^e^ |
| 277 | Fidjeland et al. (2016) | *Salmonella* spp. | None | Urea | Excreta | -9.5380 | NA | NA | NA | NA | NA | 4.1 | 1.0 | 0.48 |
| 278 | Fidjeland et al. (2016) | *Salmonella* spp. | None | Urea | Excreta | -7.7083 | NA | NA | NA | NA | NA | 1.8 | 0.53 | 0.60 |
| 279 | Fidjeland et al. (2016) | *Salmonella* spp. | None | None | Excreta | -0.2211 | 0.36 | 0.47 | 0.17 | 0.31 | JM2 | 0.6 | 3.8 | 19^d^ |
| 280 | Tonner-Klank et al. (2007) | *S. typhimurium* phage 28B | None | None | Feces only | -0.0408 | 2.34 | -16.30 | 4.62 | 2.90 | JM2 | 5.5 | 217 | 92 |
| 281 | Tonner-Klank et al. (2007) | Thermotolerant coliforms | None | None | Feces only | -0.0565 | 3.12 | -6.27 | 4.19 | 2.88 | JM2 | 7.0 | 217 | 13 |
| 282 | Tonner-Klank et al. (2007) | Enterococci | None | None | Feces only | -0.0281 | 1.88 | -11.01 | 3.12 | 2.45 | JM2 | 3.5 | 217 | 148 |
| 283 | Tonner-Klank et al. (2007) | *S. senftenberg* | None | None | Feces only | -0.2069 | 3.02 | -19.78 | 8.73 | 1.37 | JM2 | 8.0 | 93 | 16 |
| 284 | Tonner-Klank et al. (2007) | *S. typhimurium* phage 28B | None | None | Feces only | -0.0357 | 3.15 | -8.00 | 3.37 | 3.72 | JM2 | 6.9 | 216 | 42 |
| 285 | Tonner-Klank et al. (2007) | *S. senftenberg* | None | None | Feces only | -0.2130 | 2.95 | -29.51 | 10.91 | 1.72 | JM2 | 7.9 | 93 | 23 |
| 286 | Tonner-Klank et al. (2007) | Thermotolerant coliforms | None | None | Feces only | -0.1881 | 1.82 | -34.07 | 11.27 | 0.88 | JM2 | 6.9 | 92 | 31 |
| 287 | Tonner-Klank et al. (2007) | Enterococci | None | None | Feces only | -0.0305 | 2.65 | -6.34 | 2.82 | 3.08 | JM2 | 6.0 | 216 | 48 |
| 288 | Odey et al. (2018) | Fecal coliforms | None | None | Excreta | 0.0045 | 0.14 | -5.00 | 1.1E-13 | 0.19 | log-linear | 0.0 | 20 | NA^e^ |
| 289 | Odey et al. (2018) | Fecal coliforms | Organic | None | Excreta | -0.0114 | 0.04 | -7.15 | 1.89 | 0.04 | JM2 | 0.1 | 20 | >40^c^ |
| 290 | Odey et al. (2018) | Fecal coliforms | None | None | Excreta | -0.2305 | 0.15 | NA | NA | NA | NA | 1.0 | 10 | 20 |
| 291 | Odey et al. (2018) | Fecal coliforms | Organic | None | Excreta | 0.0213 | 1.81 | NA | NA | NA | NA | 0.0 | 10 | NA^e^ |
| 292 | Niwagaba et al. (2009) | *E. coli* | Ash | None | Feces only | -0.0623 | 0.37 | 0.24 | 1.58 | 0.51 | JM2 | 3.0 | 81 | 16 |
| 293 | Niwagaba et al. (2009) | Enterococci | Ash | None | Feces only | -0.0197 | 0.45 | 0.86 | 0.99 | 0.94 | JM2 | 2.6 | 101 | 44 |
| 294 | Niwagaba et al. (2009) | Enterococci | Organic | None | Feces only | -0.0556 | 0.40 | -2.21 | 1.30 | 0.98 | JM2 | 1.9 | 57 | 188 |
| 295 | Niwagaba et al. (2009) | *E. coli* | Organic | None | Feces only | -0.1271 | 0.17 | -5.42 | 3.00 | 0.46 | log-linear | 3.0 | 57 | 32 |
| 296 | Niwagaba et al. (2009) | Enterococci | Ash | None | Feces only | -0.2106 | 0.48 | -0.71 | 2.27 | 0.31 | JM2 | 3.1 | 31 | 10 |
| 297 | Niwagaba et al. (2009) | *E. coli* | Ash | None | Feces only | -0.0675 | 0.25 | -0.51 | 0.90 | 0.31 | JM2 | 1.0 | 30 | 283 |
| 298 | Niwagaba et al. (2009) | Enterococci | Organic | None | Feces only | -0.1133 | 0.54 | 0.86 | 1.33 | 0.66 | JM2 | 2.3 | 30 | 17 |
| 299 | Niwagaba et al. (2009) | *E. coli* | Organic | None | Feces only | -0.1162 | 0.42 | -0.15 | 1.35 | 0.47 | JM2 | 1.7 | 30 | 34 |
| 300 | Anderson et al. (2015) | *E. coli* | None | None | Excreta | -0.3718 | 0.56 | NA | NA | NA | NA | 1.7 | 7.0 | 8.4 |
| 302 | Anderson et al. (2015) | *E. coli* | None | None | Excreta | 0.0137 | 0.27 | -3.13 | 2.4E-13 | 0.27 | JM2 | 0.2 | 7.0 | NA^e^ |
| 303 | Anderson et al. (2015) | *E. coli* | None | Urea | Excreta | -1.9046 | 2.17 | NA | NA | NA | NA | 2.8 | 3.0 | 2.0 |
| 304 | Anderson et al. (2015) | Fecal coliforms | None | None | Excreta | 0.0679 | 0.32 | -5.01 | 2.2E-14 | 0.44 | log-linear | 0.1 | 7.0 | NA^e^ |
| 305 | Anderson et al. (2015) | Fecal coliforms | None | Urea | Excreta | -1.3335 | 0.83 | 3.58 | 1.53 | 0.27 | JM2 | 2.5 | 4.0 | 1.9 |
| 306 | Anderson et al. (2015) | Fecal coliforms | None | None | Excreta | -0.5616 | 0.14 | NA | NA | NA | NA | 2.5 | 7.0 | 4.8 |
| 307 | Anderson et al. (2015) | Fecal coliforms | None | Urea | Excreta | -0.6332 | NA | NA | NA | NA | NA | 1.9 | 4.0 | 4.4 |
| 308 | Anderson et al. (2015) | *E. coli* | None | None | Excreta | -0.9473 | 0.12 | 1.00 | 10.00 | 0.20 | log-linear | 0.1 | 0.21 | 4.8 |
| 309 | Anderson et al. (2015) | *E. coli* | Lime | None | Excreta | -6.4736 | 0.11 | 6.35 | 3.73 | 0.23 | log-linear | 0.4 | 0.21 | 0.77 |
| 310 | Anderson et al. (2015) | *E. coli* | Lime | None | Excreta | -27.078 | 1.09 | 27.08 | 27.08 | 3.82 | log-linear | 2.5 | 0.21 | 0.14 |
| 311 | Anderson et al. (2015) | *E. coli* | Lime | None | Excreta | -17.368 | 1.79 | 17.37 | 17.37 | 4.59 | log-linear | 2.3 | 0.21 | 0.12 |
| 312 | Anderson et al. (2015) | *E. coli* | Lime | None | Excreta | -17.368 | 1.79 | 17.37 | 17.37 | 5.63 | log-linear | 2.8 | 0.21 | 0.050 |
| 313 | Anderson et al. (2015) | *E. coli* | Lime | None | Excreta | -20.526 | 2.11 | 20.53 | 20.53 | 7.70 | log-linear | 3.8 | 0.21 | 0.46 |
| 314 | Anderson et al. (2015) | Total coliforms | None | None | Excreta | -1.8158 | 0.17 | 39.55 | 25.64 | 0.12 | JM2 | 0.2 | 0.21 | 0.26 |
| 315 | Anderson et al. (2015) | Total coliforms | Lime | None | Excreta | -8.4472 | 0.04 | -4.41 | 3.3E-11 | 0.77 | log-linear | 0.6 | 0.21 | 0.58 |
| 316 | Anderson et al. (2015) | Total coliforms | Lime | None | Excreta | -24.868 | 1.13 | 24.87 | 24.87 | 3.16 | log-linear | 2.0 | 0.21 | 0.18 |
| 317 | Anderson et al. (2015) | Total coliforms | Lime | None | Excreta | -10.026 | 0.55 | 3.36 | 0.54 | 0.27 | JM2 | 1.2 | 0.21 | 10 |
| 318 | Anderson et al. (2015) | Total coliforms | Lime | None | Excreta | -23.289 | 1.33 | 23.29 | 23.29 | 5.32 | log-linear | 3.0 | 0.21 | 0.073 |
| 319 | Anderson et al. (2015) | Total coliforms | Lime | None | Excreta | -23.289 | 2.40 | 23.29 | 23.29 | 8.07 | log-linear | 4.0 | 0.21 | 0.45 |
| 320 | Nordin et al. (2009b) | *Salmonella* spp. | None | None | Feces only | -0.3933 | 1.71 | 1.27 | 3.58 | 2.25 | JM2 | 5.7 | 27 | 2.5 |
| 321 | Nordin et al. (2009b) | *Salmonella* spp. | None | Urea | Feces only | -2.3024 | NA | NA | NA | NA | NA | 4.7 | 3.7 | 1.0 |
| 322 | Nordin et al. (2009b) | *Salmonella* spp. | None | Urea | Feces only | -5.1905 | NA | NA | NA | NA | NA | 5.5 | 2.0 | 0.42 |
| 323 | Hashemi et al. (2019) | *E. coli* | Organic | None | Feces only | -0.0876 | 0.81 | 0.89 | 1.41 | 0.31 | JM2 | 2.8 | 56 | 14 |
| 324 | Hashemi et al. (2019) | *E. coli* | Organic | None | Feces only | -0.0934 | 0.78 | 1.10 | 1.44 | 0.15 | JM2 | 3.0 | 56 | 11 |
| 325 | Hashemi et al. (2019) | *E. coli* | Organic | None | Feces only | -0.2191 | 1.45 | 2.57 | 1.83 | 0.16 | JM2 | 3.9 | 35 | 3.0 |
| 326 | Nakamura and Taylor (1965) | *Shigella sonnei* | None | None | Feces only | -0.1522 | 2.27 | -86.14 | 23.53 | 0.71 | JM2 | 4.2 | 59 | 47 |
| 327 | Nakamura and Taylor (1965) | *Shigella sonnei* | None | None | Feces only | -0.2592 | 1.05 | -20.73 | 8.12 | 0.17 | JM2 | 4.1 | 42 | 23 |
| 328 | Nakamura and Taylor (1965) | *Shigella flexneri* | None | None | Feces only | -0.2827 | 0.93 | NA | NA | NA | NA | 3.9 | 34 | 19 |
| 329 | Nakamura and Taylor (1965) | *Shigella sonnei* | None | None | Feces only | -0.0122 | 0.49 | 2.13 | 0.45 | 0.69 | JM2 | 2.2 | 118 | 246 |
| 330 | Nakamura and Taylor (1965) | *Shigella sonnei* | None | None | Feces only | -0.0919 | 1.79 | 1.62 | 1.35 | 1.57 | JM2 | 3.9 | 58 | 9.0 |
| 331 | Nakamura and Taylor (1965) | *Shigella flexneri* | None | None | Feces only | -0.0869 | 0.85 | 1.78 | 1.56 | 0.82 | JM2 | 3.9 | 62 | 6.1 |
| 332 | Moe and Shirley (1982) | Rotavirus | None | None | Feces only | -0.0778 | 0.20 | -6.17 | 2.47 | 0.34 | log-linear | 1.6 | 45 | 60 |
| 333 | Moe and Shirley (1982) | Rotavirus | None | None | Feces only | -0.3036 | 0.22 | -2.87 | 2.54 | 0.42 | log-linear | 1.9 | 13 | 15 |
| 334 | Moe and Shirley (1982) | Rotavirus | None | None | Feces only | -0.8068 | 0.07 | 0.55 | 2.00 | 0.20 | log-linear | 1.6 | 4.3 | 5.4 |
| 335 | Moe and Shirley (1982) | Rotavirus | None | None | Feces only | -0.4383 | 0.21 | -0.92 | 2.39 | 0.26 | JM2 | 2.2 | 11 | 10 |
| 336 | Moe and Shirley (1982) | Rotavirus | None | None | Feces only | -0.8377 | 0.35 | -3.38 | 4.92 | 0.93 | log-linear | 4.3 | 11 | 5.6 |
| 337 | Moe and Shirley (1982) | Rotavirus | None | None | Feces only | -1.0350 | 0.10 | -2.03 | 4.90 | 0.55 | log-linear | 4.1 | 9.0 | 4.3 |
| 338 | Moe and Shirley (1982) | Rotavirus | None | None | Feces only | -0.3933 | 0.13 | -1.35 | 2.32 | 0.21 | log-linear | 1.9 | 11 | 11 |
| 339 | Moe and Shirley (1982) | Rotavirus | None | None | Feces only | -0.3043 | 0.21 | -3.34 | 2.69 | 0.38 | log-linear | 1.9 | 13 | 16 |
| 340 | Sossou et al. (2016b) | *E. coli* | None | None | Feces only | -0.4498 | 0.12 | -1.66 | 2.53 | 0.12 | JM2 | 0.7 | 3.0 | 12 |
| 341 | Sossou et al. (2016b) | *E. coli* | None | Urea | Feces only | -0.8155 | 0.09 | -0.17 | 2.21 | 0.07 | JM2 | 1.0 | 3.0 | 8.7 |
| 342 | Sossou et al. (2016b) | *E. coli* | None | Urea | Feces only | -1.1451 | 0.12 | 0.77 | 2.27 | 0.23 | log-linear | 1.6 | 3.0 | 4.0 |
| 343 | Sossou et al. (2016b) | *E. coli* | None | None | Feces only | -0.7184 | 0.11 | -0.79 | 2.45 | 0.10 | JM2 | 0.9 | 3.0 | 9.0 |
| 344 | Sossou et al. (2016b) | *E. coli* | None | Urea | Feces only | -1.0521 | 0.10 | 0.37 | 2.35 | 0.16 | log-linear | 1.4 | 3.0 | 4.4 |
| 345 | Sossou et al. (2016b) | *Enterococcus faecalis* | None | None | Feces only | -0.2984 | 0.04 | -0.95 | 1.27 | 0.05 | log-linear | 0.4 | 3.0 | 15 |
| 346 | Sossou et al. (2016b) | *Enterococcus faecalis* | None | Urea | Feces only | -0.6822 | 0.09 | -0.37 | 2.06 | 0.13 | log-linear | 1.0 | 3.0 | 6.8 |
| 347 | Sossou et al. (2016b) | *Enterococcus faecalis* | None | Urea | Feces only | -0.9977 | 0.14 | -0.08 | 2.67 | 0.20 | log-linear | 1.4 | 3.0 | 4.7 |
| 348 | Sossou et al. (2016b) | *Enterococcus faecalis* | None | None | Feces only | -0.9313 | 0.22 | -0.67 | 3.05 | 0.18 | JM2 | 1.3 | 3.0 | 5.6 |
| 349 | Sossou et al. (2016b) | *Enterococcus faecalis* | None | Urea | Feces only | -0.5709 | 0.10 | -0.91 | 2.20 | 0.10 | JM2 | 0.8 | 3.0 | 12 |
| 350 | Sossou et al. (2016b) | *Ascaris lumbricoides* | None | None | Feces only | -0.1179 | 0.02 | -1.65 | 0.85 | 0.04 | log-linear | 0.2 | 3.0 | 39 |
| 351 | Sossou et al. (2016b) | *Ascaris lumbricoides* | None | Urea | Feces only | -0.4749 | 0.11 | -1.58 | 2.50 | 0.08 | JM2 | 0.7 | 3.0 | 12 |
| 352 | Sossou et al. (2016b) | *Ascaris lumbricoides* | None | Urea | Feces only | -0.7658 | 0.21 | -1.66 | 3.57 | 0.16 | JM2 | 1.1 | 3.0 | 5.8 |
| 353 | Sossou et al. (2016b) | *Ascaris lumbricoides* | None | None | Feces only | -0.6553 | 0.18 | -1.75 | 3.30 | 0.15 | JM2 | 0.9 | 3.0 | 6.8 |
| 354 | Sossou et al. (2016b) | *Ascaris lumbricoides* | None | Urea | Feces only | -0.3118 | 0.09 | -1.71 | 2.05 | 0.06 | JM2 | 0.5 | 3.0 | 22 |
| 355 | Berendes et al. (2015) | *E. coli* | Organic | None | Feces only | -0.8866 | 2.22 | -8.84 | 7.49 | 2.57 | JM2 | 4.9 | 14 | 6.0 |
| 356 | Berendes et al. (2015) | *E. coli* | Organic | None | Feces only | -0.7784 | 1.30 | 1.14 | 3.67 | 1.41 | JM2 | 4.8 | 14 | 2.6 |
| 357 | Berendes et al. (2015) | *E. coli* | Organic | None | Feces only | -0.5034 | 2.29 | 8.29 | 1.08 | 1.04 | JM2 | 4.9 | 14 | 0.032 |
| 358 | Berendes et al. (2015) | *E. coli* | Organic | None | Feces only | -0.3945 | 2.52 | 8.06 | 0.45 | 2.01 | JM2 | 4.9 | 14 | 0.00047 |
| 359 | Berendes et al. (2015) | *E. coli* | Organic | None | Feces only | -0.2153 | 0.57 | -12.99 | 6.09 | 0.04 | JM2 | 4.8 | 52 | 18 |
| 360 | Berendes et al. (2015) | *E. coli* | Organic | None | Feces only | -0.1822 | 2.80 | 6.12 | 0.94 | 1.17 | JM2 | 4.9 | 52 | 0.20 |
| 361 | Berendes et al. (2015) | *E. coli* | Organic | None | Feces only | -0.2772 | 1.68 | NA | NA | NA | NA | 4.8 | 40 | 19 |
| 362 | Berendes et al. (2015) | *E. coli* | Organic | None | Feces only | -0.5572 | NA | NA | NA | NA | NA | 4.8 | 20 | 8.3 |
| 363 | Graham et al. (2003) | *Cryptosporidium* | Sawdust | None | Excreta | -0.0024 | 0.18 | NA | NA | NA | NA | 0.3 | 180 | >500^c^ |
| 364 | Graham et al. (2003) | *Cryptosporidium* | Lime and soil | None | Feces only | -0.0149 | 0.25 | NA | NA | NA | NA | 1.1 | 180 | 321 |
| 365 | Graham et al. (2003) | *Giardia* | Sawdust | None | Excreta | -0.0015 | 0.11 | NA | NA | NA | NA | 0.1 | 180 | >500^c^ |
| 366 | Graham et al. (2003) | *Giardia* | Lime and soil | None | Feces only | -0.0040 | 0.09 | NA | NA | NA | NA | 0.2 | 180 | >500^c^ |
| 367 | Graham et al. (2003) | Fecal coliforms | Sawdust | None | Excreta | -0.0345 | 0.00 | NA | NA | NA | NA | 2.5 | 180 | 147 |
| 368 | Graham et al. (2003) | Fecal coliforms | Lime and soil | None | Feces only | -0.0288 | 0.14 | NA | NA | NA | NA | 2.5 | 180 | 141 |
| 369 | Robertson et al. (1992) | *Cryptosporidium* | None | None | Feces only | -0.0151 | 0.09 | -13.65 | 3.13 | 0.03 | JM2 | 1.1 | 178 | 341 |
| 370 | Robertson et al. (1992) | *Cryptosporidium* | None | None | Feces only | -0.0018 | 0.01 | -5.94 | 0.95 | 0.03 | JM2 | 0.1 | 178 | >500^c^ |
| 371 | Robertson et al. (1992) | *Cryptosporidium* | None | None | Feces only | -0.0211 | 0.15 | -13.36 | 3.33 | 0.21 | JM2 | 1.7 | 178 | 221 |

^a^ According to the best fit model (*i.e.*, with the lowest AIC value) or if the JM2 model could not be fit, then according to the log-linear model

^b^ JM2 model fitting was not possible due to small sample size or estimation of T_99_ using the JM2 model was not possible due to excessive extrapolation; T_99_ was estimated based on the log-linear model

^c^ Experimental decay was insufficient to extrapolate a T_99_ value beyond 500 days

^d^ Estimation of T_99_ from the JM2 model was not practical due to large extrapolation; T_99_ value shown is based on the log-linear model

^e^ T_99_ value could not be estimated due to limited experimental data

^f^ Excreta means feces and urine

**Full R Markdown Code**

The R Markdown file used to generate this printout can be found in the following repository on GitHub: <https://github.com/mverbyla/pathogen.decay.in.pits>. It reproduces the figures and tables from the manuscript and from this Supplemental Information document.

persistencePits v7.0

Matthew E. Verbyla

6/16/2022

## Load packages

First, load the required packages.

library(ggplot2)
library(gridExtra)
library("PerformanceAnalytics")
library("Hmisc")
library("knitr")
library("bbmle")

## Load and explore data

Now, we will load and explore the persistence data, which are stored on the GWPP K2P Data Portal under the following dataset: <http://data.waterpathogens.org/dataset/persistence-treatment>.

The next chunk of code explores the data set to quantify the total number of data points, experiments, and the breakdown of lab-based studies, field-based studies, and the number of experiments done with different types of pathogen groups.

nrow(persist) # total number of data points

## [1] 1382

length(unique(persist$experiment_id)) # total number of experiments

## [1] 243

length(persist[persist$location_comment=="Laboratory-based study",]$experiment_id) # number of data points from lab-based studies

## [1] 1078

length(unique(persist[persist$location_comment=="Laboratory-based study",]$experiment_id)) # number of lab-based experiments

## [1] 190

length(persist[persist$location_comment!="Laboratory-based study",]$experiment_id) # number of data points from field-based studies

## [1] 304

length(unique(persist[persist$location_comment!="Laboratory-based study",]$experiment_id)) # number of field-based experiments

## [1] 53

length(unique(persist[persist$microbial_group=="Virus",]$experiment_id)) # number of experiments with viruses

## [1] 32

length(unique(persist[persist$microbial_group=="Bacteria",]$experiment_id)) # number of experiments with bacteria

## [1] 147

length(unique(persist[persist$microbial_group=="Protozoa",]$experiment_id)) # number of experiments with protozoa

## [1] 8

length(unique(persist[persist$microbial_group=="Helminth",]$experiment_id)) # number of experiments with helminths

## [1] 56

unique(persist[persist$microbial_group=="Virus",]$microorganism_common_name) # types of viruses studied

## [1] "Bacteriophage φX" "Bacteriophage MS2"
## [3] "Salmonella typhimurium phages" "MS2 coliphage"
## [5] "PhiX174 coliphage" "T4 coliphage"
## [7] "Adenovirus" "Salmonella typhimurium phage 28B"
## [9] "Rotavirus"

unique(persist[persist$microbial_group=="Bacteria",]$microorganism_common_name) # types of bacteria studied

## [1] "Fecal Coliforms" "Enterococcus faecalis"
## [3] "Salmonella enterica Typhimurium" "Escherichia Coli"
## [5] "Fecal coliforms" "Streptococcus"
## [7] "Salmonella" "Total coliform"
## [9] "Eschericia Coli" "Sulfur reducing clostridia"
## [11] "Escherichia coli" "Enterococcus"
## [13] "Salmonella spp" "Thermotolerant coliform"
## [15] "Enterococci" "Salmonella senftenberg"
## [17] "Total coliforms" "Salmonella spp."
## [19] "Shigella sonnei" "Shigella flexneri"
## [21] "Escherichia coli "

unique(persist[persist$microbial_group=="Protozoa",]$microorganism_common_name) # types of protozoa studied

## [1] "Entamoeba cysts" "Cryptosporidium" "Giardia"

unique(persist[persist$microbial_group=="Helminth",]$microorganism_common_name) # types of helminths studied

## [1] "Ascaris" "Ascaris suum" "Ascaris eggs"
## [4] "Ascaris ova" "Ascaris lumbricoides"

## Calculate the decay rate coefficients

The next chunk of code sets up some variables that will be used in an upcoming "FOR" loop, which will be used to calculate the decay rate coefficients and other statistics associated with the fit of the log-linear and JM2 models.

N<-length(unique(persist$experiment_id)) # the total number of experiments
M<-length(persist$experiment_id) # the total number of data points
persist$ind<-NA
for(i in 1:M){
 # this creates a unique index variable to distinguish each independent experiment
 persist$ind[i]<-which(data.frame(unique(persist$experiment_id))==persist$experiment_id[i])
}
# here we create lots of NULL variables, which later we will populate values for in the upcoming "for" loop
k<-NULL;k1<-NULL;k2<-NULL;intrcpt<-NULL;group<-NULL;addit<-NULL;urine<-NULL;urea<-NULL;moist<-NULL;temp<-NULL;pH<-NULL;r2<-NULL;t99<-NULL;t99jm<-NULL;authors<-NULL;num<-NULL;aicLL<-NULL;aicJM<-NULL;experiment<-NULL;maxLRV<-NULL;maxTime<-NULL;mcn<-NULL;best<-NULL;sterr<-NULL;sigme<-NULL
par(mfrow=c(3,6)) #this creates the panel layout for the plots used for creating Figure S1

for(i in 1:N){ #in this loop, we calculate the decay rate coefficients for the log-linear and JM2 models
 time<-persist[persist$ind==i,]$time_days # get the time for the present experiment
 lnrv<-persist[persist$ind==i,]$ln_removal # get the ln reduction for the present experiment
 log10_reduction<-persist[persist$ind==i,]$log10_reduction # get the log10 reduction for the present experiment
 # since we calculated the ln reduction, then equation gets algebraically rearranged like this:
 # Ct = Co*exp(-k*t)
 # ln(Ct/Co) = -k*t

 # first, we fit the model like this to express the decay rate coefficient, k, on a LN scale
 fit<-lm(lnrv~time) # lnrv = ln(Ct/Co), so our linear model is lnrv~time
 # then, we fit the model like this to create plots and calculate T99 values more easily later on
 fit2<-lm(log10_reduction~time)

 # next, we set up the JM2 model
 JM2<-function(k1,k2,sigma){
 R = lnrv - log(1/(1+exp(k1+k2*log(time)))) # this is the equation for the JM2 model
 R = suppressWarnings(dnorm(R,0,sigma,log=T)) # this is the standard deviation of the normally distributed difference between measured and modeled LRVs
 -sum(R) # this is the negative log likelihood, which must be maximized (using the MLE method)
 }

 # in order to use MLE to find the best values for the JM2 model, we have to provide some halfway decent guesses for k1, k2, and sigma
 # through trial and error, we found that starting guesses of k1=-5, k2=1, and sigma=3 generally produced converging results for most experiments; the following are exceptions
 if(fit$coefficients[2]<=-10){k1guess=-fit$coefficients[2];k2guess=-fit$coefficients[2];siguess=3}else{
 if(fit$coefficients[2]>=-0.0001){k1guess=-5;k2guess=0.01;siguess=2}else{
 if(i==48|i==181|i==186){k1guess=-0.1;k2guess=1;siguess=1}else{
 if(i==115){k1guess=4;k2guess=100;siguess=1}else{
 if(i==112|i==113|i==105|i==114){k1guess=5;k2guess=1;siguess=1}else{
 if(i==180){k1guess=1;k2guess=10;siguess=1}else{
 k1guess=-5;k2guess=1;siguess=3
 }
 }
 }
 }
 }
 }

 if(length(time)>3){ # it only calculates the k1, k2, and sigma values for the JM2 model if there are more than 3 time points in the experiment
 fit.jm<-mle2(minuslogl=JM2,start=list(k1=k1guess,k2=k2guess,sigma=siguess),optimizer="nlminb") # uses unbounded optimization using portable Fortran programs for numerical computation (PORT) routines
 # Reference: Fox et al. (1978). “The PORT Mathematical Subroutine Library.” ACM Transactions on Mathematical Software, 4(2), 104–126. doi:10.1145/355780.355783
 }else{fit.jm<-NA}

 ## NEXT WE STORE THE VALUES OF THE VARIABLES AND STATISTICS WE WANT TO STORE FOR EACH EXPERIMENT
 # here, we store the basic information about the experiment (e.g., the design, operational, and environmental parameters)
 authors[i]<-as.character(unique(persist[persist$ind==i,]$authors)) # the names of the authors of the paper where the experiment's data were published
 experiment[i]<-as.numeric(unique(persist[persist$ind==i,]$experiment_id)) # the unique experiment ID
 num[i]<-length(time) # number of time points in the experiment
 maxTime[i]<-max(time) # the maximum time point for which data were collected in the experiment
 maxLRV[i]<-as.numeric(max(persist[persist$ind==i,]$log10_reduction)) # the maximum log10 reduction value observed in the experiment
 group[i]<-as.character(unique(persist[persist$ind==i,]$microbial_group)) # the microbial group used in the experiment (viruses, bacteria, protozoa, helminths)
 mcn[i]<-as.character(persist[persist$ind==i,]$microorganism_common_name) # a more specific description of the microbial group
 addit[i]<-as.character(unique(persist[persist$ind==i,]$additive)) # the type of additive applied to the fecal sludge
 urine[i]<-as.character(unique(persist[persist$ind==i,]$urine)) # whether the experiment was done with feces only or feces mixed with urine (excreta)
 moist[i]<-as.numeric(median(persist[persist$ind==i,]$moisture_content_percent)) # the average percent moisture content of the fecal sludge during the experiment
 temp[i]<-as.numeric(median(persist[persist$ind==i,]$temperature_celsius)) # the average temperature of the fecal sludge during the experiment
 pH[i]<-as.numeric(median(persist[persist$ind==i,]$pH)) # the average pH of the fecal sludge during the experiment
 urea[i]<-as.character(unique(persist[persist$ind==i,]$urea)) # whether or not urea (or stored urine) was added to the fecal sludge during the experiment

 # here, we store the statistics for the fit to the log-linear model
 k[i]<-fit$coefficients[2] # the log-linear decay rate coefficient
 intrcpt[i]<-fit$coefficients[1] # the log-linear model Y-intercept
 out<-summary(fit); sterr[i]<-out$coefficients[1,2] # this is the standard error of the decay rate coefficient
 t99[i]<-(2-fit2$coefficients[1])/fit2$coefficients[2] # the predicted T99 value for the log-linear model
 r2[i]<-summary(fit)$r.squared # coefficient of determination for the log-linear model fit
 aicLL[i]<-AIC(fit) # the AIC value for the log-linear model fit

 # and here, we store the statistics for the fit to the JM2 model
 if(!is.na(fit.jm)){k1[i]<-coef(fit.jm)["k1"]} # the 1st JM2 model decay rate coefficient
 if(!is.na(fit.jm)){k2[i]<-coef(fit.jm)["k2"]} # the 2nd JM2 model decay rate coefficient
 if(!is.na(fit.jm)){sigme[i]<-coef(fit.jm)["sigma"]} # the sigma value for the fit of the JM2 model (measure of uncertainty)
 t99jm[i]<-exp((log(99)-k1[i])/k2[i]) # the predicted T99 value for the JM2 model
 if(length(time)>3){aicJM[i]<-AIC(fit.jm)}else{aicJM[i]<-NA} # the AIC value for the JM2 model fit

 # this stores text describing which of the two models had the better fit (Chick Log-Linear Model or JM2 Model), based on the AIC values calculated
 if(is.na(aicJM[i])){best[i]<-"Chick Model"}else{if(aicLL[i]<aicJM[i]){best[i]<-"Chick Model"}else{best[i]<-"JM2 Model"}}

 # this next chunk of script creates the plot for each panel of Figure S1 (new plot created for each experiment during each round of the FOR loop)
 par(mar=c(5,4,3.5,1)) # sets the margins so that the plot looks nice
 plot(time,-log10_reduction,ylim=c(-8,0),xlim=c(0,500),ylab="Log10 Reduction",xlab="Time (days)",main=paste(experiment[i],", ",group[i],", N=",num[i],", LRVmax=",round(maxLRV[i],2),"\n",addit[i],", ",urea[i],", ",round(r2[i],2),"\n",authors[i],sep=""),sub=best[i],cex.main=0.95)
 lines(seq(0,360),-fit2$coefficients[2]*seq(0,360)-fit2$coefficients[1],col="blue")
 if(length(time)>3){lines(seq(0,360),log10(1/(1+exp(coef(fit.jm)["k1"]+coef(fit.jm)["k2"]*log(seq(0,360))))),lty=2,col="red")}
}

# now we will create a data frame with all the values from all experiments
kPit<-data.frame(experiment=experiment,authors=authors,microbial_group=group,mcn=mcn,k=k,se.k=sterr,k1=k1,k2=k2,se.jm=sigme,int=intrcpt,aicLL=aicLL,aicJM=aicJM,num=num,additive=addit,urea=urea,urine=urine,moisture=moist,temp=temp,pH=pH,r2=r2,maxLRV=maxLRV,t99=t99,t99jm=t99jm,bestModel=best)
kPit$k<-round(kPit$k,4)
kPit$k1<-round(kPit$k1,2)
kPit$k2<-round(kPit$k2,2)
kPit.demo<-kPit[c(1,17,126,236),c("experiment","authors","mcn","k","k1","k2")]
kPit.demo

## experiment authors mcn k
## 1 128 Endale et al.(2012) Ascaris -0.0656
## 17 144 Magri et al.(2013) Salmonella enterica Typhimurium -0.0610
## 126 253 Decrey and Kohn (2017) MS2 coliphage -1.0044
## 236 364 Graham et al. (2003) Cryptosporidium -0.0149
## k1 k2
## 1 -6.79 2.48
## 17 -7.38 3.41
## 126 -7.19 7.99
## 236 NA NA

The previous chunk of code hid the plots that were created for Figure S1. Here is a demonstrative plot for experiment 144.


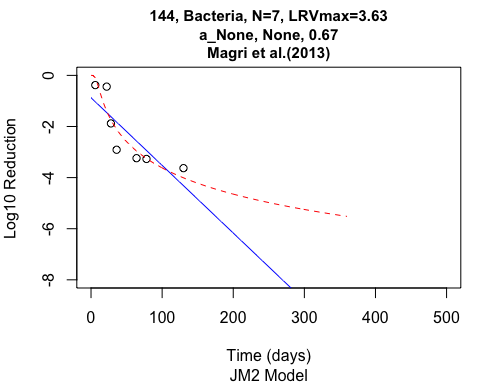


Here is a summary of which model provided the better fit for each experiment

kPit[,c("experiment","mcn","bestModel")]

## experiment mcn bestModel
## 1 128 Ascaris JM2 Model
## 2 129 Ascaris JM2 Model
## 3 130 Ascaris JM2 Model
## 4 131 Ascaris JM2 Model
## 5 132 Fecal Coliforms Chick Model
## 6 133 Fecal Coliforms Chick Model
## 7 134 Fecal Coliforms JM2 Model
## 8 135 Fecal Coliforms JM2 Model
## 9 136 Enterococcus faecalis Chick Model
## 10 137 Enterococcus faecalis Chick Model
## 11 138 Enterococcus faecalis Chick Model
## 12 139 Enterococcus faecalis Chick Model
## 13 140 Enterococcus faecalis Chick Model
## 14 141 Salmonella enterica Typhimurium JM2 Model
## 15 142 Salmonella enterica Typhimurium JM2 Model
## 16 143 Salmonella enterica Typhimurium JM2 Model
## 17 144 Salmonella enterica Typhimurium JM2 Model
## 18 145 Salmonella enterica Typhimurium JM2 Model
## 19 146 Bacteriophage φX JM2 Model
## 20 147 Bacteriophage φX Chick Model
## 21 148 Bacteriophage φX JM2 Model
## 22 149 Bacteriophage φX Chick Model
## 23 150 Bacteriophage φX Chick Model
## 24 151 Bacteriophage φX Chick Model
## 25 152 Bacteriophage MS2 JM2 Model
## 26 153 Bacteriophage MS2 JM2 Model
## 27 154 Bacteriophage MS2 JM2 Model
## 28 155 Bacteriophage MS2 JM2 Model
## 29 156 Bacteriophage MS2 JM2 Model
## 30 157 Bacteriophage MS2 Chick Model
## 31 158 Salmonella typhimurium phages JM2 Model
## 32 159 Salmonella typhimurium phages Chick Model
## 33 160 Salmonella typhimurium phages Chick Model
## 34 161 Salmonella typhimurium phages JM2 Model
## 35 162 Ascaris suum JM2 Model
## 36 163 Ascaris suum JM2 Model
## 37 164 Ascaris suum Chick Model
## 38 165 Ascaris eggs Chick Model
## 39 166 Ascaris eggs Chick Model
## 40 167 Ascaris eggs Chick Model
## 41 168 Ascaris eggs JM2 Model
## 42 169 Ascaris eggs JM2 Model
## 43 170 Ascaris eggs Chick Model
## 44 171 Ascaris eggs JM2 Model
## 45 172 Ascaris eggs JM2 Model
## 46 173 Escherichia Coli JM2 Model
## 47 174 Escherichia Coli JM2 Model
## 48 175 Escherichia Coli JM2 Model
## 49 176 MS2 coliphage Chick Model
## 50 177 MS2 coliphage JM2 Model
## 51 178 MS2 coliphage Chick Model
## 52 179 Ascaris JM2 Model
## 53 180 Ascaris Chick Model
## 54 181 Ascaris Chick Model
## 55 182 Ascaris JM2 Model
## 56 183 Ascaris Chick Model
## 57 184 Ascaris Chick Model
## 58 185 Ascaris JM2 Model
## 59 186 Ascaris JM2 Model
## 60 187 Ascaris Chick Model
## 61 188 Ascaris JM2 Model
## 62 189 Ascaris JM2 Model
## 63 190 Ascaris ova Chick Model
## 64 191 Ascaris ova Chick Model
## 65 192 Ascaris ova JM2 Model
## 66 193 Ascaris ova Chick Model
## 67 194 Ascaris ova JM2 Model
## 68 195 Ascaris ova JM2 Model
## 69 196 Ascaris eggs JM2 Model
## 70 197 Ascaris eggs JM2 Model
## 71 198 Ascaris eggs JM2 Model
## 72 199 Ascaris eggs JM2 Model
## 73 200 Ascaris eggs JM2 Model
## 74 201 Ascaris eggs JM2 Model
## 75 202 Fecal coliforms Chick Model
## 76 203 Streptococcus Chick Model
## 77 204 Salmonella Chick Model
## 78 205 Fecal coliforms Chick Model
## 79 206 Streptococcus Chick Model
## 80 207 Salmonella Chick Model
## 81 208 Fecal coliforms Chick Model
## 82 209 Streptococcus Chick Model
## 83 210 Salmonella Chick Model
## 84 211 Fecal coliforms Chick Model
## 85 212 Streptococcus Chick Model
## 86 213 Salmonella Chick Model
## 87 214 Fecal coliforms Chick Model
## 88 215 Streptococcus Chick Model
## 89 216 Salmonella Chick Model
## 90 217 Fecal coliforms Chick Model
## 91 218 Streptococcus Chick Model
## 92 219 Salmonella Chick Model
## 93 220 Fecal coliforms Chick Model
## 94 221 Streptococcus Chick Model
## 95 222 Salmonella Chick Model
## 96 223 Total coliform JM2 Model
## 97 224 Eschericia Coli Chick Model
## 98 225 Streptococcus JM2 Model
## 99 226 Sulfur reducing clostridia Chick Model
## 100 227 Entamoeba cysts Chick Model
## 101 228 Ascaris eggs Chick Model
## 102 229 Escherichia coli Chick Model
## 103 230 Escherichia coli Chick Model
## 104 231 Escherichia coli Chick Model
## 105 232 Escherichia coli JM2 Model
## 106 233 Escherichia coli JM2 Model
## 107 234 Escherichia coli JM2 Model
## 108 235 Escherichia coli Chick Model
## 109 236 Escherichia coli Chick Model
## 110 237 Escherichia coli Chick Model
## 111 238 Enterococcus JM2 Model
## 112 239 Enterococcus JM2 Model
## 113 240 Enterococcus JM2 Model
## 114 241 Enterococcus JM2 Model
## 115 242 Enterococcus JM2 Model
## 116 243 Enterococcus JM2 Model
## 117 244 Enterococcus Chick Model
## 118 245 Enterococcus Chick Model
## 119 246 Enterococcus Chick Model
## 120 247 Enterococcus Chick Model
## 121 248 Enterococcus Chick Model
## 122 249 Enterococcus Chick Model
## 123 250 Escherichia coli Chick Model
## 124 251 Escherichia coli Chick Model
## 125 252 Escherichia coli Chick Model
## 126 253 MS2 coliphage JM2 Model
## 127 254 T4 coliphage JM2 Model
## 128 255 Adenovirus JM2 Model
## 129 256 Ascaris Chick Model
## 130 257 Ascaris Chick Model
## 131 258 Ascaris JM2 Model
## 132 259 Ascaris JM2 Model
## 133 260 Ascaris Chick Model
## 134 261 Ascaris JM2 Model
## 135 262 Ascaris JM2 Model
## 136 263 Ascaris JM2 Model
## 137 264 Ascaris Chick Model
## 138 265 Ascaris Chick Model
## 139 266 Ascaris Chick Model
## 140 267 Ascaris Chick Model
## 141 268 Salmonella spp Chick Model
## 142 269 Salmonella spp Chick Model
## 143 270 Salmonella spp Chick Model
## 144 271 Salmonella spp Chick Model
## 145 272 Salmonella spp Chick Model
## 146 273 Salmonella spp Chick Model
## 147 274 Salmonella spp Chick Model
## 148 275 Salmonella spp Chick Model
## 149 276 Salmonella spp Chick Model
## 150 277 Salmonella spp Chick Model
## 151 278 Salmonella spp Chick Model
## 152 279 Salmonella spp JM2 Model
## 153 280 Salmonella typhimurium phage 28B JM2 Model
## 154 281 Thermotolerant coliform JM2 Model
## 155 282 Enterococci JM2 Model
## 156 283 Salmonella senftenberg JM2 Model
## 157 284 Salmonella typhimurium phage 28B JM2 Model
## 158 285 Salmonella senftenberg JM2 Model
## 159 286 Thermotolerant coliform JM2 Model
## 160 287 Enterococci JM2 Model
## 161 288 Fecal coliforms Chick Model
## 162 289 Fecal coliforms JM2 Model
## 163 290 Fecal coliforms Chick Model
## 164 291 Fecal coliforms Chick Model
## 165 292 Escherichia coli JM2 Model
## 166 293 Enterococci JM2 Model
## 167 294 Enterococci JM2 Model
## 168 295 Escherichia coli Chick Model
## 169 296 Enterococci JM2 Model
## 170 297 Escherichia coli JM2 Model
## 171 298 Enterococci JM2 Model
## 172 299 Escherichia coli JM2 Model
## 173 300 Escherichia coli Chick Model
## 174 302 Escherichia coli JM2 Model
## 175 303 Escherichia coli Chick Model
## 176 304 Fecal coliforms Chick Model
## 177 305 Fecal coliforms JM2 Model
## 178 306 Fecal coliforms Chick Model
## 179 307 Fecal coliforms Chick Model
## 180 308 Escherichia coli Chick Model
## 181 309 Escherichia coli Chick Model
## 182 310 Escherichia coli Chick Model
## 183 311 Escherichia coli Chick Model
## 184 312 Escherichia coli Chick Model
## 185 313 Escherichia coli Chick Model
## 186 314 Total coliforms JM2 Model
## 187 315 Total coliforms Chick Model
## 188 316 Total coliforms Chick Model
## 189 317 Total coliforms JM2 Model
## 190 318 Total coliforms Chick Model
## 191 319 Total coliforms Chick Model
## 192 320 Salmonella spp. JM2 Model
## 193 321 Salmonella spp. Chick Model
## 194 322 Salmonella spp. Chick Model
## 195 323 Escherichia coli JM2 Model
## 196 324 Escherichia coli JM2 Model
## 197 325 Escherichia coli JM2 Model
## 198 326 Shigella sonnei JM2 Model
## 199 327 Shigella sonnei JM2 Model
## 200 328 Shigella flexneri Chick Model
## 201 329 Shigella sonnei JM2 Model
## 202 330 Shigella sonnei JM2 Model
## 203 331 Shigella flexneri JM2 Model
## 204 332 Rotavirus Chick Model
## 205 333 Rotavirus Chick Model
## 206 334 Rotavirus Chick Model
## 207 335 Rotavirus JM2 Model
## 208 336 Rotavirus Chick Model
## 209 337 Rotavirus Chick Model
## 210 338 Rotavirus Chick Model
## 211 339 Rotavirus Chick Model
## 212 340 Escherichia coli JM2 Model
## 213 341 Escherichia coli JM2 Model
## 214 342 Escherichia coli Chick Model
## 215 343 Escherichia coli JM2 Model
## 216 344 Escherichia coli Chick Model
## 217 345 Enterococcus faecalis Chick Model
## 218 346 Enterococcus faecalis Chick Model
## 219 347 Enterococcus faecalis Chick Model
## 220 348 Enterococcus faecalis JM2 Model
## 221 349 Enterococcus faecalis JM2 Model
## 222 350 Ascaris lumbricoides Chick Model
## 223 351 Ascaris lumbricoides JM2 Model
## 224 352 Ascaris lumbricoides JM2 Model
## 225 353 Ascaris lumbricoides JM2 Model
## 226 354 Ascaris lumbricoides JM2 Model
## 227 355 Escherichia coli JM2 Model
## 228 356 Escherichia coli JM2 Model
## 229 357 Escherichia coli JM2 Model
## 230 358 Escherichia coli JM2 Model
## 231 359 Escherichia coli JM2 Model
## 232 360 Escherichia coli JM2 Model
## 233 361 Escherichia coli Chick Model
## 234 362 Escherichia coli Chick Model
## 235 363 Cryptosporidium Chick Model
## 236 364 Cryptosporidium Chick Model
## 237 365 Giardia Chick Model
## 238 366 Giardia Chick Model
## 239 367 Fecal coliforms Chick Model
## 240 368 Fecal coliforms Chick Model
## 241 369 Cryptosporidium JM2 Model
## 242 370 Cryptosporidium JM2 Model
## 243 371 Cryptosporidium JM2 Model

## Produce figures and tables

This last chunk of code uses the data frame kPit (which has all of the results from fitting the models to data from each experiment) to produce figures and tables.

### Table 1

# sample size for k-value calcs based on log linear, then sample size for t99 calcs based on log linear, then the k values, then the estimated T99 values based on the log linear model
data.frame(rbind(c(paste(length(kPit[kPit$microbial_group=="Virus",]$k)," (",length(kPit[kPit$microbial_group=="Virus"&kPit$maxLRV>=1,]$t99),")",sep=""),
 -quantile(kPit[kPit$microbial_group=="Virus",]$k,c(0.5,0.95,0.05)),
 quantile(kPit[kPit$microbial_group=="Virus"&kPit$maxLRV>=1,]$t99,c(0.5,0.05,0.95))),
c(paste(length(kPit[kPit$microbial_group=="Bacteria",]$k)," (",length(kPit[kPit$microbial_group=="Bacteria"&kPit$maxLRV>=1,]$t99),")",sep=""),
 -quantile(kPit[kPit$microbial_group=="Bacteria",]$k,c(0.5,0.95,0.05)),
 quantile(kPit[kPit$microbial_group=="Bacteria"&kPit$maxLRV>=1,]$t99,c(0.5,0.05,0.95))),
c(paste(length(kPit[kPit$microbial_group=="Protozoa",]$k)," (",length(kPit[kPit$microbial_group=="Protozoa"&kPit$maxLRV>=1,]$t99),")",sep=""),
 -quantile(kPit[kPit$microbial_group=="Protozoa",]$k,c(0.5,0.95,0.05)),
 quantile(kPit[kPit$microbial_group=="Protozoa"&kPit$maxLRV>=1,]$t99,c(0.5,0.05,0.95))),
c(paste(length(kPit[kPit$microbial_group=="Helminth",]$k)," (",length(kPit[kPit$microbial_group=="Helminth"&kPit$maxLRV>=1,]$t99),")",sep=""),
 -quantile(kPit[kPit$microbial_group=="Helminth",]$k,c(0.5,0.95,0.05)),
 quantile(kPit[kPit$microbial_group=="Helminth"&kPit$maxLRV>=1,]$t99,c(0.5,0.05,0.95)))
))

## V1 X50. X95. X5. X50..1 X5..1
## 1 32 (31) 0.18885 0.02547 16.800765 29.8092087720436 0.940083546180488
## 2 147 (120) 0.9313 0.01164 242.79872 0.90902445630565 0.00242002305642991
## 3 8 (4) 0.00945 0.001605 0.03501 269.526297347728 125.84936663375
## 4 56 (23) 0.04165 5e-05 0.52 44.3065754116127 6.62139246819842
## X95..1
## 1 134.199331043184
## 2 72.4674672473823
## 3 320.697383842882
## 4 290.930499952965

### Table 2

data.frame(rbind(
 c(length(kPit[kPit$microbial_group=="Virus"&kPit$num>3&kPit$maxLRV>=1,]$t99jm),
 quantile(kPit[kPit$microbial_group=="Virus"&kPit$num>3&kPit$maxLRV>=1,]$k1,c(0.5,0.05,0.95),na.rm=T),
 quantile(kPit[kPit$microbial_group=="Virus"&kPit$num>3&kPit$maxLRV>=1,]$k2,c(0.5,0.05,0.95),na.rm=T),
 quantile(kPit[kPit$microbial_group=="Virus"&kPit$num>3&kPit$maxLRV>=1,]$t99jm,c(0.5,0.05,0.95),na.rm=T)),
 c(length(kPit[kPit$microbial_group=="Bacteria"&kPit$num>3&kPit$maxLRV>=1,]$t99jm),
 quantile(kPit[kPit$microbial_group=="Bacteria"&kPit$num>3&kPit$maxLRV>=1,]$k1,c(0.5,0.05,0.95),na.rm=T),
 quantile(kPit[kPit$microbial_group=="Bacteria"&kPit$num>3&kPit$maxLRV>=1,]$k2,c(0.5,0.05,0.95),na.rm=T),
 quantile(kPit[kPit$microbial_group=="Bacteria"&kPit$num>3&kPit$maxLRV>=1,]$t99jm,c(0.5,0.05,0.95),na.rm=T)),
 c(length(kPit[kPit$microbial_group=="Protozoa"&kPit$num>3&kPit$maxLRV>=1,]$t99jm),
 quantile(kPit[kPit$microbial_group=="Protozoa"&kPit$num>3&kPit$maxLRV>=1,]$k1,c(0.5,0.05,0.95),na.rm=T),
 quantile(kPit[kPit$microbial_group=="Protozoa"&kPit$num>3&kPit$maxLRV>=1,]$k2,c(0.5,0.05,0.95),na.rm=T),
 quantile(kPit[kPit$microbial_group=="Protozoa"&kPit$num>3&kPit$maxLRV>=1,]$t99jm,c(0.5,0.05,0.95),na.rm=T)),
 c(length(kPit[kPit$microbial_group=="Helminth"&kPit$num>3&kPit$maxLRV>=1,]$t99jm),
 quantile(kPit[kPit$microbial_group=="Helminth"&kPit$num>3&kPit$maxLRV>=1,]$k1,c(0.5,0.05,0.95),na.rm=T),
 quantile(kPit[kPit$microbial_group=="Helminth"&kPit$num>3&kPit$maxLRV>=1,]$k2,c(0.5,0.05,0.95),na.rm=T),
 quantile(kPit[kPit$microbial_group=="Helminth"&kPit$num>3&kPit$maxLRV>=1,]$t99jm,c(0.5,0.05,0.95),na.rm=T))
))

## V1 X50. X5. X95. X50..1 X5..1 X95..1 X50..2 X5..2
## 1 31 -7.190 -31.0500 17.725 4.920 1.485 17.870 29.367170 0.9675115
## 2 76 0.875 -78.3400 215.855 3.625 0.450 215.855 8.677702 0.1009299
## 3 3 -13.360 -13.6210 -6.547 3.130 2.104 3.310 220.666726 190.4310660
## 4 18 -8.675 -58.1235 -1.847 3.830 1.389 17.626 61.532533 12.3873031
## X95..2
## 1 187.9810
## 2 158.3257
## 3 328.6808
## 4 457.6740

### Figure 2

persistExc<-persist[!persist$experiment_id %in% kPit$experiment,]
persistInc<-persist[persist$experiment_id %in% kPit$experiment,]

plotFun<-function(persistanceData){
 fitV <- lm(-log10_reduction ~ time_days, data = persistanceData[persistanceData$microbial_group=="Virus" & persistanceData$matrix=="Fecal sludge" & persistanceData$time_days<200,])
 new_df <- persistanceData[persistanceData$microbial_group=="Virus" & persistanceData$matrix=="Fecal sludge" & persistanceData$time_days<200,]
 vi <- ggplot(new_df, aes(time_days, -log10_reduction)) +
 geom_point() +
 ylim(-8,0) + ylab("Log Reduction Value") +
 xlim(0,150) + xlab("Time (days)") +
 ggtitle("Viruses") +
 theme(plot.title = element_text(hjust=0.5)) +
 theme_bw() #+ geom_smooth(method=lm, se=TRUE)
 fitB <- lm(-log10_reduction ~ time_days, data = persistanceData[persistanceData$microbial_group=="Bacteria" & persistanceData$matrix=="Fecal sludge" & persistanceData$time_days<200,])
 new_df <- persistanceData[persistanceData$microbial_group=="Bacteria" & persistanceData$matrix=="Fecal sludge" & persistanceData$time_days<200,]
 ba <- ggplot(new_df, aes(time_days, -log10_reduction))+
 geom_point() +
 ylim(-8,0) + ylab("Log Reduction Value") +
 xlim(0,150) + xlab("Time (days)") +
 ggtitle("Bacteria") +
 theme(plot.title = element_text(hjust=0.5))+
 theme_bw()
 fitH <- lm(-log10_reduction ~ time_days, data = persistanceData[persistanceData$microbial_group=="Helminth" & persistanceData$matrix=="Fecal sludge" & persistanceData$time_days<200,])
 new_df <- persistanceData[persistanceData$microbial_group=="Helminth" & persistanceData$matrix=="Fecal sludge" & persistanceData$time_days<200,]
 he <- ggplot(new_df, aes(time_days, -log10_reduction))+
 geom_point() +
 ylim(-8,0) + ylab("Log Reduction Value") +
 xlim(0,150) + xlab("Time (days)") +
 ggtitle("Helminths") +
 theme(plot.title = element_text(hjust=0.5))+
 theme_bw()
 fitP <- lm(-log10_reduction ~ time_days, data = persistanceData[persistanceData$microbial_group=="Protozoa" & persistanceData$matrix=="Fecal sludge" & persistanceData$time_days<200,])
 new_df <- persistanceData[persistanceData$microbial_group=="Protozoa" & persistanceData$matrix=="Fecal sludge" & persistanceData$time_days<200,]
 pr <- ggplot(new_df, aes(time_days, -log10_reduction))+
 geom_point() +
 ylim(-8,0) + ylab("Log Reduction Value") +
 xlim(0,150) + xlab("Time (days)") +
 ggtitle("Protozoa") +
 theme(plot.title = element_text(hjust=0.5))+
 theme_bw()
grid.arrange(vi,ba,pr,he,nrow = 2,widths = c(2,2))
}
plotFun(persistInc) # Plots Figure 2


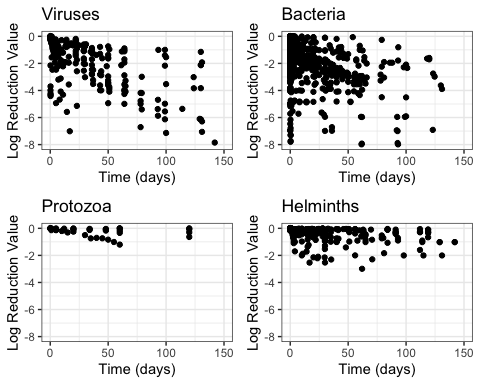


### Figure 3

kPit$tk<-log(-kPit$k)
kPit$tk1<-sqrt(log(kPit$k1+150))
kPit$tk2<-sqrt(log(kPit$k2+10))
par(mfrow=c(1,3))
myData<-kPit[kPit$temp<50&kPit$temp>0,c("tk","k1","tk2","urine","urea","additive")]
myData$urine<-factor(myData$urine)
a<-ggplot(myData,aes(urine,tk))+geom_boxplot(aes(fill=urine))+theme_bw()+scale_fill_grey(start=0,end=.9)
b<-ggplot(myData,aes(urea,tk))+geom_boxplot(aes(fill=urea))+theme_bw()+scale_fill_grey(start=0,end=.9)
c<-ggplot(myData,aes(additive,tk))+geom_boxplot(aes(fill=additive))+theme_bw()+scale_fill_grey(start=0,end=.9)
grid.arrange(a,b,c,nrow=1,widths=c(1,1,2))


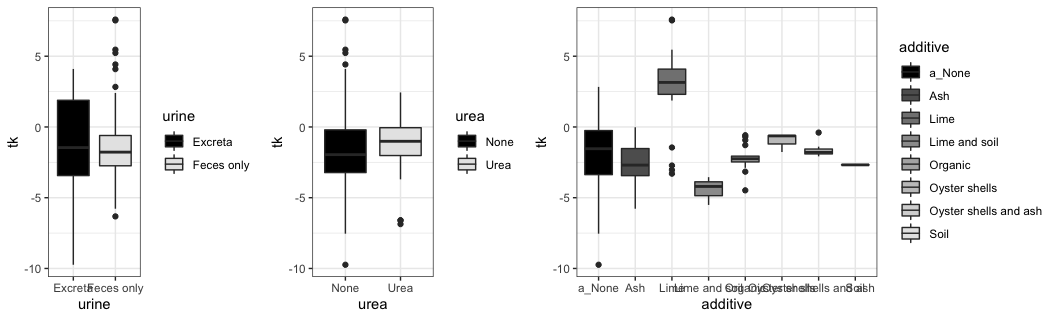


a<-ggplot(myData,aes(urine,k1))+geom_boxplot(aes(fill=urine),outlier.shape=NA)+theme_bw()+scale_fill_grey(start=0,end=.9)+coord_cartesian(ylim=c(-100,50))
b<-ggplot(myData,aes(urea,k1))+geom_boxplot(aes(fill=urea),outlier.shape=NA)+theme_bw()+scale_fill_grey(start=0,end=.9)+coord_cartesian(ylim=c(-100,50))
c<-ggplot(myData,aes(additive,k1))+geom_boxplot(aes(fill=additive),outlier.shape=NA)+theme_bw()+scale_fill_grey(start=0,end=.9)+coord_cartesian(ylim=c(-100,50))
grid.arrange(a,b,c,nrow=1,widths=c(1,1,2))


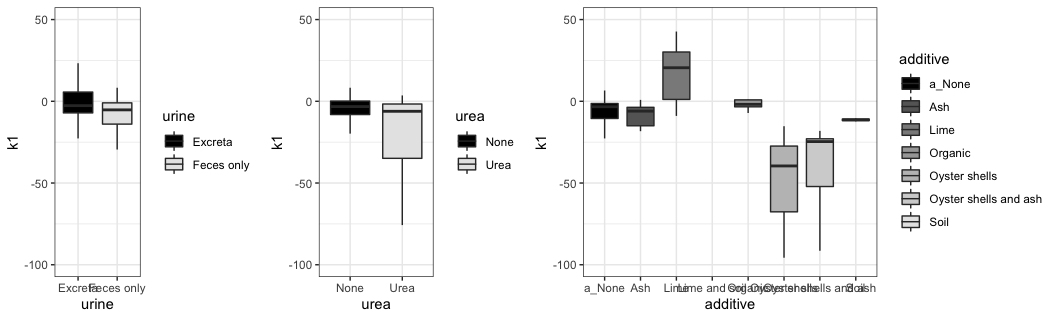


a<-ggplot(myData,aes(urine,tk2))+geom_boxplot(aes(fill=urine),outlier.shape=NA)+theme_bw()+scale_fill_grey(start=0,end=.9)+coord_cartesian(ylim=c(1.5,2))
b<-ggplot(myData,aes(urea,tk2))+geom_boxplot(aes(fill=urea),outlier.shape=NA)+theme_bw()+scale_fill_grey(start=0,end=.9)+coord_cartesian(ylim=c(1.5,2))
c<-ggplot(myData,aes(additive,tk2))+geom_boxplot(aes(fill=additive),outlier.shape=NA)+theme_bw()+scale_fill_grey(start=0,end=.9)+coord_cartesian(ylim=c(1.5,2))
grid.arrange(a,b,c,nrow=1,widths=c(1,1,2))


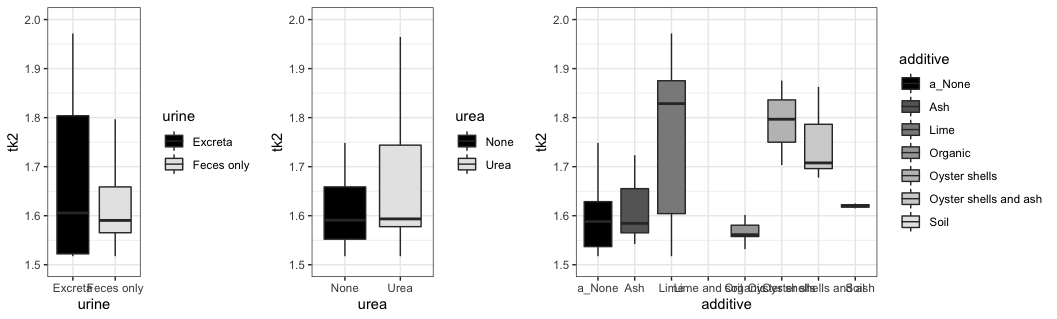


### Table 1

kPit$lk<-log(-kPit$k)

## Warning in log(-kPit$k): NaNs produced

fit.final<-lm(lk~factor(microbial_group)+pH+temp+moisture+factor(urine)+factor(urea)+factor(additive),data=kPit[kPit$temp<50,]) #Regression
summary(fit.final)

##
## Call:
## lm(formula = lk ~ factor(microbial_group) + pH + temp + moisture +
## factor(urine) + factor(urea) + factor(additive), data = kPit[kPit$temp <
## 50, ])
##
## Residuals:
## Min 1Q Median 3Q Max
## -3.8146 -0.8802 0.0259 0.9639 3.9028
##
## Coefficients:
## Estimate Std. Error t value Pr(>|t|)
## (Intercept) -7.114015 1.241884 -5.728 4.28e-08 ***
## factor(microbial_group)Helminth -3.095314 0.319952 -9.674 < 2e-16 ***
## factor(microbial_group)Protozoa -3.545962 0.809523 -4.380 2.02e-05 ***
## factor(microbial_group)Virus -0.853137 0.373070 -2.287 0.023391 *
## pH 0.336058 0.124312 2.703 0.007534 **
## temp 0.059870 0.013559 4.416 1.75e-05 ***
## moisture 0.020508 0.005455 3.759 0.000231 ***
## factor(urine)Feces only 1.198454 0.374606 3.199 0.001633 **
## factor(urea)Urea 0.580519 0.359224 1.616 0.107867
## factor(additive)Ash -0.891555 0.527245 -1.691 0.092603 .
## factor(additive)Lime 3.609997 0.520643 6.934 7.39e-11 ***
## factor(additive)Lime and soil -1.678775 1.183292 -1.419 0.157735
## factor(additive)Organic -1.534367 0.506761 -3.028 0.002832 **
## factor(additive)Oyster shells -0.612545 1.013803 -0.604 0.546481
## factor(additive)Oyster shells and ash -0.347010 0.662433 -0.524 0.601044
## factor(additive)Soil 0.311357 1.221094 0.255 0.799033
## ---
## Signif. codes: 0 '***' 0.001 '**' 0.01 '*' 0.05 '.' 0.1 ' ' 1
##
## Residual standard error: 1.661 on 177 degrees of freedom
## (9 observations deleted due to missingness)
## Multiple R-squared: 0.6674, Adjusted R-squared: 0.6392
## F-statistic: 23.67 on 15 and 177 DF, p-value: < 2.2e-16

### Figure S4

par(mfrow=c(3,2),mar=c(4,4,2,2))
hist(kPit[kPit$temp<50&kPit$temp>0,]$k,breaks=100)
hist(kPit[kPit$temp<50&kPit$temp>0,]$tk,breaks=100)
hist(kPit[kPit$temp<50&kPit$temp>0,]$k1,breaks=100)
hist(kPit[kPit$temp<50&kPit$temp>0&kPit$k1>-20&kPit$k1<20,]$tk1,breaks=100)
hist(kPit[kPit$temp<50&kPit$temp>0,]$k2,breaks=100)
hist(kPit[kPit$temp<50&kPit$temp>0&kPit$k1<50,]$tk2,breaks=100)


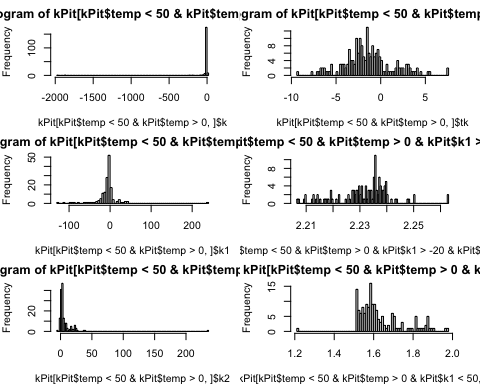


### Figure S5

myData<-kPit[kPit$temp<50&kPit$temp>0,c("microbial_group","k","k1","k2","urine","urea","additive")]
a<-ggplot(myData,aes(microbial_group,k))+geom_boxplot(aes(fill=microbial_group),outlier.shape=NA)+theme_bw()+scale_fill_grey(start=0,end=.9)+coord_cartesian(ylim=c(-15,1))
b<-ggplot(myData,aes(microbial_group,k1))+geom_boxplot(aes(fill=microbial_group),outlier.shape=NA)+theme_bw()+scale_fill_grey(start=0,end=.9)+coord_cartesian(ylim=c(-35,10))
c<-ggplot(myData,aes(microbial_group,k2))+geom_boxplot(aes(fill=microbial_group),outlier.shape=NA)+theme_bw()+scale_fill_grey(start=0,end=.9)+coord_cartesian(ylim=c(-10,30))
grid.arrange(a,b,c,nrow=1,widths=c(1,1,1))

## Warning: Removed 40 rows containing non-finite values (stat_boxplot).

## Warning: Removed 40 rows containing non-finite values (stat_boxplot).


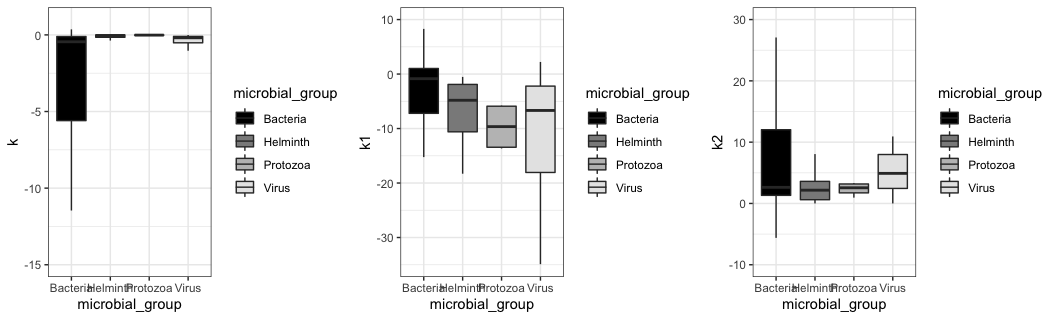


### Figure S6

myData<-kPit[kPit$temp<50&kPit$temp>0,c("tk","k1","tk2","temp","pH","moisture")]
names(myData)<-c("ln(k) (1/days)","k1","tk2","Temperature (°C)","pH","moisture")
par(mfrow=c(1,2))
chart.Correlation(kPit[,c("tk","k1","tk2","temp","pH","moisture")],histogram=TRUE)


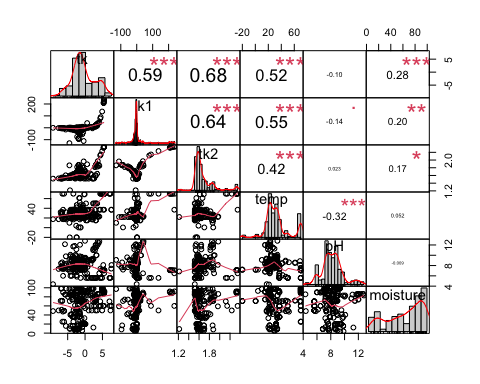


chart.Correlation(myData,histogram=TRUE)


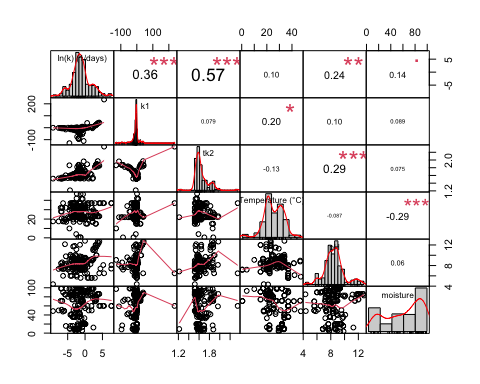


### Figure S7

fit.final<-lm(lk~factor(microbial_group)+pH+temp+moisture+factor(urine)+factor(urea)+factor(additive),data=kPit)
par(mfrow=c(2,3))
plot(fit.final,which=1:6)


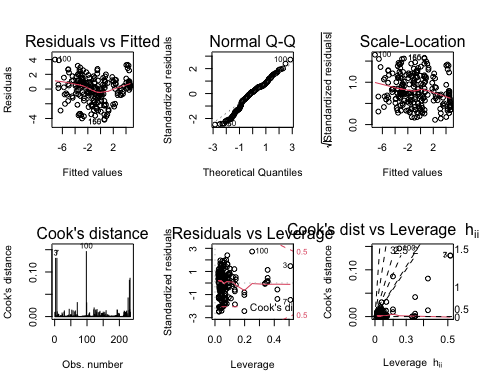


1. Musaazi, I.G., Verbyla, M.E., McLoughlin, S., Murphy, H., 2020. Modeling pathogen inactivation in fecal sludge along the sanitation service chain: A systematic review and meta-analysis methodology. PROSPERO, National Institute for Health Research, Record No. CRD42020167254. Available at: www.crd.york.ac.uk/prospero/display_record.php?ID=CRD42020167254. [↑](#footnote-ref-1)
